# Metastable dynamics emerge from local excitatory–inhibitory homeostasis in the cortex at rest

**DOI:** 10.1162/netn_a_00460

**Published:** 2025-07-29

**Authors:** Francisco Páscoa dos Santos, Paul F. M. J. Verschure

**Affiliations:** Eodyne Systems SL, Barcelona, Spain; Universitat Pompeu Fabra, Barcelona, Spain; Donders Institute for Brain, Cognition and Behavior, Radboud University, Nijmegen, The Netherlands

**Keywords:** Excitatory–inhibitory homeostasis, Cortical networks, Metastability, Homeostatic plasticity, Large-scale modeling, Complexity

## Abstract

The dynamics of the human cortex are highly metastable, driving the spontaneous exploration of network states. This metastability depends on circuit-level edge-of-bifurcation dynamics, which emerge from firing-rate control through multiple mechanisms of excitatory–inhibitory (E–I) homeostasis. However, it is unclear how these contribute to the metastability of cortical networks. We propose that individual mechanisms of the E–I homeostasis contribute uniquely to the emergence of resting-state dynamics and test this hypothesis in a large-scale model of the human cortex. We show that empirical connectivity and dynamics can only be reproduced when accounting for multiple mechanisms of the E–I homeostasis. More specifically, while the homeostasis of excitation and inhibition enhances metastability, the regulation of intrinsic excitability ensures moderate synchrony, maximizing functional complexity. Furthermore, the modulation bifurcation modulation by the homeostasis of excitation and intrinsic excitability compensates for strong input fluctuations in connector hubs. Importantly, this only occurs in models accounting for local gamma oscillations, suggesting a relationship between E–I balance, gamma rhythms, and metastable dynamics. Altogether, our results show that cortical networks self-organize toward maximal metastability through the multifactor homeostasis of E–I balance. Therefore, the benefits of combining multiple homeostatic mechanisms transcend the circuit level, supporting the metastable dynamics of large-scale cortical networks.

## Author Summary

Experimental studies have consistently shown that cortical circuits maintain a precise homeostasis of excitatory–inhibitory (E–I) balance, thereby optimizing local dynamics. While it is well established that multiple homeostatic mechanisms are involved in this local regulation, it remains unclear how each contributes to the large-scale dynamics of cortical networks. This study presents evidence that, through the E–I homeostasis, the cortex can self-organize toward a regime of highly complex and metastable spontaneous dynamics. Crucially, we demonstrate that this results from the synergistic action of multiple homeostatic mechanisms. Our findings advance our understanding of the E–I homeostasis as a process of self-organization, demonstrating its key role in the maintenance of metastable dynamics in large-scale cortical networks.

## INTRODUCTION

**Complex systems**, such as the human brain, show rich collective dynamics beyond the behavior of their units (Mitchell, 2011; Sporns, 2022; Turkheimer et al., 2022). One of such collective behaviors in cortical networks is **metastability**, underlying the spontaneous exploration of the state-space of network configurations (Freeman & Holmes, 2005; Haken, 2006; Haldeman & Beggs, 2005; Hancock et al., 2023; Raichle, 2011, 2015; Tognoli & Kelso, 2014) with a pivotal role in cognitive function (Alderson, Bokde, Kelso, Maguire, & Coyle, 2018; Alderson et al., 2018; Cabral et al., 2017; Hancock et al., 2022; Hellyer, Scott, Shanahan, Sharp, & Leech, 2015). That said, the key ingredients underlying the **emergence** of metastable dynamics are not well understood. The **connectome** can have a pivotal role, either due to its intrinsic inhomogeneity (Tognoli & Kelso, 2014) or conduction delays between cortical areas (Cabral et al., 2022; Hancock et al., 2023), supporting intrinsic activity patterns with high complexity (Arsiwalla & Verschure, 2016; Sporns, Tononi, & Edelman, 2002; Zamora-López, Chen, Deco, Kringelbach, & Zhou, 2016). However, it has also been suggested that a necessary condition for the emergence of metastability is that the units making up the system are poised near a phase transition or **bifurcation** point (Freeman, 2005; Freeman & Holmes, 2005), underlying the rich patterns of activity observed in the resting-state neocortex (Arsiwalla & Verschure, 2016, 2017; Cabral et al., 2014; Deco & Jirsa, 2012; Deco, Kringelbach, Jirsa, & Ritter, 2017; Freeman, 2005; Freeman & Holmes, 2005; Schirner, Kong, Yeo, Deco, & Ritter, 2022). More specifically, when multiple small-scale phase transitions co-occur, they can self-reinforce and lead to the emergence of collective activity patterns (Freeman, 2005). Therefore, while the emergence of metastable dynamics requires the interaction between cortical areas, which is constrained by the connectome, local circuit dynamics must also be key in its genesis. For this reason, we hypothesize that **excitatory–inhibitory (E–I) homeostasis**, which regulates edge-of-bifurcation dynamics in local cortical circuits (Beggs & Timme, 2012; Freeman, 2005; Ma, Turrigiano, Wessel, & Hengen, 2019; Poil, Hardstone, Mansvelder, & Linkenkaer-Hansen, 2012; Poil, van Ooyen, & Linkenkaer-Hansen, 2008; Páscoa dos Santos & Verschure, 2025), could be a fundamental principle for understanding the cortex and its collective dynamics (Sporns, 2022; Turkheimer, Leech, Expert, Lord, & Vernon, 2015).

There is extensive evidence that the excitatory and inhibitory inputs received by cortical pyramidal (PY) neurons are subject to a tight balance (Douglas, Martin, & Whitteridge, 1991; Mariño et al., 2005; Okun & Lampl, 2008; Wehr & Zador, 2003; Xue, Atallah, & Scanziani, 2014), essential for a myriad of operations performed by cortical networks (Beggs & Timme, 2012; Sadeh & Clopath, 2021; van Vreeswijk & Sompolinsky, 1996; Vogels, Sprekeler, Zenke, Clopath, & Gerstner, 2011). For this reason, cortical neurons are equipped with mechanisms of the E–I homeostasis, which contribute to the maintenance of E–I balance in the face of perturbations in activity (Gainey & Feldman, 2017; Maffei & Turrigiano, 2008; Turrigiano, 2011; Turrigiano, Leslie, Desai, Rutherford, & Nelson, 1998). These mechanisms include synaptic scaling of excitatory (Maffei, Nelson, & Turrigiano, 2004; Maffei & Turrigiano, 2008; Turrigiano et al., 1998) and inhibitory synapses (Keck et al., 2013; Maffei et al., 2004; Maffei & Turrigiano, 2008; Xue et al., 2014) and the adaptation of intrinsic excitability (Desai, Rutherford, & Turrigiano, 1999; Maffei & Turrigiano, 2008; Wen & Turrigiano, 2021). Furthermore, recent results suggest that the conjugation of multiple forms of the E–I homeostasis not only ensures stable firing rates but can also regulate the activity of local cortical circuits toward criticality (Ma et al., 2019; Wen & Turrigiano, 2024), providing a mechanism for the control of edge-of-bifurcation dynamics at the circuit level (Páscoa dos Santos & Verschure, 2025).

In that context, several computational studies have explored how local E–I balance ensures flexible collective dynamics (Hellyer, Jachs, Clopath, & Leech, 2016; Naskar, Vattikonda, Deco, Roy, & Banerjee, 2021) and the emergence of **functional connectivity** (FC) networks (Abeysuriya et al., 2018; Deco et al., 2014; Naskar et al., 2021), particularly in the ultra-slow dynamics characteristic of BOLD signals (Castaldo et al., 2023). In addition, E–I homeostasis can also be a mechanism of **self-organization**, driving the recovery of FC following lesions to the connectome (Chakraborty, Saha, Deco, Banerjee, & Roy, 2023; Páscoa dos Santos & Verschure, 2022; Vattikonda, Surampudi, Banerjee, Deco, & Roy, 2016), and the reemergence of functional properties such as modularity (Páscoa dos Santos, Vohryzek, & Verschure, 2023). However, a common caveat of such studies is the exclusive focus on the homeostasis of inhibitory synapses, neglecting a possible role for the combined action of multiple mechanisms of homeostasis in the maintenance of circuit dynamics (Ma et al., 2019; Wen & Turrigiano, 2024). Importantly, our results suggest that multiple mechanisms of homeostasis not only regulate stable firing rates but also the distance to the bifurcation of local circuits (Páscoa dos Santos & Verschure, 2025), although it is not clear how this shapes large-scale dynamics. Moreso, the homeostasis of inhibition alone is not sufficient for the recovery of collective dynamics in lesioned networks (Páscoa dos Santos et al., 2023), suggesting the need for other homeostatic mechanisms.

Here, we aim to investigate the hypothesis that relying on distinct mechanisms of homeostasis at the circuit level is essential to support the emergence of large-scale metastable dynamics in the human cortex. To do this, we study how large-scale models with local E–I homeostasis reproduce empirical FC and FC dynamics (FCD), synchrony, metastability, and the complexity of functional networks. Given our focus on metastability, it should be stressed that our main objective is not to study models optimized to be maximally metastable, but to investigate if a large-scale state of heightened metastability can be a consequence of the maintenance of E–I balance at the local level. That said, we explore the dependence of collective dynamics not only on E–I homeostasis but also on the specific mechanisms employed to achieve it. In this context, our results demonstrate that the only models able to reproduce empirical FC, FCD, network dynamics, and complexity are the ones accounting for the combined homeostasis of excitation, inhibition, and intrinsic excitability. Finally, we also show that networks with these mechanisms of homeostasis can reorganize to recover not only functional networks but also metastable dynamics following focal lesions. Therefore, our results demonstrate that the maintenance of E–I balance not only underlies the emergence of metastable spontaneous dynamics in the cortex but is also a robust mechanism of self-organization, especially when based on multiple complementary mechanisms of the E–I homeostasis.

## METHODS

### Structural Connectivity (SC) Data

To approximate the anatomical connections between brain areas, we use a normative connectome from 32 healthy participants (mean age = 31.5 years old ± 8.6, 14 females) generated as part of the Human Connectome Project (HCP; https://www.humanconnectome.org). In this work, we focus on the cortical regions of the AAL atlas, yielding a 78 × 78 matrix. Tract lengths between brain regions were also extracted to be used in the computation of conduction delays between network nodes. For more detailed information, refer to Castaldo et al. (2023).

### BOLD fMRI Data

To analyze network dynamics and FC properties in empirical data, we use resting-state BOLD time series from 99 healthy unrelated subjects from the HCP dataset. Data were processed using the pipeline described in Castaldo et al. (2023), yielding 99 time series of size 78 areas × 1200 TR, roughly corresponding to 14.5 min of data for each subject (TR = 0.72 s).

### Large-Scale Model

To model the activity of individual cortical regions, comprising excitatory (*r^E^*) and inhibitory (*r^I^*) neural masses, we make use of the Wilson–Cowan model (Cowan, Neuman, & Van Drongelen, 2016; Wilson & Cowan, 1972):

$$\begin{array}{ll} \tau^{E}\frac{r_{i}^{E}\left(t\right)}{dt} & =-r_{i}^{E}\left(t\right)+F^{E}\left(G^{E}c^{EE}r_{i}^{E}\left(t\right)-c^{EI}r_{i}^{I}\left(t\right)+G^{E}I_{i}^{\mathrm{ext}}+\xi \left(t\right)\right)\\ \tau^{I}\frac{r_{i}^{I}\left(t\right)}{dt} & =-r_{i}^{I}\left(t\right)+F^{I}\left(c^{IE}r_{i}^{E}\left(t\right)+\xi \left(t\right)\right) \end{array}$$ (1)

Where *c^EE^* represents the recurrent excitatory coupling, *c^EI^* represents the coupling from inhibitory to excitatory populations, and *c^IE^* represents the excitatory coupling unto inhibitory populations. *G^E^* is a parameter introduced in Páscoa dos Santos and Verschure (2025), allowing for the scaling of all excitatory inputs to the excitatory neural mass (i.e., recurrent excitation and *I^ext^*). The default values of each parameter can be consulted in Supporting Information Table S1. Unless stated otherwise, *ξ*(*t*) represents Gaussian noise with mean 0 and variance 0.01. Here, $I_{i}^{\mathrm{ext}}$ describes the incoming input to each node *i* from the rest of the network and can be written as follows:

$$I_{i}^{\mathrm{ext}}=C\sum_{j=1}^{N}W_{ij}r_{j}^{E}\left(t-\tau_{ij}\right)+P$$ (2)

where *C* is a scaling factor referred to as global coupling, *W*_*ij*_ is the SC matrix, and *τ_ij_* represents the conduction delay between nodes *i* and *j*. Here, similarly to Abeysuriya et al. (2018), Castaldo et al. (2023), and Páscoa dos Santos et al. (2023), *P* represents a background input imparted on the nodes and is set at 0.31 so that the default uncoupled node is close to the Hopf bifurcation. The time constants *τ^E^* and *τ^I^* were tuned so that the uncoupled node oscillates at ∼40 Hz. Furthermore, we define *F*^*E*/*I*^(*x*) as the input–output function of the excitatory and inhibitory populations, respectively, using the following equation:

$$F^{E/I}\left(x\right)=\frac{1}{1+e^{-\frac{x-\mu^{E/I}}{\sigma^{E/I}}}}$$ (3)

### E–I Homeostasis

As opposed to the common approach to E–I homeostasis (Abeysuriya et al., 2018; Castaldo et al., 2023; Deco et al., 2014; Hellyer et al., 2016; Naskar et al., 2021; Páscoa dos Santos et al., 2023), we do not implement dynamical homeostasis. Instead, we follow the framework developed in Páscoa dos Santos and Verschure (2025) to mathematically estimate the parameter values that ensure the maintenance of mean excitatory activity at a target firing rate (*ρ*) for different levels of input (*I^ext^*; Hengen, Lambo, Van Hooser, Katz, & Turrigiano, 2013; Ma et al., 2019), in line with the governing rules of the E–I homeostasis (Turrigiano, 2011). That said, to compute the homeostatic parameters of nodes embedded in a network, it is necessary to provide an estimate of *I^ext^* for each node. To do this, we rely on the fact that E–I homeostasis works in timescales much slower than neural dynamics (Gainey & Feldman, 2017; Turrigiano, 2011; Wen & Turrigiano, 2024), ensuring timescale separation. Therefore, to compute the homeostatic parameters, we consider the average external input received by each node at the homeostatic set point, when the average activity across the network corresponds to *ρ*, leading to the following expression:

$$\begin{array}{ll} {\langle I_{i}^{\mathrm{ext}}\rangle }_{t} & ={\langle P\rangle }_{t}+{\langle C\sum_{j}W_{ij}r_{j}^{E}\rangle }_{t}\\ {\langle I_{i}^{\mathrm{ext}}\rangle }_{t} & =P+C\sum_{j}W_{ij}{\langle r_{j}^{E}\rangle }_{t}\\ \langle I_{i}^{\mathrm{ext}}\rangle & =P+C\rho \sum_{j}W_{ij} \end{array}$$ (4)

Here, we explore the behavior of models with the following distinct mechanisms of the E–I homeostasis, based on experimental results (Desai et al., 1999; Maffei et al., 2004; Maffei & Turrigiano, 2008; Turrigiano, 2011; Turrigiano et al., 1998; Wen & Turrigiano, 2021, 2024) and described in detail in Páscoa dos Santos and Verschure (2025):

- *G^E^* homeostasis: synaptic scaling of excitatory synapses in PY neurons.
- *c^EI^* homeostasis: synaptic scaling of inhibitory synapses in PY neurons.
- *μ^E^* homeostasis: plasticity of the intrinsic excitability of PY neurons, modulating the “firing threshold” (i.e., *μ^E^*) of *F^E^*(*x*).
- *μ^E^* + *σ^E^* homeostasis: plasticity of the intrinsic excitability of PY neurons, modulating both the “firing threshold” (*μ^E^*) and “slope” (*σ^E^*) of *F^E^*(*x*) in a synergistic manner.
- *G^E^* + *c^EI^* homeostasis: synaptic scaling of excitatory and inhibitory synapses in PY neurons.
- *G^E^* + *c^EI^* + *μ^E^* homeostasis: synaptic scaling of excitatory and inhibitory synapses and intrinsic excitability of PY neurons, implemented at the level of *μ^E^*.
- *G^E^* + *c^EI^* + *μ^E^* + *σ^E^* homeostasis: synaptic scaling of excitatory and inhibitory synapses and intrinsic excitability of PY neurons, implemented at the level of *μ^E^* and *σ^E^*.

The equations to compute the set-point parameters corresponding to each form of homeostasis can be consulted in SI Methods.

### Hemodynamic Model

From the raw activity of the excitatory populations, we extract simulated BOLD signals by using a forward hemodynamic model (Friston, Mechelli, Turner, & Price, 2000; Friston, Harrison, & Penny, 2003), described in detail in Páscoa dos Santos et al. (2023). This model represents the coupling between model activity and blood vessel diameter, which affects blood flow, blood volume, and deoxyhemoglobin content. After passing model activity through the hemodynamic model, the output is downsampled to a sampling period of 0.72 s to align simulated signals with empirical data.

### Network Validity

For each type of homeostasis and combination of hyper-parameters (*C, ρ*, and mean delay), we evaluate if the homeostatic set point—that is, a mean firing rate of *ρ* for all nodes—can be maintained across the network. To do this, we run 15-s simulations and compute the mean firing rate of each node in the last 10 s of the simulation. We consider the given network fixed point to be invalid if there is any node in the network for which the mean firing rate differs from *ρ* by more than 1% (i.e., $\frac{|\langle r^{E}\rangle -\rho |}{\rho }>0.01$). The dynamical behavior of invalid models is examined in detail in SI Text. While it is possible to evaluate the stability of a network analytically (see Ghosh, Rho, McIntosh, Kötter, & Jirsa, 2008), the characteristic polynomial of our model (from which the eigenvalues can be extracted) does not have a closed-form solution due to the highly nonlinear nature of the Wilson–Cowan equations. For this reason, we base our exploration of model validity on the analysis of the numerical solution of the system.

### Model Optimization

We perform model optimization by exploring all combinations of the global coupling (*C*), mean delay, and target firing rate (*ρ*) within the following ranges of variation: *C*: [0, 10], mean delay: [0, 40] ms, and *ρ*: [0.05, 0.2]. Within these ranges, we selected 19 logarithmically spaced values for *C*, 16 values for *ρ* in steps of 0.01, and 41 mean delays in steps of 1 ms. For each simulation, we initialize the model with the homeostatic parameters corresponding to the combination of *C* and *ρ* and record model activity for 30 min. To evaluate model performance against empirical data, we use the following properties of FC.

▪  **Static FC**: 78 × 78 matrix of correlations between BOLD time series across all network nodes. Modeled FC matrices were compared with group-averaged empirical FC by computing the correlation coefficient (*r*_*FC*_) and mean squared error (*MSE*_*FC*_) between their upper triangular elements.
**FCD**: matrix of correlations between the upper triangular part of FC matrices computed in windows of 80 samples with 80% overlap (Deco et al., 2017, 2021). Model results are compared with empirical data by computing the Kolmogorov–Smirnov (KS) distance between the distributions of values in the respective FCD matrices (*KS*_*FCD*_).

BOLD signals are filtered between 0.008 and 0.08 Hz before computing FC and FCD. In addition, we compute a cross-feature fitting score as $\frac{r_{FC}-{MSE}_{FC}-{KS}_{FCD}-S_{\min}}{S_{\max}-S_{\min}}$ to evaluate the performance of models in simultaneously reproducing static and dynamic properties of empirical FC (Castaldo et al., 2023; Páscoa dos Santos et al., 2023). *S*_*max*_ = 1 represents the maximum value of *r*_*FC*_ − *MSE*_*FC*_ − *KS*_*FCD*_ (1–0–0) while *S*_*min*_ = −4.0024 represents its minimum (−1–2.0024–1). The value of 2.0024 represents the maximum possible MSE between an FC matrix and our empirical FC matrix, considering that FC can only vary between −1 and 1. With this expression, we obtain a cross-feature fitting score normalized between 0 and 1, indicating how well models represent FC and FCD.

All simulations were performed by solving the system’s equations numerically using the Euler method with an integration time step of 0.2 ms.

### Synchrony and Metastability

To evaluate the magnitude of synchrony and the degree of dynamic switching between network states (i.e., metastability), we compute the Kuramoto Order Parameter (KOP; Kuramoto, 1975; Shanahan, 2010) of BOLD timeseries, filtered between 0.008 and 0.08 Hz. More specifically, the KOP (*R*(*t*) in Equation 5) represents the degree of phase alignment within a set of coupled oscillators at a given point in time and can be calculated as follows:

$$R\left(t\right)e^{i\Phi \left(t\right)}=\frac{1}{N}\sum_{n=1}^{N}e^{i\theta_{n}\left(t\right)}$$ (5)

where *θ_n_*(*t*) represents the instantaneous Hilbert phase of node *n* at time *t*. Synchrony and metastability are defined, respectively, as the mean and standard deviation of *R*(*t*) over time.

### Functional Complexity

Previous studies have demonstrated that the architecture of structural networks has a significant impact on the complexity of the functional interactions between nodes (Arsiwalla & Verschure, 2016; Sporns et al., 2002; Zamora-López et al., 2016). To explore this, we measure the complexity of simulated FC matrices using the method introduced in Zamora-López et al. (2016). In short, we quantify complexity by measuring how strongly the distribution of FC weights deviates from an equivalent uniform distribution using the following formula:

$$C=1-\frac{1}{C_{M}}\sum_{m=1}^{M}|p_{m}\left({FC}_{ij}\right)-\frac{1}{M}|$$ (6)

where *M* represents the number of bins used to evaluate the FC distribution. The term $C_{M}=2\frac{m-1}{m}$ is added for normalization, corresponding to the deviation of a Dirac-delta function from the uniform distribution. Therefore, FC complexity is 0 when all entries of the FC matrix are the same and 1 when they are uniformly distributed. While there are other methods to evaluate the complexity of a matrix, Zamora-López et al. (2016) demonstrate that their formulation is more robust to changes in bin sizes, compared with other methods quantifying the entropy or variance of FC matrices. Here, we use a bin size of 0.05.

### Replication of Analysis Using the Wong–Wang Model

To investigate the effects of the E–I homeostasis on slow local dynamics, we employ the reduced Wong–Wang model of cortical populations (Deco et al., 2014). In this model, instead of simulating fast α-Amino-3-hydroxy-5-methyl-4-isoxazolepropionic acid (AMPA) synapses, excitatory synapses have a considerably slower decay time (100 ms), consistent with the timescale of N-methyl-D-aspartate (NMDA) synapses. The model dynamics are described as follows:

$$\begin{array}{ll} I_{i}^{E} & =G^{E}W_{E}I_{0}+G^{E}w_{+}J_{\text{NMDA}}S_{i}^{E}+{CG}^{E}J_{\text{NMDA}}\sum_{j}W_{ij}S_{j}^{E}-J_{\text{GABA}}S_{i}^{I}\\ I_{i}^{I} & =W_{I}I_{0}+J_{\text{NMDA}}S_{i}^{I}-J_{\text{GABA}}S_{i}^{I}\\ r_{i}^{E} & =F^{E}\left(I_{i}^{E}\right)\\ r_{i}^{I} & =F^{I}\left(I_{i}^{I}\right)\\ \frac{{dS}_{i}^{E}\left(t\right)}{dt} & =-\frac{S_{i}^{E}}{\tau^{E}}+\left(1-S_{i}^{E}\right)\gamma r_{i}^{E}+\xi \left(t\right)\\ \frac{{dS}_{i}^{I}\left(t\right)}{dt} & =-\frac{S_{i}^{I}}{\tau^{I}}+r_{i}^{I}+\xi \left(t\right) \end{array}$$ (7)

where $I_{i}^{E/I}$ represents the total input to excitatory or inhibitory neuronal population *i*, $r_{i}^{E/I}$ represents its firing rate, and $S_{i}^{E/I}\left(t\right)$ represents an average synaptic gating variable. In this case, we have also added the parameter *G^E^*, which allows for the simultaneous modulation of all excitatory synapses unto the excitatory neuronal population. The function *ξ*(*t*) represents the additive Gaussian noise with mean 0 and variance 0.01 (nA). *H*^*E*/*I*^(*t*) represents the input–output function of neuronal populations and is written as follows:

$$F^{E/I}\left(x\right)=\frac{a^{E/I}x-b^{E/I}}{1-\exp\left(-d^{E/I}\left(a^{E/I}x-b^{E/I}\right)\right)}$$ (8)

The parameter values, adapted from Deco et al. (2014) and their physiological meaning are described in detail in Supporting Information Table S3. Importantly, this model does not consider the dynamics of AMPA synapses and *τ^E^* = 100 ms (the time constant of excitatory synapses) represents the slow decay time of NMDA (Deco et al., 2014). Therefore, in line with Deco et al. (2014) and given the focus of the Wong–Wang model on slower dynamics, interareal conduction delays are neglected in this model.

Similarly to the Wilson–Cowan model, we derived the equations allowing for the mathematical estimation of the steady-state value of each model parameter corresponding to the different empirically based mechanisms of homeostasis. More specifically, we investigate the following forms of the E–I homeostasis:

- *G^E^* homeostasis: synaptic scaling of excitatory synapses in PY neurons.
- *J*_*GABA*_ homeostasis: synaptic scaling of inhibitory synapses in PY neurons.
- *b^E^* homeostasis: plasticity of the intrinsic excitability of PY neurons, modulating the “firing threshold” (i.e., *b^E^*) of *F^E^*(*x*).
- *a^E^* homeostasis: plasticity of the intrinsic excitability of PY neurons, the “slope” of (*σ^E^*) of *F^E^*(*x*) in a synergistic manner. In this case, since changing *a^E^* has a minimal impact on the value of *F^E^*(*x*) at 0, we employ this form of homeostasis to simulate the plasticity of intrinsic excitability described in Desai et al. (1999); Nataraj, Le Roux, Nahmani, Lefort, and Turrigiano (2010); and Wen and Turrigiano (2021). For more details, refer to Supporting Information Figure S9.
- *G^E^* + *J*_*GABA*_ homeostasis: synaptic scaling of excitatory and inhibitory synapses in PY neurons.
- *G^E^* + *J*_*GABA*_ + *b^E^* homeostasis: synaptic scaling of excitatory and inhibitory synapses and intrinsic excitability of PY neurons, implemented at the level of *b^E^*.
- *G^E^* + *J*_*GABA*_ + *a^E^* homeostasis: synaptic scaling of excitatory and inhibitory synapses and intrinsic excitability of PY neurons, implemented at the level of *a^E^*.

For more details on the derivation of the mathematical expressions and the effects of the E–I homeostasis at the circuit level, refer to the SI Methods.

Similarly to the procedure employed for the Wilson–Cowan model, we perform model optimization by running one simulation for each combination of the global coupling (*C*) and target firing rate (*ρ*) within the following ranges of variation: *C*: [0, 1] and *ρ*: [1, 10] Hz. Within these ranges, we selected 25 logarithmically spaced values for *C* and 10 values for *ρ*. For each simulation, we initialize the model with the homeostatic parameters corresponding to the combination of *C* and *ρ* and record model activity for 30 min. In Deco et al. (2014), the target firing rate is fixed at 3.0631 Hz; the model is considered to have reached the homeostatic fixed point when the mean firing rate is between 2.63 and 3.55 Hz, corresponding to an interval of ∼15% around the target. Therefore, here, we consider a simulation valid when the error between mean firing rates and the target is smaller than 15% for all nodes in the network.

Finally, we detect the optimal combination of *C* and *ρ* maximizing the fitting score for each form of homeostasis. To be able to statistically compare models, we run 10 simulations with each combination of optimal parameters and compute the respective cross-feature fitting score and metastability.

### Effects of Noise

To explore the impact of noise in our model, we run simulations of the network with homeostasis of *G^E^, c^EI^*, and *μ^E^* +*σ^E^* with optimal parameters (*C* = 3.59, *ρ* = 0.12) and across all mean delays yielding valid simulations. For each simulation, we vary the variance of Gaussian noise (*ξ*(*t*)), corresponding to 29 logarithmically spaced values between 10^−5^ and $10^{-\frac{1}{3}}$. We then analyze model performance, synchrony, metastability, and functional complexity across all models with varying levels of noise.

### Lesion Simulation Protocol

Following the framework applied in Páscoa dos Santos et al. (2023), we investigate the effects of focal lesions in model dynamics and functional interactions by following the following protocol. Using the model with all mechanisms of homeostasis at the optimal point (*C* = 3.59; *ρ* = 0.12), we compute the homeostatic parameters by using the methodology from Páscoa dos Santos and Verschure (2025; SI Methods). From the balanced model, we extract 30 min of the BOLD signal corresponding to the activity in the prelesion (i.e., healthy) period. Then, we apply a structural lesion by removing all connections to and from a chosen node and extract 30 min of activity corresponding to the early acute period of stroke recovery. We then compute the new homeostatic parameters, allowing for the restoration of E–I balance in the lesioned connectome, using the prelesion homeostatic values as $G_{0}^{E}$, $c_{0}^{EI}$, and $\mu_{0}^{E}$ (SI Methods for the equations). From the rebalanced model, we extract 30 min of model activity corresponding to the chronic period. We follow this protocol for all possible single-node lesions and extract the following metrics from the healthy, acute, and chronic simulations: difference of FC from baseline, FC-SC correlation, FC modularity, FC complexity, synchrony, metastability, and FCD distributions. For a more detailed description of how each feature is computed, refer to SI methods. While, in the main text, we present the effects of lesions on models with the combination of all mechanisms of homeostasis, we also report the effects of lesions on models with *G^E^* and *G^E^* +*c^EI^* homeostasis (Supporting Information Figures S27–S28).

### Code

All simulations and analyses were run using in-house scripts in Python. The code can be consulted in https://gitlab.com/francpsantos/wc_network_homeostasis.

## RESULTS

**Figure 1.**
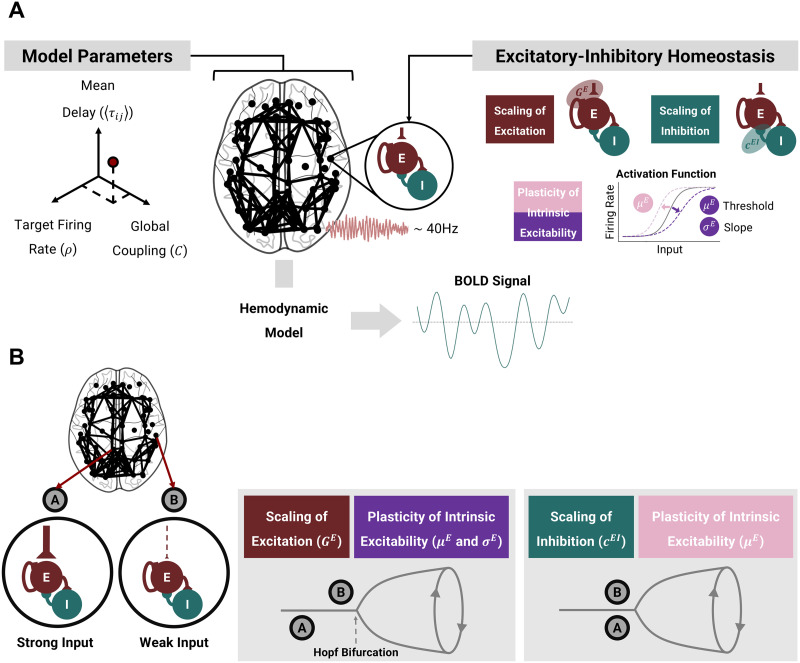
Model architecture and local E–I homeostasis. (A) To simulate the resting-state dynamics of the cortex, we built a large-scale model based on the healthy human connectome. Cortical areas are modeled with the Wilson–Cowan model of coupled E–I populations, tuned to oscillate at 40 Hz. Based on experimental studies, we implement distinct forms of the local E–I homeostasis to maintain the mean excitatory activity at a target firing rate (*ρ*) based on the scaling of excitatory synapses (*G^E^*), inhibitory synapses (*c^EI^*), or the plasticity of intrinsic excitability, either through the adaptation of the firing threshold (*μ^E^*) or the threshold and slope (*μ^E^* and *σ^E^*) of the input–output function of neural populations. In addition, we explore models with different combinations of these homeostatic mechanisms and of the free parameters (global coupling *C*, target firing rate [*ρ*], and mean delay). We extract simulated BOLD signals for further analysis and benchmarking against empirical resting-state recordings. (B) Effects of distinct homeostatic mechanisms on local circuit dynamics of strongly and weakly connected nodes. Certain forms of the E–I homeostasis, such as the scaling of excitation and the plasticity of excitability slope (*σ^E^*) and threshold (*μ^E^*) modulate the distance to the bifurcation of local cortical circuits, so that areas with a strong average input are poised further away from the bifurcation. Conversely, for the homeostasis of inhibition (*c^EI^*) or excitability threshold (*μ^E^*), the distance to the bifurcation mostly depends on the target firing rate (*ρ*). For more information, consult Páscoa dos Santos and Verschure (2025).

To study how E–I homeostasis impacts the resting-state dynamics of the neocortex, we constructed a large-scale model considering some of its key features together with the local regulation of E–I balance (Figure 1A). The dynamics of cortical areas are modeled with the Wilson–Cowan model of coupled E–I populations with **gamma-band oscillations** (∼40 Hz). Long-range connectivity and conduction delays between areas follow empirical SC data derived from diffusion-tensor imaging. Critically, we implement multiple empirically identified mechanisms of the local E–I homeostasis (Páscoa dos Santos & Verschure, 2025; Turrigiano, 2011; Wen & Turrigiano, 2024), which ensure that activity remains close to a target firing rate (*ρ*) by regulating excitatory and inhibitory synapses and the intrinsic excitability of excitatory populations in each cortical area (Páscoa dos Santos & Verschure, 2025; Figure 1A).

Critically, homeostasis is not implemented dynamically in this work. More specifically, we take advantage of the separation of timescales between the fast circuit dynamics and the ultraslow homeostatic mechanisms (Turrigiano, 2011; Wen & Turrigiano, 2024) to estimate analytically the parameter values corresponding to the homeostatic fixed point—that is, the parameters allowing for the maintenance of the target firing rate (*ρ*) across all nodes. This mathematical estimation allows for the models to be initialized with the homeostatic values of each relevant parameter, making the simulations more tractable and saving simulation time. For a more detailed derivation of the methods, consult Páscoa dos Santos and Verschure (2025). Here, to compute the local parameters of nodes embedded in the large-scale network, we estimate the external input received by each node from the rest of the network, assuming the mean firing rate to be *ρ* in all cortical areas (see the Methods section).

Importantly, our implementation of the Wilson–Cowan model behaves as a Hopf bifurcation between a fixed point of noisy activity and sustained gamma oscillations. That said, it is relevant to stress that the distinct mechanisms of homeostasis have specific effects on the local dynamics of the model (Figure 1B; Páscoa dos Santos & Verschure, 2025). More specifically, the scaling of excitation and the plasticity of the excitability slope (*σ^E^*) and threshold (*μ^E^*) modulate circuit dynamics so that circuits with stronger input are poised farther away from the bifurcation than nodes with weaker inputs. Conversely, the plasticity of inhibition (*c^EI^*) or the firing threshold (*μ^E^*) do not affect the distance to the bifurcation, which depends only on the target firing rate (Figure 1B). Furthermore, when combining multiple homeostatic mechanisms, their effects on edge-of-bifurcation dynamics are also compounded (Supporting Information Figure S1). For a more detailed exploration of the effects of each homeostatic mechanism on node dynamics, refer to Páscoa dos Santos and Verschure (2025).

To optimize model performance, we explore different combinations of free parameters, namely, the local target firing rate (*ρ*); the global coupling (*C*), which scales the SC; and the mean conduction delay, which depends on the length of white-matter tracts and the conduction velocity. For each simulation, we first verify the validity of the model by evaluating if the difference between mean local firing rates and the target (*ρ*) does not exceed 1%. We then only retain valid models—that is, where firing rates can be maintained close to the target—for further analysis. For a detailed exploration of model validity, refer to the Supporting Information SI Text and Supporting Information Figures S2–S11. Finally, we extract simulated BOLD signals from valid networks by using the Balloon–Windkessel hemodynamic model (see the Methods section) and analyze network dynamics based on the BOLD time series.

### Modulation of Distance to Bifurcation at Circuit Level Optimizes Fit to Empirical Data

We start our analysis by studying how distinct forms of homeostasis, based on the plasticity of excitation, inhibition, and intrinsic excitability (see the Methods section and Figure 1), shape the performance of our model in reproducing empirical FC and FCD obtained from resting-state BOLD signals, which are strongly modulated by E–I balance (Castaldo et al., 2023; Páscoa dos Santos et al., 2023). We simulate the activity of a large-scale model of the neocortex with different combinations of free parameters (*C, ρ*, and mean delay) and mechanisms of homeostasis (Figure 1). For each simulation, we compare empirical and simulated FC matrices and FCD distributions and extract a cross-feature fitting score to evaluate model performance. The score is calculated as $\frac{r_{FC}-{MSE}_{FC}-{KS}_{FCD}-S_{\min}}{S_{\max}-S_{\min}}$, where *r*_*FC*_ is the Pearson’s correlation between empirical and simulated FC matrices, *MSE*_*FC*_ is their mean squared error, *KS*_*FCD*_ is the Kolmogorov–Smirnov distance between FCD distributions, and *S*_*max*_/*S*_*min*_ is the maximum/minimum of *r*_*FC*_ − *MSE*_*FC*_ − *KS*_*FCD*_. With this, we obtain a cross-feature fitting score between 0 and 1, reflecting how well models represent the features of interest (see the Methods section for more details). Furthermore, our results remain similar when employing a different method to compute the cross-feature fitting score (Supporting Information Figure S12).

**Figure 2.**
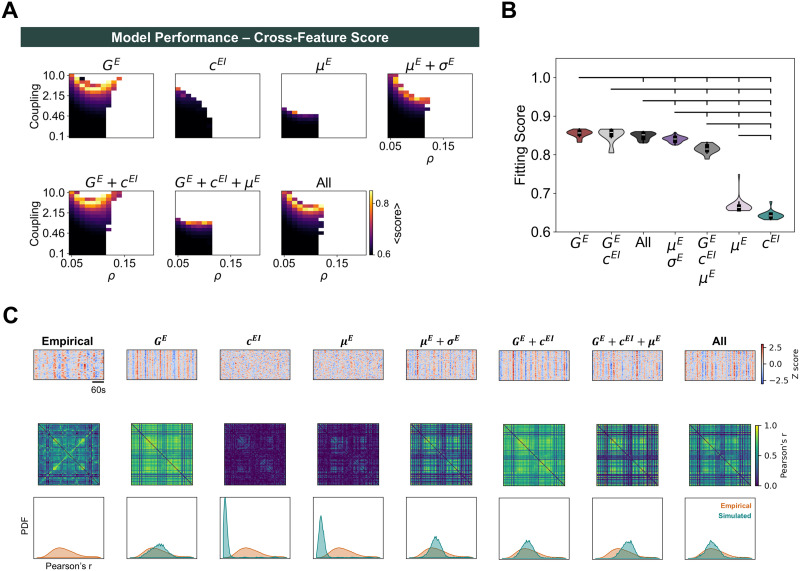
Cross-feature model performance for different homeostatic mechanisms. (A) Parameter spaces representing the cross-feature score for each combination of *C* and *ρ*, averaged across mean delays. Blank spaces represent combinations of *C* and *ρ* for which the homeostatic set point is not valid (i.e., mean firing rates differ from *ρ* by more than 1% in at least one cortical area). (B) Comparison between cross-feature fitting scores at the optimal point of each mechanism of homeostasis. Distributions correspond to simulations with optimal *C* and *ρ* and all values of mean delay yielding a valid network solution. Brackets indicate a significant difference, with *p* < 0.05 from a Mann–Whitney *U* test. All *p* values were FDR corrected. (C) Examples of network activity (top), FC matrices (middle), and FCD distributions (bottom) from empirical data and optimized models with distinct combinations of homeostatic mechanisms. In each plot, we present 6 min of band-pass filtered and *Z*-scored activity across all cortical areas. Each example from the optimal models represents a simulation with the optimal combination of *C* and *ρ* and a mean delay of 4 ms.

First, it is relevant to point out that the main parameters impacting model performance are *C* and *ρ*, while the mean delay of interareal conduction does not have a strong impact, provided it is long enough to avoid widespread pathological synchronization (Supporting Information Figures S13–S16 for the full parameter spaces). Therefore, we present the performance scores for each combination of *C* and *ρ*, averaged across mean delays (Figure 2A). For each mechanism of homeostasis, we observed a narrow region in the parameter space where empirical resting-state FC and FCD emerge. To compare the optimal performance of each model, we select the combination of *C* and *ρ* yielding the highest fitting score while ensuring that simulations are valid for at least 10 different values of mean delay. Although conduction delays do not strongly impact the fitting score (Supporting Information Figures S13–S16), we enforce this constraint to ensure that models are robust to changes in conduction velocity without losing the ability to maintain stable firing rates. That said, we present the distribution of cross-feature fitting scores across mean delays for the optimal point of each model (Figure 2B, Table 1) and examples of activity, FC, and FCD distributions from empirical and simulated data at the optimal point of each model (Figure 2C). The best scores are obtained for models with *G^E^* homeostasis (0.855 ± 0.009) and *G^E^* +*c^EI^* homeostasis (0.851 ± 0.019), followed by the homeostasis of *G^E^, c^EI^*, and *μ^E^* +*σ^E^* (i.e., all; 0.849 ± 0.009). The values of *r*_*FC*_, *MSE*_*FC*_, and *KS*_*FCD*_ corresponding to each optimal score can be consulted in Table 1. While the performance of the model with *G^E^* homeostasis was significantly better than the one with all mechanisms of homeostasis (*p* = 0.020; Mann–Whitney *U* test, false discovery rate [FDR] correction), the effect size is not substantial (Cohen’s *d* = 0.281). Furthermore, the optimal target firing rates for the three best models (*G^E^*: 0.12; *G^E^* +*c^EI^*: 0.1; all: 0.12) are close to the bifurcation between damped and sustained oscillations (Supporting Information Figure S1), in line with theoretical (Cabral et al., 2014; Deco et al., 2017; Freeman, 2005) and experimental (Ma et al., 2019) results suggesting that the neocortex operates at the edge-of-bifurcation.

**Table 1.** Optimal parameters, fitting scores, dynamics, and complexity for each mechanism of the E–I homeostasis in comparison with empirical data

Homeostatic mechanism	*C*	*ρ*	Score	*r*_*FC*_	*MSE*_*FC*_	*KS*_*FCD*_
*G^E^*	5.99	0.12	0.86 ± 0.01	0.4 ± 0.01	0.061 ± 0.010	0.16 ± 0.04
*c^EI^*	2.15	0.05	0.64 ± 0.01	0.28 ± 0.02	0.158 ± 0.088	0.91 ± 0.04
*μ^E^*	0.46	0.09	0.67 ± 0.02	0.31 ± 0.02	0.139 ± 0.005	0.84 ± 0.08
*μ^E^* +*σ^E^*	3.59	0.07	0.84 ± 0.01	0.43 ± 0.02	0.056 ± 0.002	0.18 ± 0.03
*G^E^* +*c^EI^*	7.74	0.11	0.85 ± 0.02	0.48 ± 0.04	0.088 ± 0.031	0.14 ± 0.05
*G^E^* +*c^EI^* +*μ^E^*	1.00	0.10	0.82 ± 0.01	0.42 ± 0.01	0.063 ± 0.002	0.28 ± 0.05
All (*G^E^* +*c^EI^* +*μ^E^* +*σ^E^*)	3.59	0.12	0.85 ± 0.01	0.47 ± 0.02	0.046 ± 0.003	0.18 ± 0.04
Homeostatic mechanism	*C*	*ρ*	Synchrony	Metastability	Complexity	
*G^E^*	5.99	0.12	**0.661** ± **0.027**	0.20 ± 0.009	**0.640** ± **0.017**	
*c^EI^*	2.15	0.05	**0.203** ± **0.021**	**0.100** ± **0.007**	**0.393** ± **0.025**	
*μ^E^*	0.46	0.09	**0.253** ± **0.017**	**0.116** ± **0.009**	**0.436** ± **0.025**	
*μ^E^* +*σ^E^*	3.59	0.07	0.507 ± 0.015	**0.173** ± **0.008**	0.767 ± 0.021	
*G^E^* +*c^EI^*	7.74	0.11	**0.707** ± **0.041**	0.203 ± 0.011	**0.565** ± **0.052**	
*G^E^* +*c^EI^* +*μ^E^*	1.00	0.10	0.533 ± 0.014	**0.171** ± **0.007**	**0.797** ± **0.020**	
All (*G^E^* +*c^EI^* +*μ^E^* +*σ^E^*)	3.59	0.12	0.580 ± 0.021	0.191 ± 0.008	0.750 ± 0.020	
Empirical	–	–	0.537 ± 0.112	0.194 ± 0.025	0.754 ± 0.069	

Fitting scores correspond to $\frac{r_{FC}-{MSE}_{FC}-{KS}_{FCD}-S_{\min}}{S_{\max}-S_{\min}}$, where *r*_*FC*_ is the Pearson’s correlation between empirical and simulated FC matrices, *MSE*_*FC*_ is their mean squared error, *KS*_*FCD*_ is the Kolmogorov–Smirnov distance between FCD distributions, and *S*_*max*_/*S*_*min*_ is the maximum/minimum of *r*_*FC*_ − *MSE*_*FC*_ − *KS*_*FCD*_. Values are presented as mean ± *SD*. Values in bold represent a significant difference from empirical data (*p* < 0.05, Mann–Whitney *U* test, FDR correction).

Our results indicate that models with E–I homeostasis can reproduce the emergence of empirical networks and their dynamics at the ultra-slow timescales characteristic of resting-state BOLD, depending on the interaction between global coupling (*C*) and target firing rate (*ρ*). In addition, empirical data are best approximated when accounting for mechanisms of homeostasis that modulate the bifurcation point (i.e., the ones that modulate *G^E^* or *σ^E^*; Páscoa dos Santos & Verschure, 2025), so that areas with stronger inputs are poised farther away from the bifurcation (Figure 1B and Supporting Information Figure S1). This effect compensates for the higher input fluctuations in strongly connected nodes (Supporting Information Figure S17), ensuring that such nodes are not constantly entering the regime of sustained oscillations. Therefore, even though this effect leads to heterogeneous distances to the bifurcation across cortical areas, we argue that our results still align with the edge-of-bifurcation theory of brain dynamics (Arsiwalla & Verschure, 2016, 2017; Cabral et al., 2014; Deco & Jirsa, 2012; Deco et al., 2017; Freeman, 2005; Freeman & Holmes, 2005; Schirner et al., 2022) when input fluctuations are accounted for. More importantly, this bifurcation modulation, observed in models with the homeostasis of excitation (*G^E^*) and excitability slopes (*σ^E^*; Figure 1B and Supporting Information Figure S1), is essential for the emergence of FC and FCD (Figure 2).

### Resting-State FC Emerges in Networks With Metastable Dynamics

Given that the metastability of large-scale cortical networks is shaped by circuit-level edge-of-bifurcation dynamics (Deco et al., 2017; Freeman, 2005; Freeman & Holmes, 2005; Naskar et al., 2021), controlled by E–I homeostasis (Páscoa dos Santos & Verschure, 2025; Wen & Turrigiano, 2024), it is relevant to explore how distinct mechanisms of homeostasis shape the collective dynamics of the resting-state cortex. To do this, we measure the synchrony and metastability (see the Methods section) of the simulated BOLD signals of models under different homeostatic mechanisms and combinations of *C* and *ρ*. Again, the conduction delays do not significantly impact either synchrony or metastability (Supporting Information Figures S18–S19), and, for this reason, we present results averaged across mean delays.

**Figure 3.**
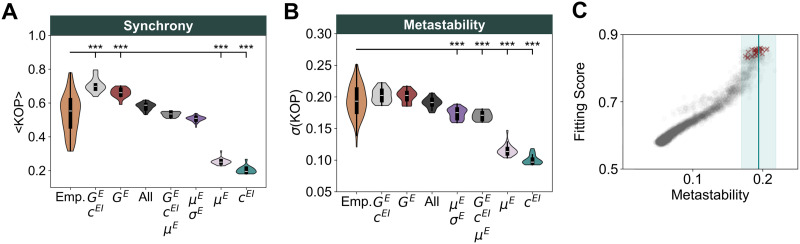
Networks with multiple mechanisms of homeostasis reproduce empirical network dynamics. (A) Comparison between synchrony in empirical data and models at the optimal point (best cross-feature fitting score) for each mechanism of homeostasis. Simulated distributions correspond to models with optimal *C* and *ρ* and all values of mean delay yielding a valid solution. Brackets represent a significant difference between models and empirical data from a Mann–Whitney *U* test (*p < 0.05; **p < 0.01; ***p < 0.001). All *p* values were FDR corrected. (B) Same as A, for metastability. (C) Relationship between fitting score and metastability in the model with homeostasis of excitation, inhibition, and intrinsic excitability (*μ^E^* and *σ^E^*). Gray dots show the results of all simulations while red crosses represent simulations at the optimal point (*C* = 3.59, *ρ* = 0.12). The vertical bar and shaded area display the mean and standard deviation of metastability in empirical BOLD signals.

We observe that synchrony generally follows increases in *C* and *ρ*, reaching a maximum before the networks become unstable (Supporting Information Figure S20). To explore how synchrony is shaped by E–I homeostasis, we compare the optimal models of each mechanism of homeostasis (i.e., best cross-feature fit) with empirical data (Figure 3A, Table 1). While some of the models that best reproduce empirical FC and FCD generally display moderate levels of synchrony, models based on the homeostasis of *G^E^* +*c^EI^* (0.707 ± 0.041) or *G^E^* (0.661 ± 0.027) show significantly higher synchrony than empirical resting-state BOLD dynamics (0.537 ± 0.112; *p* < 0.001, Mann–Whitney *U* test, FDR corrected). In contrast, networks based on the combination of all mechanisms of homeostasis, which reproduce empirical FC and FCD to a similar degree (Figure 2B), show levels of synchrony similar to empirical data (0.580 ± 0.021; *p* = 0.108; Mann–Whitney *U* test, FDR corrected).

Regarding metastability, our results reveal a region of maximal metastability determined by the interaction between *C* and *ρ* (Supporting Information Figure S21), coinciding with the region of optimal fit to empirical data (Figure 2A). Furthermore, the mechanisms of homeostasis that best fit empirical FC and FCD also show levels of metastability close to empirical values (0.194 ± 0.025; Figure 3B, Table 1). More specifically, we found enhanced metastability in models with the homeostasis of *G^E^* +*c^EI^* (0.203 ± 0.011), *G^E^* (0.201 ± 0.009), and the combination of all mechanisms of the E–I homeostasis (0.191 ± 0.008), with no significant difference from empirical values (*G^E^* +*c^EI^: p* = 0.254; *G^E^: p* = 0.254; All: *p* = 0.521; Mann–Whitney *U* test, FDR corrected). Importantly, empirical levels of both synchrony and metastability can only be reproduced when including all mechanisms of homeostasis (Figure 3 and Supporting Information Figure S22). In addition, we found a pronounced positive correlation between the metastability of models and their fitting scores, not only in the model with all mechanisms of homeostasis (Figure 3C) but as a general principle (Supporting Information Figure S23). Therefore, our results confirm previous studies proposing that the resting-state dynamics of cortical networks might correspond to a regime of maximal metastability (Deco et al., 2017; Naskar et al., 2021; Yang, Shew, Roy, & Plenz, 2012).

In summary, our analysis of network dynamics suggests that empirical FC networks and dynamics emerge more prominently in networks with moderate synchrony and high metastability, which can only be replicated when combining three mechanisms of the E–I homeostasis (*G^E^, c^EI^*, and *μ^E^* +*σ^E^*). More importantly, our results show that, through the homeostatic regulation of firing rates, cortical networks self-organize toward a regime of maximal metastability where the empirical resting-state networks emerge.

### Functional Complexity Is Shaped by E–I Balance, Metastability, and Connectome Topology

The complexity of spontaneous activity in the human brain is strongly related to the topology of its structural networks (Arsiwalla & Verschure, 2016; Sporns et al., 2002; Zamora-López et al., 2016). However, the architecture of the connectome is likely not the only source of complexity in cortical networks. Given the relevance of the E–I homeostasis at both the local and global scales, determining how local circuits engage in collective dynamics, E–I balance might be key in the emergence of complex functional interactions between cortical areas. Therefore, we study how different mechanisms of the E–I homeostasis impact FC complexity, using the methodology presented in Zamora-López et al. (2016; see the Methods section).

**Figure 4.**
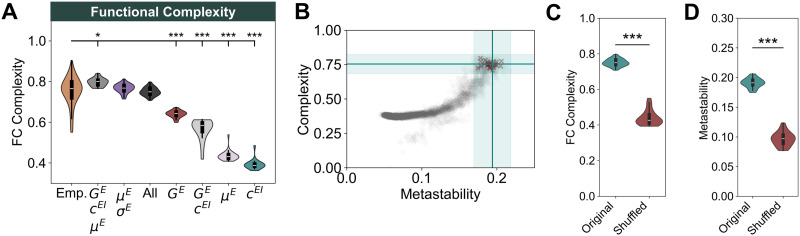
Functional complexity reflects E–I homeostasis, metastability, and connectome topology. (A) Comparison between functional complexity in empirical data and models at the optimal point (best cross-feature fit) for each mechanism of homeostasis. Distributions correspond to models with optimal *C* and *ρ* and all values of mean delay yielding a valid system. Brackets represent a significant difference between models and empirical data from a Mann–Whitney *U* test (*p < 0.05; **p < 0.01; ***p < 0.001). All *p* values were FDR corrected. (B) Relationship between complexity and metastability in the model with homeostasis of excitation, inhibition, and intrinsic excitability (*μ^E^* and *σ^E^*). Gray dots show the results of all simulations while red crosses represent simulations at the optimal point (*C* = 3.59, *ρ* = 0.12). The horizontal and vertical bars and shaded areas display the mean and standard deviation of complexity and metastability, respectively, in empirical BOLD signals. (C) Comparison between FC complexity in models with the original and shuffled structural connectomes and homeostasis of *G^E^, c^EI^, μ^E^*, and *σ^E^*. Simulations were performed with *C* = 3.59 and *ρ* = 0.12 and all mean delays yielding valid solutions. Asterisks represent the significance of a Mann–Whitney *U* test between samples (***p < 0.001). (D) Same as C, for metastability.

Our results on FC complexity suggest that, for all mechanisms of homeostasis, it is maximized in the region of the parameter space corresponding to heightened metastability and optimal fit to empirical FC and FCD (Supporting Information Figures S24 and S25). Comparing complexity in the models that best fit empirical FC and FCD data (Figure 4A, Table 1), our results indicate that the combined homeostasis of *G^E^, c^EI^*, and *μ^E^* (0.797 ± 0.020) maximizes FC complexity, although the resulting values are significantly higher than in empirical data (*p* = 0.014; Mann–Whitney *U* test, FDR corrected). Conversely, some of the homeostatic mechanisms with the best fit to empirical FC and FCD are associated with levels of complexity significantly lower than empirical values (*G^E^*: 0.640 ± 0.017, *p* < 0.001; *G^E^* +*c^EI^*: 0.565 ± 0.052, *p* < 0.001; Mann–Whitney *U* test, FDR-corrected). However, models relying on the combination of all forms of homeostasis reach levels of FC complexity (0.750 ± 0.020; *p* = 0.026) consistent with empirical data. Therefore, accounting for multiple mechanisms of homeostasis is necessary to reproduce not only synchrony and metastability but also functional complexity. Importantly, we found a strong correlation between metastability and complexity, not only for the model with all mechanisms of homeostasis (Figure 4B) but also across models (Supporting Information Figure S23). This suggests that heightened metastability of resting-state brain dynamics (Deco et al., 2017; Naskar et al., 2021) results in highly complex activity patterns that reflect on the variability of FC. More importantly, this requires the combined action of multiple mechanisms of the E–I homeostasis at the circuit level.

Having investigated the relationship between E–I homeostasis and complexity we compare complexity in models with the empirical and shuffled structural connectomes to assert the relevance of network topology (Arsiwalla & Verschure, 2016; Sporns et al., 2002; Zamora-López et al., 2016). Since shuffling preserves the distribution of edge weights, the complexity of the original and shuffled structural matrices is the same (Supporting Information Figure S26). That said, our results show that the complexity of FC in models with the empirical connectome is significantly higher than those with its shuffled counterpart (original: 0.750 ± 0.020; shuffled: 0.439 ± 0.042; *p* < 0.001; Mann–Whitney *U* test; Figure 4C). Therefore, in line with Arsiwalla and Verschure (2016); Sporns et al. (2002); and Zamora-López et al. (2016), the complexity of functional interactions also depends on the topology of SC. However, we point out that, given the relationship between metastability and FC complexity, this result could be an effect of decreased metastability in models with a shuffled connectome (Figure 4D). Therefore, our results suggest that while E–I homeostasis maximizes metastability and complexity by regulating edge-of-bifurcation dynamics in local cortical circuits, the role of the structural connectome in constraining their interactions should not be overlooked.

In summary, our results indicate that the processes underlying the emergence of metastable dynamics also maximize the complexity of spontaneous activity in the cortex. More importantly, our results suggest that multiple mechanisms of homeostasis (modulating excitatory and inhibitory synapses and intrinsic excitability through *μ^E^* and *σ^E^*) are required to reproduce the various dynamical signatures of cortical networks (Table 1). Therefore, we propose that the role of multiple mechanisms of the E–I homeostasis does extend beyond the local circuit level (Gainey & Feldman, 2017; Wen & Turrigiano, 2024), shaping the collective dynamics of large-scale cortical networks.

### Effects of the E–I Homeostasis on Collective Dynamics Depend on Fast Local Rhythms

The version of the Wilson–Cowan implemented to simulate the local dynamics of cortical areas is based on the fast dynamics of gamma oscillations (∼40Hz). These rhythms are generated by the recurrent loop between E–I populations, with fast inhibition provided by parvalbumin-positive (PV) interneurons and fast excitation through *α*-amino-3-hydroxy-5-methyl-4-isoxazolepropionic (AMPA) receptors (Buzsáki & Wang, 2012; Wang, 2010; Wang & Buzsáki, 1996). While the regulation of AMPA receptors has an important participation in the homeostasis of excitatory synapses (Turrigiano, 2011), experimental results show that the slower NMDA receptors are also modulated to maintain the homeostasis of firing rates (Watt, et al., 2000). Therefore, it is relevant to investigate if the effects of the local E–I homeostasis on the emergence of FC, FCD, and collective metastable dynamics are limited to its impact on fast gamma rhythms, related to AMPA receptors, or can also be observed when accounting for the slower NMDA dynamics.

**Figure 5.**
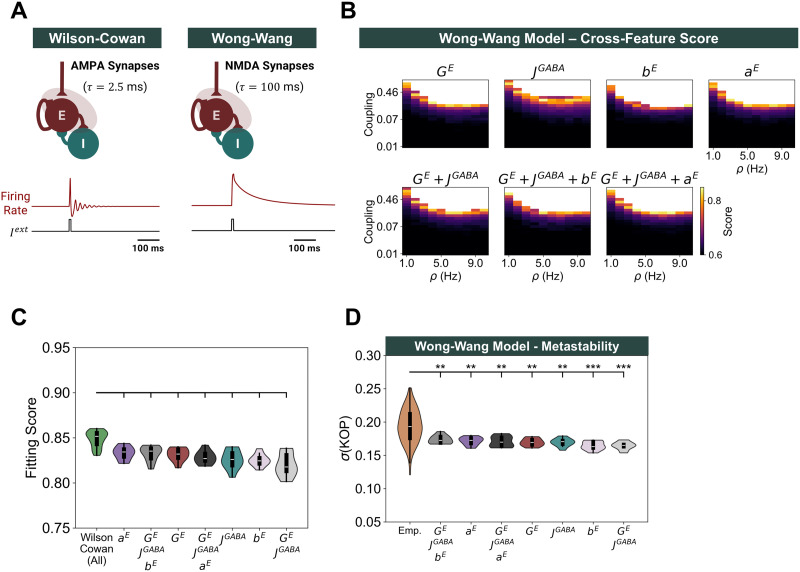
Effects of the E–I homeostasis in models with slow local dynamics. (A) Response of the Wilson–Cowan (left) and Wong–Wang (right) models to perturbations in external inputs. While the Wilson–Cowan model quickly returns to the equilibrium, exhibiting transient gamma oscillations, the Wong–Wang model takes longer to recover due to the slower dynamics of NMDA synapses. (B) Parameter spaces representing the cross-feature score of models based on the Wong–Wang dynamics for different combinations of *C* and *ρ*. Blank spaces represent combinations of *C* and *ρ* for which the homeostatic set point is not valid (i.e., mean firing rates differ from *ρ* by more than 15% in at least one cortical area). (C) Comparison between cross-feature fitting scores at the optimal point of each mechanism of homeostasis and the Wilson–Cowan model with homeostasis of *G^E^, c^EI^, μ^E^*, and *σ^E^*. Distributions correspond to the result of 10 simulations with optimal *C* and *ρ*. Brackets indicate a significant difference, with *p* < 0.05 from a Mann–Whitney *U* test. All *p* values were FDR corrected. (D) Comparison between metastability in empirical data and models at the optimal point (best cross-feature fitting score) for each mechanism of homeostasis. Values correspond to the results of 10 simulations with optimal *C* and *ρ*. Brackets represent a significant difference between models and empirical data from a Mann–Whitney *U* test (*p < 0.05; **p < 0.01; ***p < 0.001). All *p* values were FDR corrected.

To investigate this, we implemented a large-scale model with local dynamics based on the reduced Wong–Wang model (Deco et al., 2014), where excitatory synapses have slower time constants based on the timescales of NMDA receptors (Figure 5A). First, following the framework of Páscoa dos Santos and Verschure (2025), previously implemented for the Wilson–Cowan model, we derive the analytical expressions to calculate the parameter values at the homeostatic fixed point for each empirically derived mechanism of homeostasis. For more details on the derivation, refer to the SI Methods and Supporting Information Figure S27. Importantly, since the timescale of excitatory synapses (100 ms) is substantially slower than the timescale of inhibition (10 ms), the Wong–Wang model does not have a Hopf bifurcation. Therefore, the local dynamics dependent on NMDA receptors remain in the stable regime for all tested combinations of external input and target firing rate, regardless of the combination of homeostatic mechanisms (Supporting Information Figure S28), as opposed to the strong bifurcation modulation observed for the Wilson–Cowan model (Figures 1B and Supporting Information Figure S1).

With that in mind, we explore the performance of large-scale models based on Wong–Wang local dynamics by studying the dynamics of networks with different combinations of target firing rate (*ρ*) and global coupling (*C*). In line with the implementation of Deco et al. (2014), we do not account for interareal conduction delays, due to their negligible impact on the slow NMDA dynamics. Furthermore, following Deco et al. (2014), we consider a simulation valid if the firing rate is within 15% of the target, as opposed to the more stringent condition of 1% imposed on the Wilson–Cowan model. For each combination of parameters, we analyze how the model reproduces empirical FC and FCD by computing the cross-fitting scores. Our results indicate that the score depends on the interaction between *C* and *ρ*, similarly to the Wilson–Cowan model (Figure 5B). The optimal values of *C* and *ρ* and the respective scores can be consulted in Supporting Information Table S1. When comparing results at the optimal point for each model, our results show that there is no significant difference between the fitting scores of models with distinct mechanisms of homeostasis (Figure 5C). This is due to the reduced impact of the E–I homeostasis on the local NDMA dynamics (Supporting Information Figure S28), when compared with its effect on the dynamics of faster gamma rhythms, dependent on AMPA receptors (Figures 1B and Supporting Information S1; Páscoa dos Santos & Verschure, 2025). More importantly, the performance of any of the networks based on the Wong–Wang model is significantly lower than the performance of the Wilson–Cowan model with the homeostasis of excitation (*G^E^*), inhibition (*c^EI^*) and excitability slope (*σ^E^*), and threshold (*μ^E^*; Figure 5C). In line with this result, even though we also found a strong relationship between metastability and the emergence of FC and FCD when using the Wong–Wang model (Supporting Information Figure S29), networks based on slow NMDA dynamics were not able to reach the levels of metastability observed in empirical data (Figure 5D).

That considered, our results suggest that the resting-state collective dynamics of the cortex, measured with BOLD fMRI, more strongly reflect the interaction between gamma rhythms generated by fast excitation and inhibition than the slower dynamics of NDMA receptors. Therefore, given the strong impact of the E–I homeostasis on local gamma dynamics, the collective dynamics of the cortex at rest are strongly dependent on the mechanisms employed for the homeostasis of firing rates.

### Flexible Network Dynamics Emerge Independently of Noise

**Figure 6.**
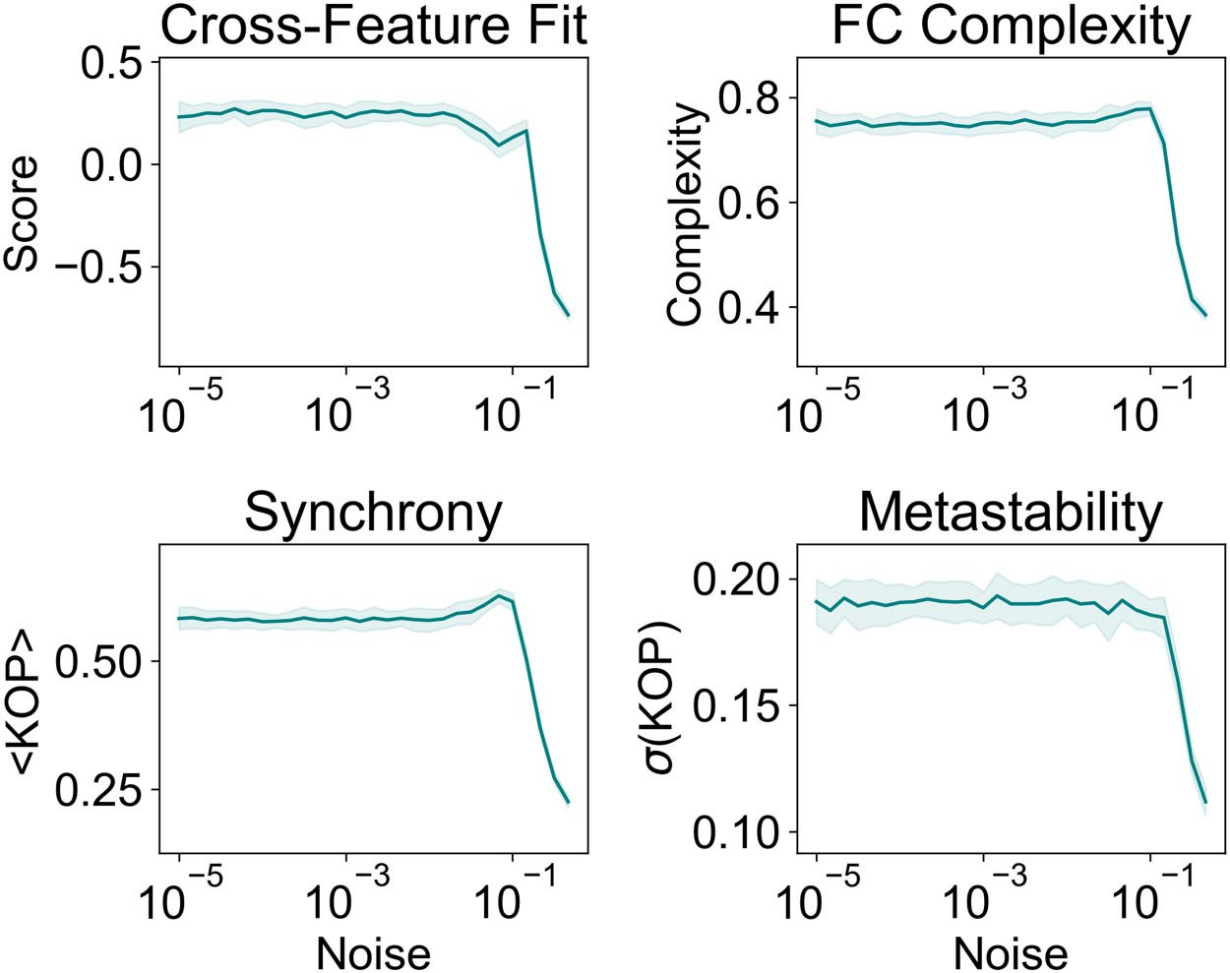
Changes in network features with the variance of noise. Values relate to models with *C* = 3.59 and *ρ* = 0.12 and all mean delays between 0 and 40 ms corresponding to valid network solutions. Solid lines represent the average, and shaded areas represent the standard deviation across mean delays.

Synaptic transmission is known that one to be noisy in brain networks (Rusakov, Savtchenko, & Latham, 2020). This variability can have a relevant role in spontaneous brain dynamics (Pinneo, 1966; Rusakov et al., 2020), underlying phenomena such as stochastic resonance (Deco, Jirsa, & McIntosh, 2011; Deco, Jirsa, McIntosh, Sporns, & Kötter, 2009; Schirner et al., 2022). In addition, systems with multiple weakly stable attractor states display noise-driven transitions between attractors, corresponding to multistable dynamics (Deco & Jirsa, 2012; Schirner et al., 2022), which can be confounded with metastability (Kelso, 2012). In metastable systems, collective dynamics emerge as a consequence of the interaction between network topology and node dynamics, largely independently of noise. However, it is not yet clear if resting-state brain activity is more reflective of a metastable or multistable system (Kelso, 2012; Schirner et al., 2022). For that reason, we explore the behavior of our network under different levels of noise (see the Methods section). Given that the model with all mechanisms of homeostasis better reproduces empirical dynamics, connectivity, and complexity, we take the optimal model with all forms of homeostasis (*C* = 3.59, *ρ* = 0.12) and simulate BOLD signals under varying levels of noise (see the Methods section). We then analyze model performance, collective dynamics, and FC complexity to assess how they are influenced by the variability of neuronal noise. Since we did not observe an interaction between noise and conduction delays (Supporting Information Figure S30), we present the average results across mean delays (Figure 6). Our results demonstrate that there is no significant effect of noise on model performance, collective dynamics, and FC complexity for lower noise levels. Conversely, as the variance of noise increases beyond these levels, model performance deteriorates. This effect is to be expected, given that the magnitude of noise becomes comparable with the intrinsic fluctuations in node dynamics (node activity is constrained between 0 and 1 by the sigmoid activation function). Furthermore, we observe a small peak in synchrony at levels of noise close to 0.1, which could suggest stochastic resonance (Deco et al., 2009), in line with studies showing noise-induced synchronization due to the structure of the human connectome (Pang, Gollo, & Roberts, 2021). However, given that this peak does not correspond to the emergence of empirically relevant dynamics and there are no significant effects on the remaining network features, we do not explore this further. That said, given that model performance and dynamics are largely independent of noise, our results favor the emergence of metastable dynamics, as opposed to multistability (Kelso, 2012; Schirner et al., 2022). For this reason, we consider that the collective dynamics of our models are most likely a product of the emergence of metastability, shaped by edge-of-bifurcation dynamics at the circuit level (regulated by E–I homeostasis) and the topology of interareal interactions (constrained by the structural connectome).

### Multiple Homeostatic Mechanisms Drive the Reemergence of Functional Networks and Collective Dynamics After Structural Lesions

Our results establish the key role of local E–I balance and its homeostasis for the emergence of empirically valid large-scale networks and dynamics in cortical networks. E–I homeostasis can also be a significant driver of the reemergence of large-scale FC properties in the lesioned cortex (Chakraborty et al., 2023; Páscoa dos Santos & Verschure, 2022; Páscoa dos Santos et al., 2023; Vattikonda et al., 2016). However, our earlier results (Páscoa dos Santos et al., 2023) suggest that scaling of inhibition, the homeostatic mechanism most commonly implemented in large-scale models (Abeysuriya et al., 2018; Castaldo et al., 2023; Deco et al., 2014; Hellyer et al., 2016; Naskar et al., 2021), is insufficient by itself for the recovery of metastable dynamics. Therefore, we investigate if the conjugation of multiple forms of homeostasis identified here can potentiate the reemergence of not only FC but also network dynamics following structural lesions. To do this, we apply structural lesions in the optimal model: including homeostasis of excitation (*G^E^*), inhibition (*c^EI^*), and intrinsic excitability (*μ^E^* and *σ^E^*) with *C* = 3.59, *ρ* = 0.12, and mean delay = 40 ms, corresponding to a conduction velocity (3.9 m/s) close to the values reported in previous studies (Lemaréchal et al., 2022; Petkoski & Jirsa, 2022). We extract activity before the lesion (healthy), after the lesion (acute), and after node parameters are adapted to restore local E–I balance (chronic). For more details on the simulation protocol and analysis (Páscoa dos Santos et al., 2023), refer to the Methods section and SI Methods. All *p* values presented in this section relate to the result of the Wilcoxon rank-sum test and are FDR corrected.

**Figure 7.**
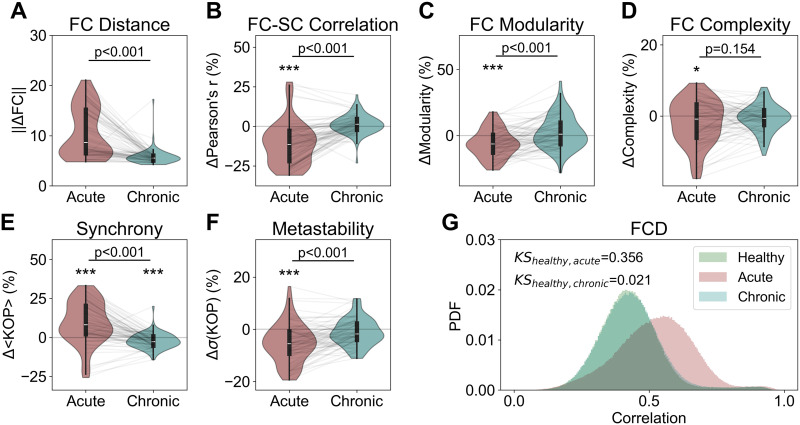
Disruption and recovery of network properties after focal lesion in models with homeostasis of excitation (*G^E^*), inhibition (*c^EI^*), and intrinsic excitability (*μ^E^* + *σ^E^*). (A) Distance from baseline of FC matrices in acute (postlesion) and chronic (postlesion with homeostatic recovery of E–I balance) simulations. (B) Change from baseline (in percentage) of FC-SC correlation in the acute and chronic simulations. (C) Same as B, for FC modularity. (D) Same as B, for FC complexity. (E) Same as B, for synchrony. (F) Same as B, for metastability. (G) Distributions of FCD correlations in all stages of our lesion simulations. In addition, we present the Kolmogorov–Smirnov distance between distributions at baseline and acute/chronic simulations. In all plots, *p* values show the significance of the Wilcoxon rank-sum test (*p < 0.05; **p < 0.01; ***p < 0.001). All *p* values were FDR corrected.

Starting with FC, we measure the Euclidean distance between healthy FC and FC in the acute and chronic periods (Figure 7A). We observed that, even though FC patterns are disrupted in the acute period (10.751 ± 4.900), significant recovery toward prelesion FC configurations (5.800 ± 1.573; *p* < 0.001) occurs in the chronic period. Consistent with stroke literature (Zhang et al., 2017), the model displays an acute decrease in their correlation between structural and functional networks (−10.97 ± 13.34%; *p* < 0.001; Figure 7B). However, this disruption was recovered in the chronic period through the action of the E–I homeostasis (1.33 ± 6.10%; *p* = 0.059). In addition, we observe an acute reduction of modularity (−6.00 ± 10.39%; *p* < 0.001; Figure 7C), consistent with that observed in stroke patients (Gratton, Nomura, Pérez, & D’Esposito, 2012; Siegel et al., 2018). In line with previous results (Páscoa dos Santos et al., 2023; Siegel et al., 2018), the recovery of the E–I balance drives the reemergence of modularity toward prelesion levels in the chronic period (2.41 ± 12.44%; *p* = 0.257). Therefore, our model can replicate the effects of lesions on FC and its subsequent recovery through the focal E–I homeostasis. We further explore the effects of lesions on functional complexity (Figure 7D), revealing a minimal decrease in the acute period (−1.86 ± 6.21%; *p* = 0.046) and a return to baseline after recovery of the E–I balance (−0.62 ± 3.81%; *p* = 0.240). Therefore, our results suggest that, as opposed to the FC-SC relation and FC modularity, FC complexity may be minimally disrupted by lesioning the connectome.

Beyond reproducing the effects of stroke, and subsequent recovery, on FC (Gratton et al., 2012; Páscoa dos Santos et al., 2023; Siegel et al., 2016; Vattikonda et al., 2016; Zhang et al., 2017), we explore how lesions disrupt network dynamics. Starting with synchrony, our results indicate that the system tends toward hypersynchrony in the acute period (9.31 ± 13.44%; *p* < 0.001; Figure 7E), in line with literature on the effects of brain injury (Hillary & Grafman, 2017). More importantly, synchrony is recovered toward prelesion levels in the chronic period (*p* < 0.001), although a small, but significant, difference from baseline remains (−2.19 ± 4.88%; *p* < 0.001). Regarding metastability, we observe a significant decrease postlesion (−5.30 ± 7.32%; *p* < 0.01; Figure 7F), followed by a significant recovery (*p* < 0.001) to prelesion levels in the chronic period (−1.08 ± 5.17%; *p* = 0.060). Finally, given the relationship between metastability and FCD (Deco et al., 2017; Schirner et al., 2022), we analyze FCD distributions in the simulated healthy, acute, and chronic periods. In line with Páscoa dos Santos et al. (2023), we observe an acute disruption, with the mean FCD correlation values shifting from 0.429 in the healthy period to 0.522 postlesion (KS distance = 0.356; Figure 7F). Importantly, the combination of multiple forms of homeostasis can drive the recovery of FCD distributions to prelesion levels (mean correlation = 0.434; KS distance = 0.021), as opposed to the plasticity of inhibition alone, as attested by our previous results (Páscoa dos Santos et al., 2023).

Finally, we have also performed the same analysis steps in models with the *G^E^* or *G^E^* +*c^EI^* homeostasis, showing that such models are either unable to reproduce the studied poststroke disruptions in FC and network dynamics, or such disruptions were not recovered through the E–I homeostasis (Supporting Information Figures S31–S32).

In summary, the conjugation of multiple mechanisms of homeostasis not only supports metastable dynamics and empirical FC networks but can also drive their reemergence following lesions to the connectome, which is not the case in models with homeostasis of excitation or inhibition only. Therefore, the multifactor homeostasis of E–I balance can also be considered a robust means of self-organization in large-scale cortical networks.

## DISCUSSION

Building on the observation that multiple mechanisms of the E–I homeostasis are essential for the regulation of local circuit dynamics (Wen & Turrigiano, 2024), we extend this hypothesis to large-scale networks, exploring how distinct homeostatic mechanisms impact the global dynamics and functional networks of the human cortex. Our results demonstrate that the homeostatic regulation of local E–I balance supports the emergence of resting-state FC and FCD, particularly when involving forms of homeostasis that modulate the distance to the bifurcation between damped and sustained oscillations (Páscoa dos Santos & Verschure, 2025; Figure 1B and Supporting Information Figure S1). In addition, we show that the resulting heterogeneity in the distance to the bifurcation across cortical areas supports the emergence of empirical FC and FCD by compensating for heterogeneities in the magnitude of input fluctuations. We further demonstrate that the optimal working points of models generally correspond to moderate levels of synchrony and heightened metastability, confirming that cortical networks might operate in a regime of enhanced metastability. More importantly, empirical levels of synchrony and metastability can only be replicated by models that incorporate multiple mechanisms of homeostasis. Furthermore, we study the complexity of large-scale functional interactions, stressing that it depends not only on the topology of structural networks (Arsiwalla & Verschure, 2016; Sporns et al., 2002; Zamora-López et al., 2016) but also on local E–I balance. Importantly, empirical levels of complexity were more closely approximated by the model with all forms of homeostasis enabled. Altogether, we confirm the hypothesis that the benefits of the interaction between multiple forms of the E–I homeostasis extend beyond the local circuit level (Gainey & Feldman, 2017; Maffei et al., 2004; Maffei & Turrigiano, 2008; Turrigiano, 2011; Wen & Turrigiano, 2024), contributing to the regulation of large-scale dynamics toward a regime of maximal metastability and complexity, which underlies the FC networks observed in the resting-state cortex. Finally, we also demonstrate that the combined action of multiple homeostatic mechanisms can drive the reemergence of not only FC but also network dynamics following focal lesions to the connectome (Páscoa dos Santos et al., 2023), evidencing the robustness of the E–I homeostasis as a self-organization process in cortical networks. Importantly, our study provides a framework to tie several relevant features of cortical organization and dynamics across spatial scales. More specifically, the regulation of E–I balance in cortical neurons allows for the regulation of edge-of-bifurcation dynamics at the circuit level, which, in turn, underlies the self-organization of spontaneous large-scale dynamics toward a regime of high metastability, underlying the resting-state activity of the human cortex.

To our knowledge, we are the first to explore the combined action of diverse mechanisms of the E–I homeostasis in large-scale models, given that most studies focus on the homeostasis of inhibition (Abeysuriya et al., 2018; Castaldo et al., 2023; Deco et al., 2014; Hellyer et al., 2016; Naskar et al., 2021; Páscoa dos Santos et al., 2023). At the circuit level, the synergy between the synaptic scaling of excitation and inhibition and the plasticity of intrinsic excitability are essential for the maintenance of not only stable firing rates but also edge-of-bifurcation dynamics (i.e., criticality; Gainey & Feldman, 2017; Rutherford, Nelson, & Turrigiano, 1998; Wen & Turrigiano, 2024). Our results add to this perspective by suggesting that these benefits extend to larger scales, supporting network dynamics and FC networks. More specifically, our results show that networks with synaptic scaling of excitation and inhibition reach heightened levels of global metastability, characteristic of the cortex at rest (Deco et al., 2017; Freeman, 2005; Freeman & Holmes, 2005; Hancock et al., 2023; Schirner et al., 2022). Regarding the plasticity of intrinsic excitability, it is not yet clear from experimental data if it acts through the modulation of firing thresholds only (*μ^E^*; Maffei & Turrigiano, 2008) or the concerted adaptation of both the threshold and slope of neuronal excitability (*μ^E^* +*σ^E^*; Desai et al., 1999; Nataraj et al., 2010; Wen & Turrigiano, 2021). Here, we suggest that the second option is more plausible given its contribution to ensuring the moderate levels of synchrony observed in empirical data, while still allowing for highly metastable dynamics. In this line, it should be highlighted that recent results suggest that the plasticity of intrinsic excitability might not be mobilized in the adult cortex, being instead pivotal during development (Wen & Turrigiano, 2021). However, it has also been suggested that homeostasis of intrinsic excitability can also be mobilized following substantial perturbations that cannot be compensated for by the homeostasis of excitation and inhibition (Turrigiano, 2011). This aligns with evidence from the poststroke brain, where processes typical of the developmental phases, such as neurogenesis, are also recruited for recovery (Cuartero et al., 2021). Therefore, we argue that the diverse mechanisms of homeostasis have specific contributions to cortical dynamics at both the circuit and global scales, which may play a role in both the healthy and diseased brain.

In light of our results, it is relevant to reflect on how exactly local E–I balance contributes to the large-scale dynamics of the cortex. Here, we focus on the edge-of-bifurcation theory for cortical dynamics (Arsiwalla & Verschure, 2017; Cabral et al., 2014; Deco & Jirsa, 2012; Deco et al., 2017; Freeman, 2005; Freeman & Holmes, 2005; Schirner et al., 2022). Under this perspective, local circuits at the edge-of-bifurcation can undergo spontaneous phase transitions, which, when coincident across cortical areas, can self-reinforce, leading to the emergence of the collective behaviors typical of the cortex (Freeman, 2005). In our model, these collective dynamics are strongly regulated by the maintenance of stable mean firing rates (*ρ*) through the E–I homeostasis (Páscoa dos Santos & Verschure, 2025). For example, increases in global coupling (*C*), which may push node dynamics beyond the bifurcation, can be compensated by lowering *ρ*. For this reason, model performance and dynamics are heavily dependent on the interaction between *C* and *ρ*. On the other hand, the heterogeneity in the in-degrees of the connectome leads not only to stronger inputs in highly connected nodes but also to higher input fluctuations (Supporting Information Figure S17). Therefore, if all cortical areas were to be equally distant from the bifurcation, the highly connected ones would be more likely to engage in sustained oscillations due to strong input fluctuations that push them beyond the bifurcation. However, this effect can be compensated for by forms of homeostasis (i.e., excitation and excitability slopes) that maintain stable firing rates by scaling the distance to the bifurcation (Figure 1B and Supporting Information Figure S1; Páscoa dos Santos & Verschure, 2025), poising highly connected nodes farther away from the bifurcation point. This adjustment is critical to ensure that cortical areas exhibit edge-of-bifurcation dynamics regardless of the variability of their inputs. More so, this aligns with previous studies showing that distance to criticality varies across cortical areas (Harris, Gollo, & Fulcher, 2024) and that global metastable dynamics are better reproduced in models with heterogeneous distances to the bifurcation (Deco et al., 2017). More so, recent computational studies investigating brain dynamics during different states of consciousness are in line with our findings that strongly connected areas are poised further away from the bifurcation, at least in the awake cortex (López-González et al., 2021). Importantly, we demonstrate that this heterogeneity, related to differences in connectivity, can emerge naturally from the maintenance of stable population firing rates via multiple forms of homeostasis. In this line, we suggest that future studies investigate how the heterogeneity in distance-to-bifurcation across cortical areas (Deco et al., 2017; Harris et al., 2024; López-González et al., 2021) relates to their structural connectivity and microcircuitry (Wang, 2020) or to the interaction between E–I homeostasis and states of reduced consciousness (López-González et al., 2021).

Our results indicate that empirical FCD emerge in a state of maximal metastability (Supporting Information Figure S23), confirming that FCD reflects the exploration of the state space of network configurations potentiated by metastable brain dynamics (Deco et al., 2017; Freeman, 2005; Hancock et al., 2023; Schirner et al., 2022; Tognoli and Kelso, 2014). Furthermore, it has been argued that metastability is generally derived from abstract Hopf bifurcation models, with no direct relationship to physiological processes (Schirner et al., 2022). However, we point out that previous studies, although not quantifying metastability directly, have demonstrated the emergence of large-scale wave patterns consistent with metastable dynamics in models similar to ours (Roberts et al., 2019). Here, we demonstrate that these theoretical concepts are directly applicable in a model of the neocortex where the generation of oscillations is based on E–I interactions and bifurcation control implemented by the E–I homeostasis. This confirms that the concept of metastability is compatible with both the architecture of the human cortex and its processes of self-organization. Importantly, our results also show that the collective dynamics of the human cortex most likely reflect metastability and not multistability (Kelso, 2012), making a further argument in favor of the hypothesis that metastability is one of the key dynamical features of large-scale cortical networks (Freeman & Holmes, 2005; Hancock et al., 2023; Schirner et al., 2022; Tognoli & Kelso, 2014).

Our analysis further shows a relationship between metastable dynamics and the complexity of functional interactions. In complex systems, the interactions between units are dictated by both the structural scaffold and circuit dynamics (Hens, Harush, Haber, Cohen, & Barzel, 2019). In the cortex, the architecture of the connectome plays an important role in the emergence of complex spontaneous activity (Arsiwalla & Verschure, 2016; Sporns et al., 2002) and its graph properties are required to support the complexity observed in empirical FC networks (Zamora-López et al., 2016). By exploring the relationship between complexity and metastability, we demonstrate that FC complexity not only reflects the topology of the connectome but is also tightly related to metastable dynamics (Figure 4B and Supporting Information Figure S23), suggesting it reflects a balanced exploration of network states (Hancock et al., 2023; Tognoli & Kelso, 2014). When averaged over long time scales, this exploration leads to a distribution of FC weights that is closer to uniformity, revealing a dynamic balance between integration and segregation. For this reason, we propose that the complexity of functional interactions can be another signature of local E–I balance, which allows for the emergence of metastable collective dynamics (Cabral et al., 2014; Deco et al., 2017; Freeman, 2005; Freeman & Holmes, 2005) through the regulation of local edge-of-bifurcation dynamics (Páscoa dos Santos & Verschure, 2025). That said, networks with homeostasis of excitation (*G^E^*) or excitation and inhibition (*G^E^* +*c^EI^*), associated with high metastability (Figure 3B), display levels of complexity considerably lower than found in empirical data (Figure 4B). However, such models also show overly synchronized dynamics (Figure 3A). Conversely, when the plasticity of intrinsic excitability (*μ^E^* +*σ^E^*) is introduced, synchrony is maintained at empirical levels and complexity enhanced. Therefore, we propose that the plasticity of intrinsic excitability has an important role in maintaining complex interactions by avoiding hypersynchrony while still allowing for highly metastable dynamics.

We demonstrate not only the association between local E–I balance and collective metastable dynamics but also the relevant role of gamma oscillations as a connecting mechanism between the local and global scales. To do this, we compare the dynamics in models with local circuits based on either fast AMPA (i.e., Wilson–Cowan) or slow NMDA (i.e., Wong–Wang) synapses, both equipped with the local E–I homeostasis. The first relevant conclusion from this exploration is that, in models with slow excitation, as long as mean firing rates are maintained locally, collective dynamics are not heavily shaped by which homeostatic mechanisms are in place (Figure 5C). We suggest that this occurs because the Wong–Wang model is not capable of generating oscillations and, thus, the bifurcation modulation of some homeostatic mechanisms, discussed in Páscoa dos Santos and Verschure (2025), is not observed in the Wong–Wang model (Supporting Information Figure S28). Conversely, in the Wilson–Cowan model, this effect is essential to compensate for heterogeneities in input fluctuations and, therefore, the combination of local homeostatic mechanisms strongly impact global dynamics. For this reason, the way multiple homeostatic mechanisms shape collective dynamics is strongly tied to the regulation of edge-of-bifurcation oscillations and not just the maintenance of firing rates. More importantly, our results also show that models with slow excitation cannot reach the metastability levels observed in empirical resting-state data (Figure 5D). Here, we propose that the relevance of gamma rhythms for the collective dynamics of our model has two main explanations. The first, and more obvious, is that we base our measurements on the dynamics of BOLD signals, which have been associated with fluctuations in gamma power and synchrony in both experimental (Logothetis, Pauls, Augath, Trinath, & Oeltermann, 2001; Niessing et al., 2005; Schölvinck, Maier, Ye, Duyn, & Leopold, 2010) and computational (Cabral, Hugues, Sporns, & Deco, 2011; Castaldo et al., 2023; Deco et al., 2009) studies. Therefore, it is natural that models that generate gamma rhythms would be more equipped to reproduce the metastable dynamics of BOLD signals. However, based on previous theories for the generation of spontaneous cortical dynamics (Freeman, 2005; Freeman & Holmes, 2005; Turkheimer et al., 2015, 2022), we advance a further explanation. At the local level, E–I balance is maintained by regulating not only excitation but also the fast GABAergic synapses from fast-spiking interneurons (Maffei, Nataraj, Nelson, & Turrigiano, 2006; Turrigiano, 2011; Wen & Turrigiano, 2024; Xue et al., 2014). Since gamma oscillations are also generated by the recurrent loop between PY neurons and fast-spiking interneurons (Buzsáki & Wang, 2012; Campanac et al., 2013; Tiesinga & Sejnowski, 2009; Wang, 2010), the E–I homeostasis should have strong effects on gamma oscillations. Previously, we have proposed that one of such effects is the modulation of edge-of-bifurcation oscillations (Páscoa dos Santos & Verschure, 2025), in line with empirical studies showing the role of multiple homeostatic mechanisms in the regulation of both firing rates and circuit-level criticality (Wen & Turrigiano, 2024). That said, given that edge-of-bifurcation oscillations have been shown to support the emergence of metastable activity patterns in the cortex (Deco et al., 2017; Freeman, 2005; Freeman & Holmes, 2005; Roberts et al., 2019), our results support the idea that gamma oscillations can be a necessary binding factor between local E–I balance and global metastable dynamics (Freeman, 2005; Turkheimer et al., 2015). For this reason, we suggest that gamma rhythms should necessarily be accounted for in large-scale models built to study the relationship between local and global dynamics in cortical networks. That said, since the combination of multiple homeostatic mechanisms is pivotal to ensure that gamma frequencies remain within physiological bounds (Páscoa dos Santos & Verschure, 2025), we suggest that large-scale modeling studies should also go beyond the plasticity of inhibition as the sole mechanism of homeostasis when studying E–I balance.

To further validate our model, we also examine the role of the E–I homeostasis in the self-organization of the cortex following focal lesions, following the framework of Páscoa dos Santos et al. (2023). We replicate disruptions in FC properties such as SC-FC coupling (Zhang et al., 2017) and modularity (Siegel et al., 2018), characteristic of the poststroke brain, which are then recovered through cortical reorganization, driven by standard mechanisms of homeostatic plasticity, in line with previous results (Páscoa dos Santos et al., 2023). However, the model in Páscoa dos Santos et al. (2023) was equipped with the plasticity of inhibition only, which proved insufficient for the recovery of metastable dynamics and FCD. Here, we demonstrate that networks with multiple mechanisms of the E–I homeostasis (excitatory and inhibitory synapses and intrinsic excitability) can not only replicate the disruptions and subsequent recovery of macroscale FC properties but also return metastability and FCD to prelesion levels through the restoration of E–I balance (Figure 6). That said, not all mechanisms of homeostasis may be necessary for the recovery of network dynamics. Therefore, we apply the same lesion protocol in networks with either *G^E^* or *G^E^* +*c^EI^* homeostasis (Supporting Information Figures S31–S32). Critically, those are either unable to recover network dynamics or do not reproduce empirical poststroke deficits in FC (Gratton et al., 2012; Siegel et al., 2018; Zhang et al., 2017). Therefore, while the homeostasis of inhibition might strongly contribute to the recovery of FC networks, as demonstrated in Páscoa dos Santos et al. (2023), the plasticity of excitation and intrinsic excitability is critical for ensuring that network dynamics can be recovered following damage to structural networks.

Our results show that, by imposing stronger constraints on the local circuitry of cortical networks, the E–I homeostasis potentiates the flexible exploration of macroscale network states. While this effect appears paradoxical, it is in line with the concept of “constraints that deconstrain” (Doyle & Csete, 2011). This idea stems from the work of Kirschner and Gerhart (1998), who suggest that some constraining mechanisms are preserved through evolution because they deconstrain other processes that are advantageous for the organisms. In neuroscience, this concept has been applied to explain how the layered architecture of brain networks (Verschure, 2012; Verschure, Kröse, & Pfeifer, 1992) ensures both robust motor control and high adaptability in behavior (Doyle & Csete, 2011). We propose that the tight control of E–I balance by multiple mechanisms of homeostasis is another example of a “constraint that deconstrains,” by supporting the emergence of metastability that endows the cortex with the flexibility needed to explore its state space during rest (Freeman, 2005; Freeman & Holmes, 2005; Hancock et al., 2023; Raichle, 2011, 2015; Tognoli & Kelso, 2014).

There are, however, some limitations to point out in our model. The first is that we do not explore homeostatic plasticity at the level of inhibitory neurons. Recent studies have shown that the plasticity of excitatory connections (Ma et al., 2019) or intrinsic excitability (Gainey & Feldman, 2017) in PV interneurons can also contribute to the E–I homeostasis. Therefore, future models should consider the implementation of the E–I homeostasis in inhibitory neurons, delving into the interaction between the homeostatic set points of excitatory and inhibitory populations. Another limitation relates to the fact that, while synaptic scaling of excitation is ubiquitous across cortical layers (Hengen et al., 2013; Keck et al., 2013; Maffei et al., 2004; Maffei & Turrigiano, 2008; Wen & Turrigiano, 2021), other forms of plasticity, such as the scaling of inhibition, might be absent from deeper layers (Barnes et al., 2015; Hengen et al., 2013; Maffei et al., 2004; Maffei & Turrigiano, 2008; Wen & Turrigiano, 2021). Therefore, we suggest that the E–I homeostasis should be explored in large-scale models accounting for the laminar structure of the neocortex (Mejias, Murray, Kennedy, & Wang, 2016; Sanchez-Todo et al., 2023). Furthermore, we implement a homogeneous target firing rate across the network, in line with previous modeling studies (Abeysuriya et al., 2018; Deco et al., 2014; Hellyer et al., 2016; Naskar et al., 2021). While *in vivo* studies of the E–I homeostasis are mostly based on the activity of visual or somatosensory cortices (Hengen et al., 2013; Ma et al., 2019), different cortical areas might be regulated toward area-specific set points, possibly following hierarchical gradients of cortical organization (Wang, 2020). For this reason, future models should explore the possibility of implementing not only heterogeneous target firing rates but also the interaction between cortical hierarchy, structural heterogeneities (Kong et al., 2021), and the homeostasis of E–I balance. In addition, our model can also be used in conjunction with a more data-driven approach, as employed by Kong et al. (2021), to optimize model parameters, investigating how this interacts with the local homeostasis of E–I balance.

Finally, we tune the Wilson–Cowan model to generate gamma oscillations, which have been related to fluctuations in BOLD signals (Cabral et al., 2014; Castaldo et al., 2023; Deco et al., 2009; Schölvinck et al., 2010) and transient rhythms across frequency bands (Cabral et al., 2022). While there is empirical evidence for gamma resonance through the recurrent interactions between PY and PV neurons (Buzsáki & Wang, 2012; Wang & Buzsáki, 1996), some studies suggest that cortical networks also generate alpha oscillations in deeper layers (Maier, Adams, Aura, & Leopold, 2010; van Kerkoerle et al., 2014). Therefore, the extension of our framework to the laminar architecture of the cortex (Mejias et al., 2016; Sanchez-Todo et al., 2023) might also provide new insight into how E–I balance interacts with different cortical rhythms, fast and slow inhibition (Páscoa dos Santos & Verschure, 2025), and how it shapes cross-frequency interactions.

To conclude, our results demonstrate the critical role of the firing-rate homeostasis, which regulates edge-of-bifurcation dynamics at the circuit level, as a key piece underlying the self-organization of resting-state cortical activity toward a regime of heightened metastability where resting-state networks emerge. More importantly, we show that this reflects the joint action of multiple homeostatic mechanisms employed to maintain E–I balance, suggesting that their influence extends beyond the neuronal and circuit level, underlying the rich spontaneous dynamics of cortical networks.

## Acknowledgment

F.P.S. is supported by the European Commission through the euSNN project (Erasmus+ MSCA-ITN ETN H2020dID 860563). P.F.M.J.V. is supported by AISN (Horizon Europe, 101057655), EBRAINS-HEALTH (Horizon Europe, 101058516), PHRASE (European Innovation Council, 101058240), the euSNN project (Erasmus+ MSCA-ITN ETN H2020 860563), and and ReHyb (Horizon2020 871767) CAVAA (EIC Pathfinder 101071178).

## Supporting Information

Supporting information for this article is available at https://doi.org/10.1162/netn_a_00460.

## Author Contributions

Francisco Páscoa dos Santos: Conceptualization; Formal analysis; Methodology; Software; Visualization; Writing – original draft. Paul F. M. J. Verschure: Funding acquisition; Supervision; Writing – review & editing.

## Funding Information

Francisco Páscoa dos Santos, Horizon 2020 (https://dx.doi.org/10.13039/501100007601), Award ID: 860563. Paul FMJ Verschure, HORIZON EUROPE Framework Programme (https://dx.doi.org/10.13039/100018693), Award ID: 101057655. Paul FMJ Verschure, HORIZON EUROPE Framework Programme (https://dx.doi.org/10.13039/100018693), Award ID: 101058516. Paul FMJ Verschure, HORIZON EUROPE European Innovation Council (https://dx.doi.org/10.13039/100018703), Award ID: 101058240. Paul FMJ Verschure, Horizon 2020 (https://dx.doi.org/10.13039/501100007601), Award ID: 871767. Paul FMJ Verschure, Horizon 2020 (https://dx.doi.org/10.13039/501100007601), Award ID: 860563. Paul FMJ Verschure, EIC Pathfinder, Award ID: 101071178.

## Data Availability Statement

All the code necessary to run simulations, together with structural connectivity data, is provided in https://gitlab.com/francpsantos/wc_network_homeostasis. Data from individual subjects can be provided upon request.

## Competing Interests

F.P.S. was employed by Eodyne Systems SL. P.F.M.J.V. is the founder and shareholder of Eodyne Systems S.L., which brings scientifically validated neurorehabilitation and education technologies to society.

## Supplementary Material



## TECHNICAL TERMS

Complex systems: Systems comprising multiple interacting units with collective behaviors that are qualitatively different from the behavior of the units.
Metastability: Property of systems that can move flexibly and continuously between quasistable states—in this case, synchrony and asynchrony.
Emergence: Phenomenon whereby the collective behavior of a complex system is distinct from the summed behavior of its parts.
Connectome: Network of structural connections between distant parts of the cerebral cortex through white-matter fibers.
Bifurcation: Point where a system transitions from one type of behavior to another—in this case, stable activity to persistent oscillations.
Excitatory–inhibitory homeostasis: Plasticity mechanisms employed by cortical neurons to maintain stable activity by balancing excitatory and inhibitory inputs.
Functional connectivity: Structure of pairwise correlations between ultra-slow fluctuations in activity of cortical areas.
Self-organization: Ability of complex systems to adapt and self-regulate toward a given regime or behavior without being directed externally.
Gamma-band oscillations: Rhythmic activity of cortical circuits, between ∼30 and 100 Hz, generated by reciprocal interactions between excitatory and inhibitory populations.
