# Supplementary material for: Metastable dynamics emerge from local excitatory–inhibitory homeostasis in the cortex at rest
Source: Netw Neurosci. 2025 Jul 29;9(3):938–68. doi: 10.1162/netn_a_00460 (PMC12543307; doi:10.1162/netn_a_00460)
Supplement: Supplementary file 1 [file netn-9-3-938-s001.pdf]

Supporting Information for

Metastable Dynamics Emerge from Local Excitatory-Inhibitory Homeostasis in the Cortex at Rest

Francisco Páscoa dos Santos and Paul FMJ Verschure

Francisco Páscoa dos Santos.
E-mail: f.pascoadossantos@gmail.com

This PDF file includes:

Supporting text
Figs. S1 to S32
Tables S1 to S3
SI References

Supporting Information Text

Methods.

Computation of Homeostatic Parameters. In (1) we develop the analytical methodology to compute the value of different parameters of the Wilson-Cowan model allowing for the maintenance of stable activity under various mechanisms of E-I homeostasis. While we present the mathematical expressions here, refer to (1) for a detailed derivation and empirical basis for each mechanism of homeostasis. In all equations below, r^E represents the fixed point r^E corresponding to the target activity actively maintained by cortical networks, here represented by the Wilson-Cowan model.

Excitatory Synapses unto Pyramidal Populations.

$$G^E = \frac{1}{c^{EE}r^E + I^{ext}} \left(\frac{c^{EI}}{1 + exp(-(c^{IE}r^E - \mu^I)/\sigma^I)} - \sigma^E log\left(\frac{1-r^E}{r^E}\right) + \mu^E \right) \quad [1]$$

Inhibitory Synapses unto Pyramidal Populations.

$$c^{EI} = \left(\sigma^E log\left(\frac{1-r^E}{r^E}\right) + G^E c^{EE} r^E + G^E I^{ext} - \mu^E \right) \left(1 + exp(-(c^{IE}r^E - \mu^I)/\sigma^I)\right) \quad [2]$$

Intrinsic Excitability of Excitatory Populations. Homeostatic plasticity of intrinsic excitability, which modulates the parameters of the activation function $F^E(x)$, can be implemented in two manners, considering empirical data (1). The first is at the level of the firing threshold of excitatory populations, μ^E:

$$\mu^E = \sigma^E log\left(\frac{1-r^E}{r^E}\right) - \frac{c^{EI}}{1 + exp(-(c^{IE}r^E - \mu^I)/\sigma^I)} + G^E c^{EE} r^E + G^E I^{ext} \quad [3]$$

In the second case, E-I homeostasis modulates both the firing threshold μ^E and the sensitivity of the activation function σ^E in a coordinated manner so that the value of $F^E(0)$, or the baseline firing rate under no external input, is maintained. To do this, we consider that both parameters can be related to each other as $\sigma^E = K\mu^E$, where K is a constant. For more details on the derivation, refer to (1). Here, we use the default parameters to compute K, so that $K = \frac{\sigma^E_{default}}{\mu^E_{default}} = \frac{0.25}{1} = 0.25$. That said, the values of both parameters can be obtained through the following expression:

$$\begin{aligned} \mu^E &= \frac{1}{Klog\left(\frac{1-r^E}{r^E}\right) - 1} \left(\frac{c^{EI}}{1 + exp(-(c^{IE}r^E - \mu^I)/\sigma^I)} - G^E c^{EE} r^E - G^E I^{ext} \right) \\ \sigma^E &= K\mu^E \end{aligned} \quad [4]$$

Synaptic Scaling of Excitatory and Inhibitory Synapses. Considering that plasticity of excitation and inhibition operate under the same timescale, it is possible to estimate the steady-state values of both parameters given the initial values G^E_0 and c^{EI}_0. For a detailed derivation of the expressions and the extension of the framework to the case of different timescales, please consult (1).

$$\begin{aligned} c^{EI} &= \frac{\sigma^E log\left(\frac{1-r^E}{r^E}\right) + (G^E_0 + c^{EI}_0)(c^{EE}r^E + I^{ext}) - \mu^E}{\frac{1}{1+exp(-(c^{IE}r^E - \mu^I)/\sigma^I)} + c^{EE}r^E + I^{ext}} \\ G^E &= G^E_0 - c^{EI} + c^{EI}_0 \end{aligned} \quad [5]$$

Synaptic Scaling of Excitation and Inhibition and Plasticity of Intrinsic Excitability. Following the previous implementations, we implement homeostasis of excitation and inhibition in conjunction with the two forms of homeostasis of intrinsic excitability. First, for plasticity of μ^E, we have:

$$\begin{aligned} \mu^E &= \frac{\sigma^E log\left(\frac{1-r^E}{r^E}\right) - \frac{c^{EI}_0 - \mu^E_0}{1+exp(-(c^{IE}r^E - \mu^I)/\sigma^I)} + (G^E_0 + \mu^E_0)(c^{EE}r^E + I^{ext})}{1 + \frac{1}{1+exp(-(c^{IE}r^E - \mu^I)/\sigma^I)} + c^{EE}r^E + I^{ext}} \\ G^E &= G^E_0 - \mu^E + \mu^E_0 \\ c^{EI} &= c^{EI}_0 + \mu^E - \mu^E_0 \end{aligned} \quad [6]$$

Similarly, for plasticity of μ^E and σ^E, we obtain:

$$\begin{aligned} \mu^E &= \frac{\frac{c^{EI}_0 - \mu^E_0}{1+exp(-(c^{IE}r^E - \mu^I)/\sigma^I)} - (G^E_0 + \mu^E_0)(c^{EE}r^E + I^{ext})}{Klog\left(\frac{1-r^E}{r^E}\right) - 1 - \frac{1}{1+exp(-(c^{IE}r^E - \mu^I)/\sigma^I)} - c^{EE}r^E - I^{ext}} \\ G^E &= G^E_0 - \mu^E + \mu^E_0 \\ c^{EI} &= c^{EI}_0 + \mu^E - \mu^E_0 \end{aligned} \quad [7]$$

Computation of Homeostatic Parameters as a Function of the Target Firing Rate ρ**.** When the fixed point of the Wilson-Cowan model corresponds to a stable fixed point or spiral, the mean activity of the excitatory population is precisely equal to the fixed-point r^E. However, in systems in a limit-cycle regime, the average firing rate differs from r^E_{fixed}. For this reason, in (1), we derive a method to obtain an estimate of the steady-state model parameters as a function of ρ and I^{ext}, based on the following iterative algorithm, with $\epsilon = 10^{-10}$. For more details, refer to (1).

1. Define upper and lower limits for r^E_{fixed} ($r^E_{lower} = 0$ and $r^E_{upper} = 1$).
2. For $r^E_{fixed} = \frac{r^E_{upper} - r^E_{lower}}{2}$, compute the homeostatic parameters of the system under I^{ext} using the equations in the previous sections with $r^E = r^E_{fixed}$.
3. Solve the system numerically for 5 seconds and compute the average firing rate $\langle r^E \rangle$ from the last 2 seconds of activity.
4. If $|\langle r^E \rangle - \rho| < \epsilon$, take r^E_{fixed} as the fixed point corresponding to ρ and save the homeostatic model parameters.
5. Else, if $\langle r^E \rangle > \rho$, restart the procedure from step 1, updating $r^E_{upper} = r^E_{fixed}$. Conversely, if $\langle r^E \rangle < \rho$, restart from step 1 with $r^E_{lower} = r^E_{fixed}$.

Excitatory-Inhibitory Homeostasis in the Wong-Wang Model.

Derivation of Homeostatic Expressions for the Wong-Wang Model. To derive the expressions allowing for the computation of parameters at the homeostatic fixed point we start with the equations for the fixed points of the model, when $\frac{dS^E}{dt} = 0$ and $\frac{dS^I}{dt} = 0$:

$$\begin{aligned} \frac{S^E}{\tau^E} &= (1 - S^E)\gamma r^E \\ \frac{S^E}{\tau^E} &= \gamma r^E - S^E \gamma r^E \\ S^E\left(\frac{1}{\tau^E} + \gamma r^E\right) &= \gamma r^E \\ S^E &= \frac{\gamma r^E \tau^E}{1 + \gamma r^E \tau^E} \end{aligned} \quad [8]$$

and

$$\begin{aligned} \frac{S^I}{\tau^I} &= r^I \\ S^I &= \tau^I r^I \end{aligned} \quad [9]$$

These equations can then be used together with the expressions for I^E, I^I, r^E, and r^I for the mathematical estimation of the parameter values at the fixed point.

Synaptic Scaling of Excitation. Similarly to the Wilson-Cowan model, the excitatory synapses unto the excitatory population can be modulated through the parameter G^E. To compute the value of G^E at the fixed point, as a function of the external input (I^{ext}) and the target firing rate r^E_{fixed} we start by computing the value of S^E_{fixed} can be computed from r^E_{fixed} using expression 8.

Then, to compute the value of S^I_{fixed} we first substitute S^I by $\tau^I r^I$ (Equation 9), obtaining a function that can be solved for I^I to obtain its fixed point value:

$$\begin{aligned} I^I &= W^I I_0 + J_{NMDA} S^E_{fixed} - S^I_{fixed} \\ I^I &= W^I I_0 + J_{NMDA} S^E_{fixed} - \tau^I r^I \\ I^I &= W^I I_0 + J_{NMDA} S^E_{fixed} - \tau^I F^I(I^I) \\ I^I + \tau^I F^I(I^I) &= W^I I_0 + J_{NMDA} S^E_{fixed} \end{aligned} \quad [10]$$

The resulting value of I^I_{fixed} can then be substituted in equation 9 to obtain S^I_{fixed}.

Then, I^E_{fixed} can be calculated by solving the following expression for I^E:

$$r^E_{fixed} = F^E(I^E) \quad [11]$$

Finally, we re-organize the equation for I^E, obtaining the following expression:

$$\begin{aligned} I^E_{fixed} &= G^E W^E I_0 + G^E w_+ J_{NMDA} S^E_{fixed} + G^E I^{ext} - J_{GABA} S^I_{fixed} \\ G^E &= \frac{I^E_{fixed} + J_{GABA} S^I_{fixed}}{W^E I_0 + w_+ J_{NMDA} S^E_{fixed} + I^{ext}} \end{aligned} \quad [12]$$

where the values of S^E_{fixed}, S^I_{fixed}, and I^E_{fixed} can be inserted to calculate the value of G^E.

Synaptic Scaling of Inhibition. Similarly to the synaptic scaling of excitation, the value of J_{GABA} corresponding to the homeostatic fixed point can be estimated from the expression for I^E as follows:

$$J_{GABA} = \frac{G^E(W^E I_0 + w_+ J_{NMDA} S^E_{fixed} + I_e xt) - I^E_{fixed}}{S^I_{fixed}} \quad [13]$$

Plasticity of Intrinsic Excitability of Excitatory Populations. In the Wong-Wang model, the equivalent of the firing threshold of the input-output function is the parameter b^E. The effect of changing b^E on $F^E(x)$ can be visualized in Figure S27.

In this case, we start by obtaining S^E_{fixed} from expression 8 and then solve the following equation for S^I to obtain S^I_{fixed}:

$$\begin{aligned} S^I &= \tau^I r^I \\ S^I &= \tau^I F^I(I^I) \\ S^I &= \tau^I F^I(W^I I_0 + J_{NMDA} S^E_{fixed} - S^I) \end{aligned} \quad [14]$$

which can then be used, together with S^E_{fixed} to compute I^E_{fixed}.

Finally, the homeostatic value of b^E at the fixed point can be obtained by solving the following expression for b^E:

$$\begin{aligned} r^E_{fixed} &= F^E(I^E_{fixed}) \\ r^E_{fixed} &= \frac{a^E I^E_{fixed} - b^E}{1 - \exp(-d^E(a^E I^E_{fixed} - b^E))} \end{aligned} \quad [15]$$

Similarly, the plasticity of excitability slopes can be approximated by modulation of the parameter a^E. In this case, since $F(x)$ is not a sigmoid function (Figure S27) and approaches $a^E x - b^E$ as x increases, the effect described in (2–4), whereby the slope is modulated without changing the output of the function when the input is equal to 0, can be approximated by varying a^E only. Not only that, since $F(0) = \frac{b^E}{1-\exp(d^E b^E)}$, a^E can be changed without affect the value of $F(x)$ at 0. We present a visual demonstration of this effect in Figure S27.

That said, the homeostatic value of a^E can be computed by solving expression 15 for a^E instead of b^E.

Synaptic Scaling of Excitatory and Inhibitory Synapses. Similarly to the procedure applied in (1) for the Wilson-Cowan model, we consider that the homeostasis of excitation and inhibition operate under the same timescale. Therefore, in line with (1), we consider that:

$$J_{GABA} = J_{GABA_0} + G^E_0 - G^E \quad [16]$$

where J_{GABA_0} and G^E_0 are the default values presented in Table S3.

Then, we substitute J_{GABA} with equation 16 in 12, obtaining:

$$G^E = \frac{I^E_{fixed} + (J_{GABA_0} + G^E_0) S^I_{fixed}}{S^I_{fixed} + W^E I_0 + w_+ J_{NMDA} S^E_{fixed} + I^{ext}} \quad [17]$$

Synaptic Scaling of Excitatory and Inhibitory Synapses and Plasticity of Intrinsic Excitability. In line with the methodology implemented for the Wilson-Cowan model, we consider the homeostasis of excitation and inhibition in conjunction with the two forms of plasticity of intrinsic excitability. In this case, since the order of magnitude of J_{GABA} and G^E is much lower than b^E or a^E (see Table S3), to consider similar time scales for all homeostatic mechanisms we instead assume that their **relative** variation is the same, as opposed to absolute variation as done in (1). Therefore, when implementing the homeostasis of b^E in conjunction with the two forms of synaptic scaling we have:

$$\begin{aligned} \frac{J_{GABA} - J_{GABA_0}}{J_{GABA_0}} &= \frac{G^E_0 - G^E}{G^E_0} = \frac{b^E - b^E_0}{b^E_0} \\ J_{GABA} - J_{GABA_0} &= G^E_0 - G^E = \frac{b^E - b^E_0}{b^E_0} \end{aligned} \quad [18]$$

since $J_{GABA_0} = G^E_0 = 1$, which simplifies the expression.

That said, G^E and J_{GABA} can be substituted in the expression for I^E, yielding:

$$I^E = \left(G^E_0 - \frac{b^E - b^E_0}{b^E_0}\right)(W^E I_0 + w_+ J_{NMDA} S^E_{fixed} + I^{ext}) - \left(J_{GABA_0} + \frac{b^E - b^E_0}{b^E_0}\right) S^I_{fixed} \quad [19]$$

The value of b^E can then be obtained by solving equation 15 for b^E.

Similarly, for a^E, we have

$$J_{GABA_0} - J_{GABA} = G^E - G_0^E = \frac{a^E - a_0^E}{a_0^E} \quad [20]$$

which can be substituted in the I^E equation, yielding:

$$I^E = \left(G_0^E + \frac{a^E - a_0^E}{a_0^E} \right) (W^E I_0 + w_+ J_{NMDA} S^E_{fixed} + I^{ext}) - \left(J_{GABA_0} - \frac{a^E - a_0^E}{a_0^E} \right) S^I_{fixed} \quad [21]$$

which can be used with 15 to obtain the homeostatic value of a^E.

Modulation of Local Circuit Dynamics by E-I Homeostasis in the Wong-Wang Model. To analyze the effects of E-I homeostasis at the local circuit level in the Wong-Wang Model we explore the behavior of the model under different combinations of target firing rate $\rho = r^E_{fixed}$ and external input I^{ext} and distinct mechanisms of E-I homeostasis. For each combination of ρ and I^{ext}, we compute the homeostatic values of the parameters of interest, following the expressions in the previous section, and follow up with linear stability analysis to investigate circuit dynamics (5, 6). To do that, it is first necessary to compute the Jacobian J of the system:

$$J = \begin{bmatrix} \frac{df(t)}{dS^E} & \frac{df(t)}{dS^I} \\ \frac{dg(t)}{dS^E} & \frac{dg(t)}{dS^I} \end{bmatrix} \quad [22]$$

where $f(t) = \frac{dS^E}{dt}$ and $g(t) = \frac{dS^I}{dt}$.

The entries of the Jacobian can be calculated as follows:

$$\begin{aligned} \frac{df}{dS^E} &= -\frac{1}{\tau^E} + (1 - S^E) \left(\gamma G^E w_+ J_{NMDA} \frac{dF^E(I^E)}{dI^E} \right) - \gamma r^E \\ \frac{df}{dS^I} &= -(1 - S^E) \gamma J_{GABA} \frac{dF^E(I^E)}{dI^E} \\ \frac{dg}{dS^E} &= J_{NMDA} \frac{dF^I(I^I)}{dI^I} \\ \frac{dg}{dS^I} &= -\frac{1}{\tau^I} - \frac{dF^I(I^I)}{dI^I} \end{aligned} \quad [23]$$

where

$$\frac{dF^{E/I}}{dI^{E/I}} = \frac{a^{E/I}(1 - \exp(-d^{E/I}(a^{E/I} I^{E/I} - b^{E/I}))) - d^{E/I} a^{E/I} (a^{E/I} I^{E/I} - b^{E/I}) \exp(-d^{E/I}(a^{E/I} I^{E/I} - b^{E/I}))}{(1 - \exp(-d^{E/I}(a^{E/I} I^{E/I} - b^{E/I})))^2} \quad [24]$$

Then, the local dynamical regimes can be evaluated by analyzing the trace and determinant of the Jacobian (5, 6). We present the results of this analysis for all homeostatic mechanisms in Figure S28. In the case of the Wong-Wang model, it can be observed that E-I homeostasis does not have the same effect on local dynamics as for the Wilson-Cowan model (e.g. Figure S1). More specifically, there is no Hopf-bifurcation in the Wong-Wang model and, thus, regardless of the effects of homeostasis, the local circuits remain in a stable regime. Even though the plasticity of J^{GABA} can lead to the emergence of a stable spiral attractor (Figure S28B), with transient rhythms in response to perturbations, the dynamics still correspond to a stable attractor and, therefore, this transition is not considered a bifurcation.

Analysis of FC Properties in Lesion Simulations. To analyze the impact of structural lesions on FC and the subsequent recovery through E-I homeostasis, we follow the procedure introduced in (7). To analyze changes in FC patterns, we make use of FC distance, which roughly quantifies the magnitude of disruption, following (7, 8). Furthermore, to measure disruptions in the macroscale architecture of FC, we measure the correlation between structural and functional connectivity (9) and modularity (10, 11), both of which are impacted in stroke patients. Furthermore, the recovery of modularity is thought to be relevant for cognitive function (11). Below, we describe how each of these metrics is computed from simulated FC matrices. The simulation protocol is described in the Methods section of the main text.

FC Distance. To measure the dissimilarity between FC matrices at baseline, acute, and chronic periods, we follow (8), defining FC distance as the Frobenius norm of the difference between two given matrices.

$$distance(FC_1, FC_2) = \sqrt{\sum_{i,j} (FC_2 - FC_1)^2_{ij}} \quad [25]$$

FC-SC Correlation. Given the results of (9), showing a decoupling between functional and structural connectivity in stroke patients, correlating with motor function, we test this biomarker at baseline, acute, and chronic periods, by computing the Pearson's correlation coefficient between the upper triangles of FC and SC matrices.

Modularity. Modularity measures the degree to which a network follows a modular structure, with dense connections within functional clusters and sparser ones between them. Modularity (Q) was calculated using the formula defined in (12):

$$Q = \sum_{u \in M} [e_{uu} - (\sum_{v \in M} e_{uv})^2] \quad [26]$$

where M is a set of non-overlapping modules (groups of nodes) in the network and e_{uv} is the proportion of edges in the network that connect nodes in module u with nodes in module v. Similarly to previous studies, (7, 11), we define modules *a priori* to avoid biasing the modularity measure by using modularity maximization to detect community structure. That said, modules were from the empirical FC data, by using a clustering algorithm described in (7), resulting in 6 clusters. Following the original formulation of modularity (12), FC matrices were transformed into unweighted graphs by applying a density threshold, through which only a percentage of the strongest connections are kept and their weights set to 1. Lesioned nodes were removed from the network before computing modularity, similarly to (7, 11).

Model Validity for Different Mechanisms of Homeostasis. For each type of homeostasis, we run short simulations (15 seconds) with all combinations of parameters and evaluate the deviation of the mean node activity from the target firing rate ρ. If any node deviates by more than 1%, we consider the fixed point invalid.

To start, we illustrate the impact of each parameter on model validity for models under G^E homeostasis and discuss the principles underlying the ability of networks to maintain target firing rates in different regions of the parameter space represented by the combination of the free parameters (C, rho and mean delay). We start by using models with G^E homeostasis to illustrate the different behaviors of our model (Figure S2). However, the general principles apply to other types of homeostasis, as will be illustrated later. The respective parameter spaces can be consulted in Figure S7.

The first main conclusion is that, as the target firing rate ρ increases, the models become increasingly unable to maintain firing rates at the target and, for any value of ρ higher than 0.15, no combination of parameters resulted in a valid system. This result is easily interpreted. As more nodes enter the limit cycle regime (S1) the presence of sustained oscillations leads to a tendency of nodes to synchronize in a self-reinforcing manner, generating run-away activity and resulting in the instability of the fixed point solution. Therefore, to guarantee the stability of models, the target firing rate ρ should be close to the threshold after which some nodes enter the limit cycle regime, corresponding to $\rho = 0.12$.

To delve into this topic in more detail, we examine the behavior of the model when $\rho = 0.14$. While most of the parameter space corresponds to an invalid solution of the system, systems can maintain mean firing rates if the global coupling is high enough (Figure S2). To study this effect, we simulated models with the same ρ (0.12) and mean delay (10 ms), but with low ($C = 0.75$) and high ($C = 7.5$) global coupling (Figures S3 and S4, respectively). For each, we ran simulations with the predicted homeostatic G^E, no noise, and different initial conditions for node activity r^E (0, ρ or 2ρ). In addition, we ran simulations with dynamical G^E homeostasis (obeying $\tau_{homeo}\frac{dG^E}{dt} = -(r^E - \rho)$) and different homeostatic timescales, to ensure that the instabilities were inherent to the model and not a consequence of errors in the estimation of homeostatic parameters. Starting with the lower global coupling ($C = 0.75$) (Figure S3), we observe that, regardless of the initial conditions, the model with predicted G^E tends toward global synchronization and, since long-range connections are excitatory, the oscillations self-reinforce and quickly reach activity levels close to saturation. How fast this happens depends on the initial conditions. Can this be fixed by implementing explicit homeostatic plasticity of G^E, instead of using the predicted weights? Our results indicate that such models, having the same tendency to synchronize, will exhibit short periods of high activity departing from ρ, which are then quenched by homeostatic plasticity. In this case, the speed of this quenching depends on the timescale of homeostatic plasticity (Figure S3). For this reason, we consider these models to still be invalid, indicating that the inability to maintain mean firing rates is an inherent property of systems with this combination of parameters and not due to errors in the estimation of parameters. Indeed, when observing the behavior of individual nodes in the homeostatic fixed points (Figure S3), we see that 52 of them are in the limit cycle regime, which explains the tendency of the model for over-synchronizing.

But why are models with $\rho = 0.14$ when the global coupling is increased? In Figure S4, we show one such case where, regardless of the initial conditions, all nodes in the model converge toward the target firing rate. Following the principles introduced before, we show that the homeostatic solution to this system corresponds to only one node in the limit cycle regime. This phenomenon occurs due to how model dynamics are shaped by G^E homeostasis (Figure S4). Shortly, for a given value of ρ, as the external current is increased, the models transition from a limit cycle to a stable spiral regime. Therefore, given that I^{ext} scales with the global coupling, increasing the global coupling will bring nodes out of the limit cycle regime, contradicting the tendency to hypersynchrony. This effect is only present for some types of homeostasis (Figure S1) which has implications for the validity of models. Conversely, in models with μ^E or c^{EI} homeostasis, when $\rho \geq 0.12$ all nodes are necessarily in the limit cycle regime and, therefore, have a greater tendency toward instability.

That said, it should be pointed out that, for target firing rates lower than 0.12, the general effect of increasing the global coupling is the opposite, disrupting the ability of models to maintain mean firing rates at the target. However, in this case, the underlying phenomena are distinct. In Figure S5 we present the behavior of the model in one such case, where the model settles in different fixed points depending on the initial conditions. If the initial r^E is higher than the target firing rate, the model will settle in a stable configuration where mean activity is higher than ρ. Conversely, the opposite happens when the model is initialized with $r^E = 0$. More importantly, the target fixed point of the system is only stable when the initial conditions correspond to $r^E = \rho$ across all nodes. These results indicate that, for this combination of parameters, the model is bistable, with the target corresponding to the saddle point of the system, thus being unstable. Accordingly, when implementing explicit homeostatic plasticity (Figure S5), the system will constantly switch between the two stable attractors. Since the target firing rate is different from ρ in both, homeostatic plasticity will be permanently frustrated, leading to the constant switching between attractors. For this reason, even though the model can be stable in this case, the stable solutions do not correspond to the target firing rate and, therefore, we consider this to represent an invalid solution of the system. While we performed this analysis in the model with G^E homeostasis, the same principles apply, for example, to models with $G^E + c^{EI}$ homeostasis (Figure S10).

Our results so far suggest that the main parameters shaping the ability of models to maintain the target firing rate are the global coupling and ρ. However, there are instances where the mean delay also plays a significant role. As an example, we take $C = 4.5$ and $\rho = 0.11$ (close to the bifurcation after which nodes with weaker inputs start entering the limit cycle regime) and examine model activity for 4 different values of mean delay (Figure S6). Starting with the model with no delays (mean delay=0), we observe that the system tends toward hyper-synchrony, similar to what was described for high values of ρ. As the mean delay is increased to higher values, this tendency disappears and the model can settle in the target fixed point. Here, our results demonstrate the role of delayed interactions in avoiding widespread synchronization, through the disruption of

phase-relations between oscillating systems. However, we observe a range of mean delays centered around 26 milliseconds, for which the model can also be invalid (Figure S6). In this case, the target fixed point is only weakly stable, since, when approached from one direction, dynamics settle at ρ. However, if the initial conditions are higher than the target, some of the nodes engage in sustained oscillations potentiated by their recurrent interactions. While this case is not as severe as the model with no delays, it still represents a solution of the system that does not correspond to the target ρ. But why does this happen specifically for this range of delays? For this combination of C and ρ, most of the nodes have a natural frequency of oscillation between 45 and 60 Hz (Figure S6). More importantly, the nodes with the lower input, which are the ones closer to the limit cycle regime (S1), oscillate at around 45 Hz, corresponding to a period of ~22.5 ms. Therefore, when the mean delay is close to this value, nodes in the system can still synchronize, albeit with a phase shift close to a full cycle, which is sufficient to throw the system outside of the desired equilibrium. Accordingly, as the mean delay is further increased, the models become valid again (Figure S6). While we performed this analysis in the model with G^E homeostasis, the same principles apply, for example, to models with c^{EI} (Figure S8) or $G^E + c^{EI}$ (Figure S9) homeostasis.

Finally, for each mechanism of homeostasis, we plot the percentage of valid simulations as a function of each free parameter (C, ρ, and mean delay) and the percentage across all simulations (Figure S11). Our results suggest that, across the various mechanisms of homeostasis, the main parameters shaping model validity are the global coupling C and target firing rate ρ. However, validity is affected in models with homeostasis of inhibition (c^{EI}) (19.26% valid simulations), firing threshold (μ^E) (14.80%), or the combined homeostasis of G^E, c^{EI} and μ^E (20.47%), all of which strongly rely on the modulation of "additive/subtractive" model parameters (1). Conversely, homeostasis mechanisms involving excitatory synapses (G^E) or the slope of neural excitability (σ^E) (i.e. "multiplicative parameters") enhance model validity (Figure S11D). We suggest that this results from the modulation of the bifurcation point toward higher ρ in response to increases in external inputs, which is characteristic of these mechanisms of homeostasis (1) (Fig S15). More specifically, when C is increased, amplifying not only the magnitude of external inputs, but also their fluctuations (Fig S16), nodes can compensate for the stronger input fluctuations by being farther away from the bifurcation. Conversely, with the homeostasis of c^{EI} or μ^E, nodes with higher inputs are more likely to engage in sustained oscillations (Fig S15), enhancing network instabilities. In short, the bifurcation modulation effect afforded by the homeostasis of excitation and the slope of intrinsic excitability is an important feature of local cortical circuits, ensuring that the target firing rate can be maintained across the network.

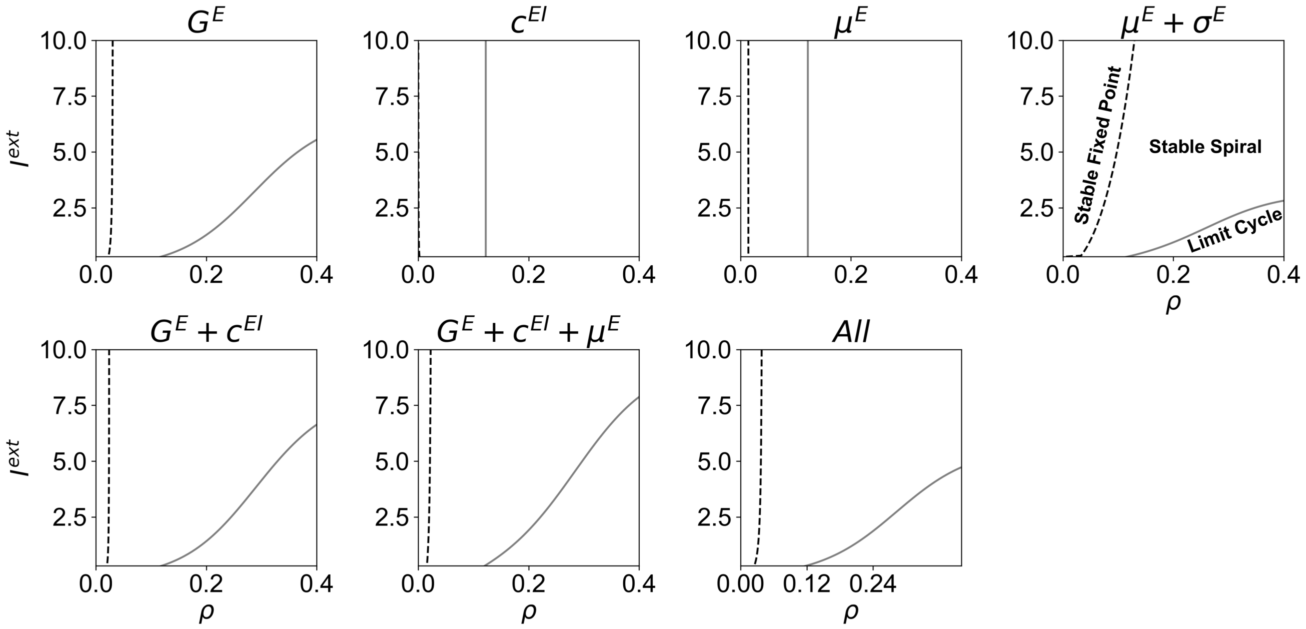

Fig. S1. Phase portraits of the Wilson-Cowan model under different mechanisms of homeostasis Dashed lines represent the transition between the stable fixed point and stable spiral regimes and solid lines represent the Andronov-Hopf bifurcation between damped and sustained oscillations (i.e. limit cycle). Refer to (1) for more detail on the formulation of each mechanism of homeostasis and the analysis of the dynamics of Wilson-Cowan nodes under the distinct mechanisms of homeostasis.

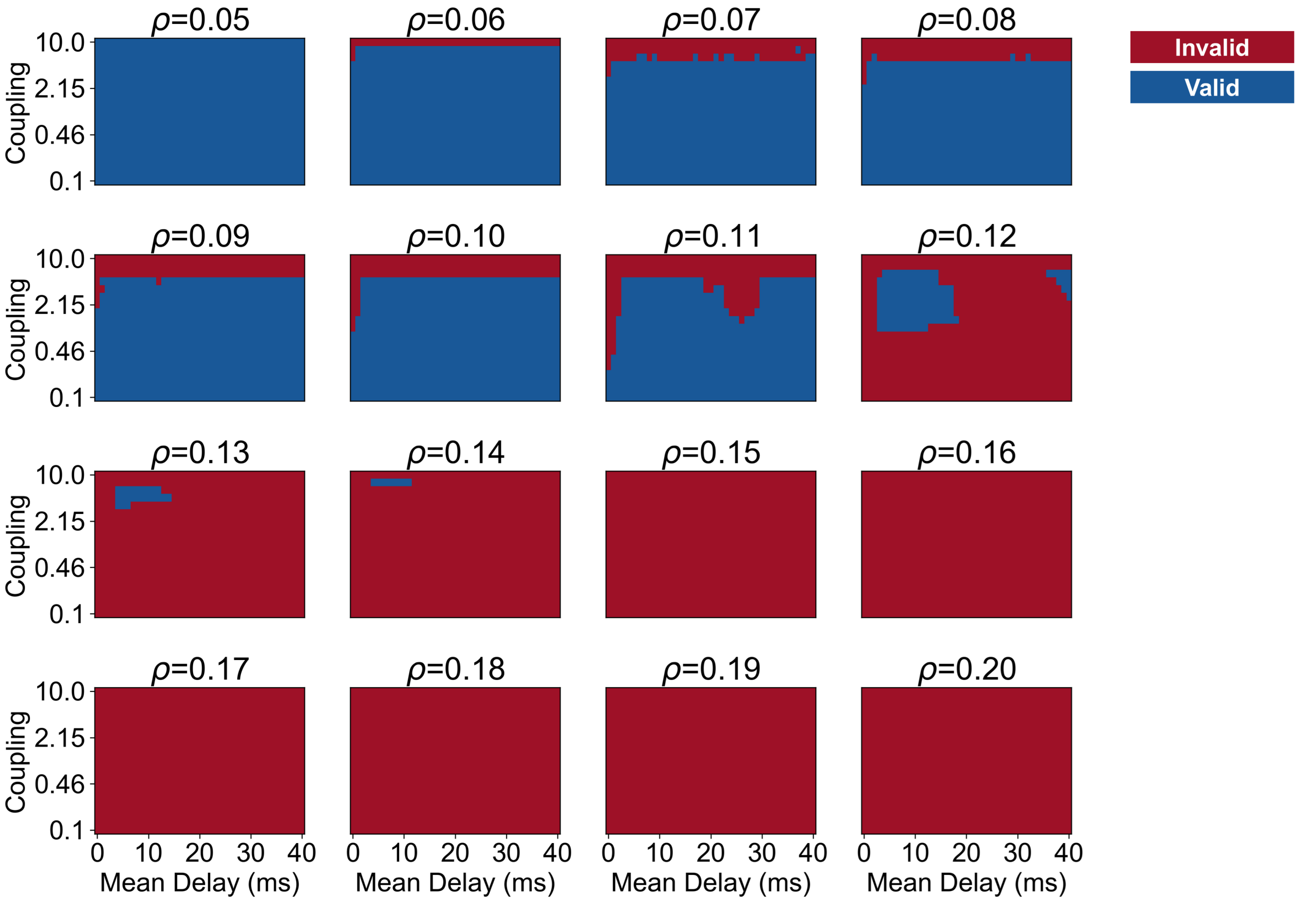

Fig. S2. Model validity under different combinations of parameters for G^E Homeostasis Red represents invalid models, where the mean activity of at least one of the nodes differed from the target firing rate ρ by more than 1%. Conversely, blue represents models where all nodes have mean activity close to the target.

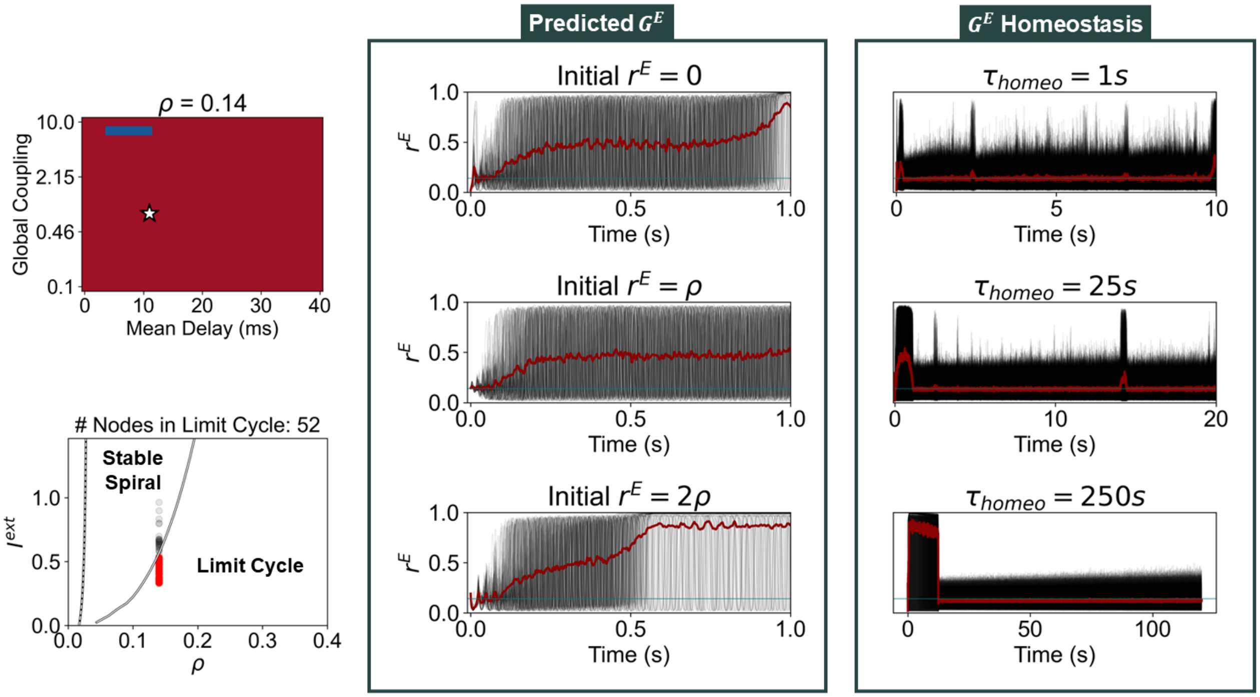

Fig. S3. Network Behavior for $C = 0.75$, $\rho = 0.14$ and mean delay 10 ms On the top left, we present the stability parameter space for $\rho = 0.14$, where the star represents the current parameters. On the bottom left, we present the dynamical portrait for G^E homeostasis at node level, with red and black dots representing network nodes in the limit cycle and stable spiral regimes, respectively. On the right, we present the activity of networks with predicted G^E values, for different initial conditions, and of networks with dynamical G^E homeostasis with different time constants. Black lines represent r^E of each node, red lines the average across nodes and blue lines the target firing rate ρ.

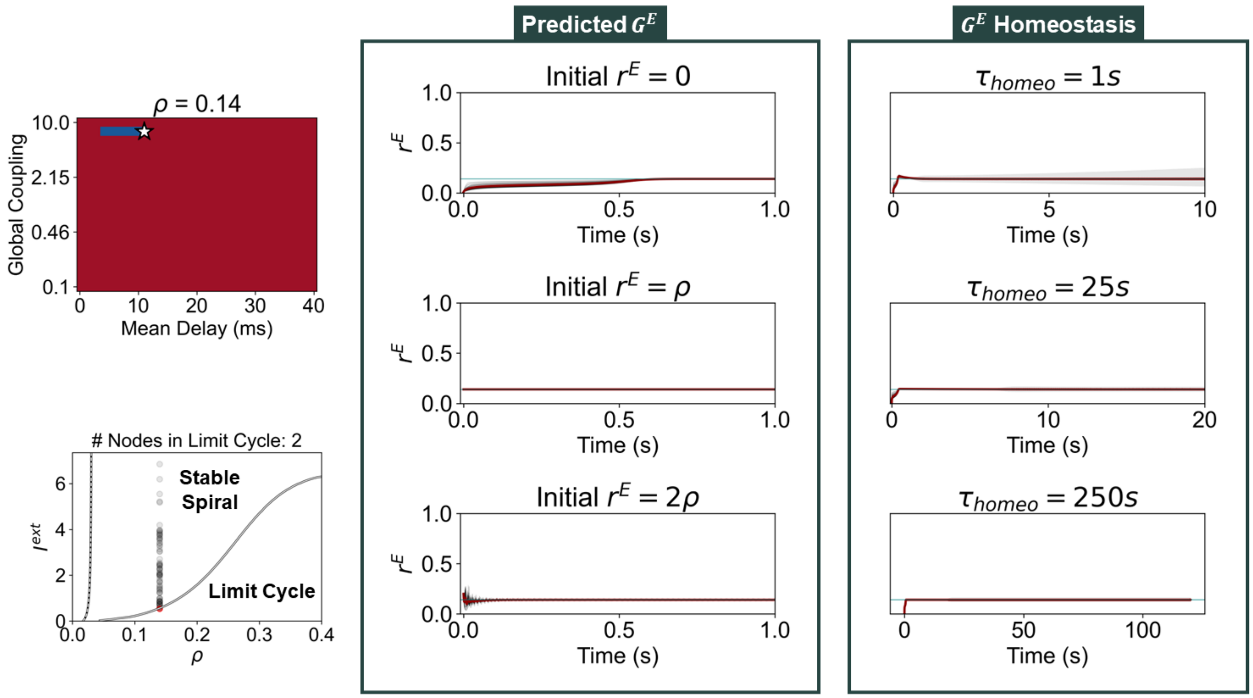

Fig. S4. Network Behavior for $C = 7.5$, $\rho = 0.14$ and mean delay 10 ms

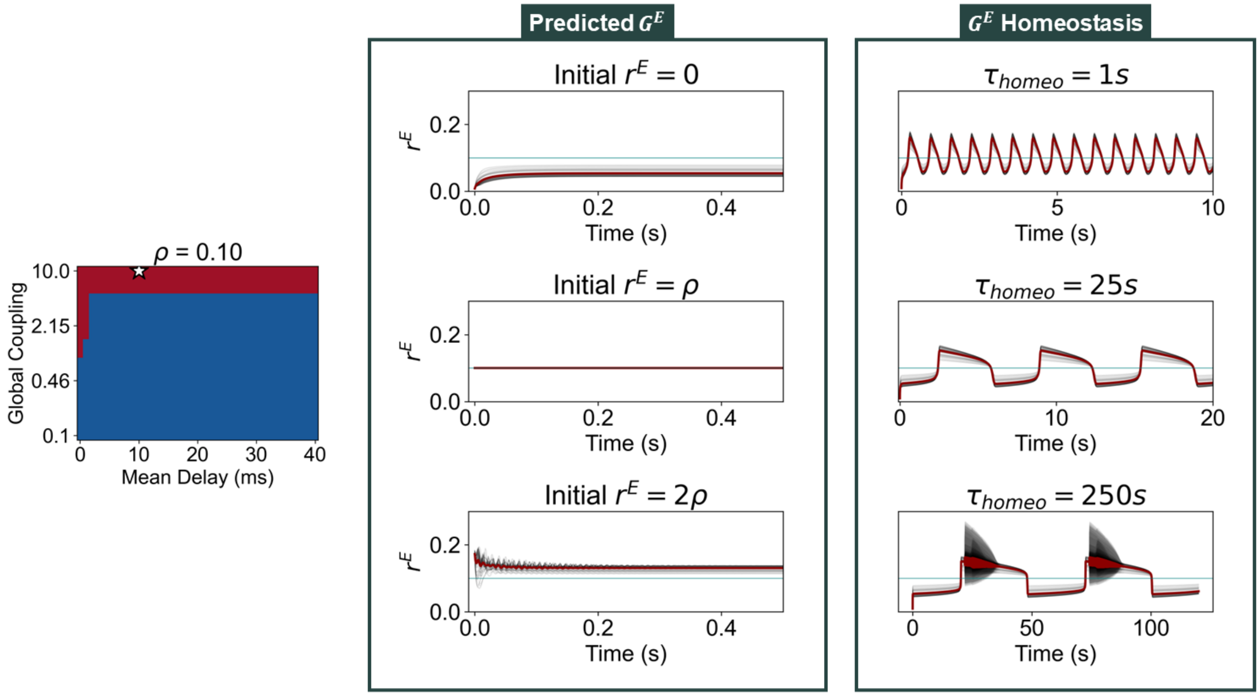

Fig. S5. Network Behavior for $C = 10$, $\rho = 0.10$ and Mean Delay 10 ms.

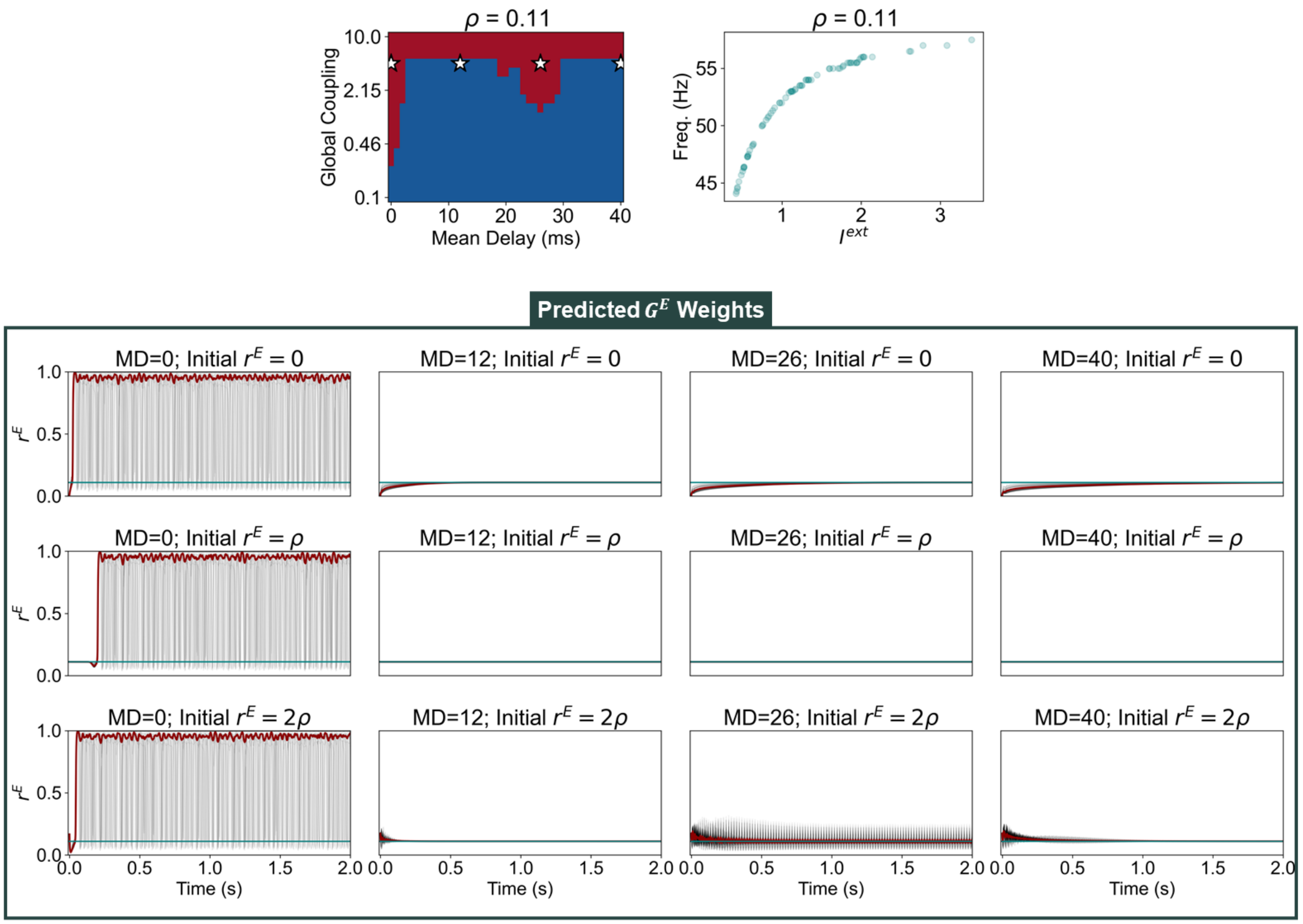

Fig. S6. Network behavior for $C = 4.5$, $\rho = 0.11$ and different mean delays. On the top left, we present the validity parameter space of the model for $\rho = 0.11$. On the top right, we present the natural frequency of oscillation of nodes in the network as a function of the average external input they receive. On the bottom, we display network activity for models with mean delay 0, 12, 26 and 40 ms, and with different initial conditions. Black lines represent r^E of each node, red lines the average across nodes and blue lines the target firing rate ρ.

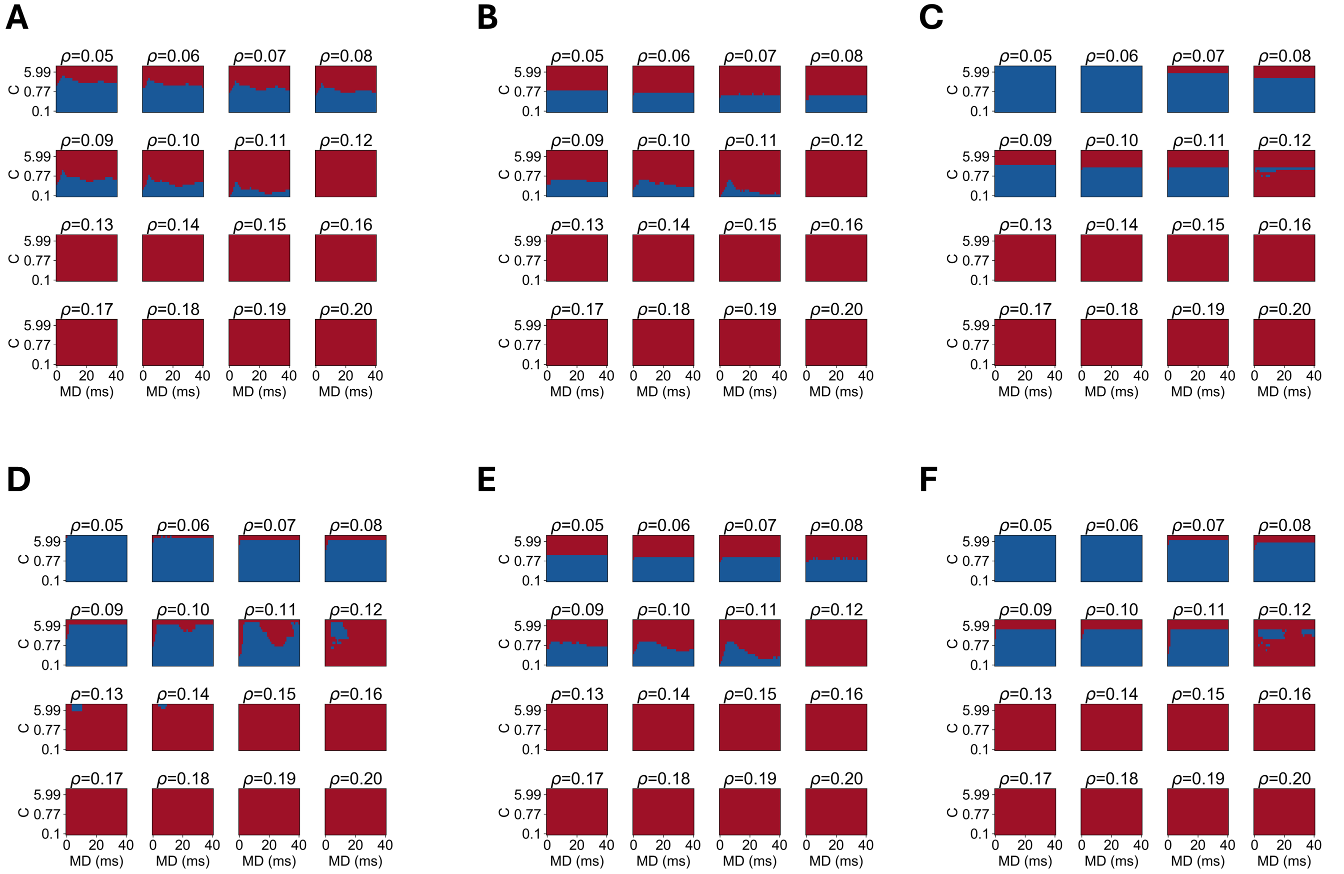

Fig. S7. Validity of models with different mechanisms of homeostasis (A) c^{EI} Homeostasis (B) μ^{E} Homeostasis (C) $\mu^{E}+\sigma^{E}$ Homeostasis (D) $G^{E}+c^{EI}$ Homeostasis (E) $G^{E}+c^{EI}+\mu^{E}$ Homeostasis (F) $G^{E}+c^{EI}+\mu^{E}+\sigma^{E}$ Homeostasis. Blue and red colors represent valid and invalid simulations, respectively.

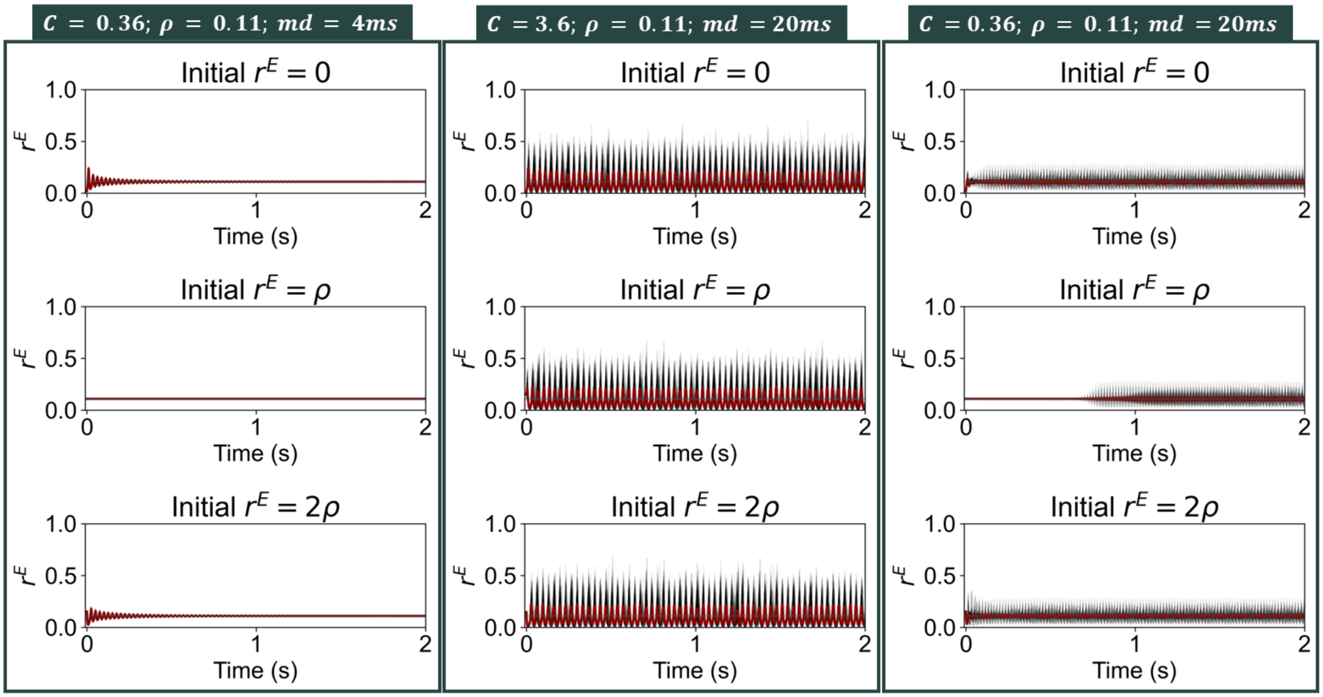

Fig. S8. Behavior of the network with homeostasis of c^{EI}. We present the activity of models with predicted c^{EI} values and different initial conditions: $r^E = 0$ (Top), $r^E = \rho$ (Middle) and $r^E = 2\rho$ (Bottom)

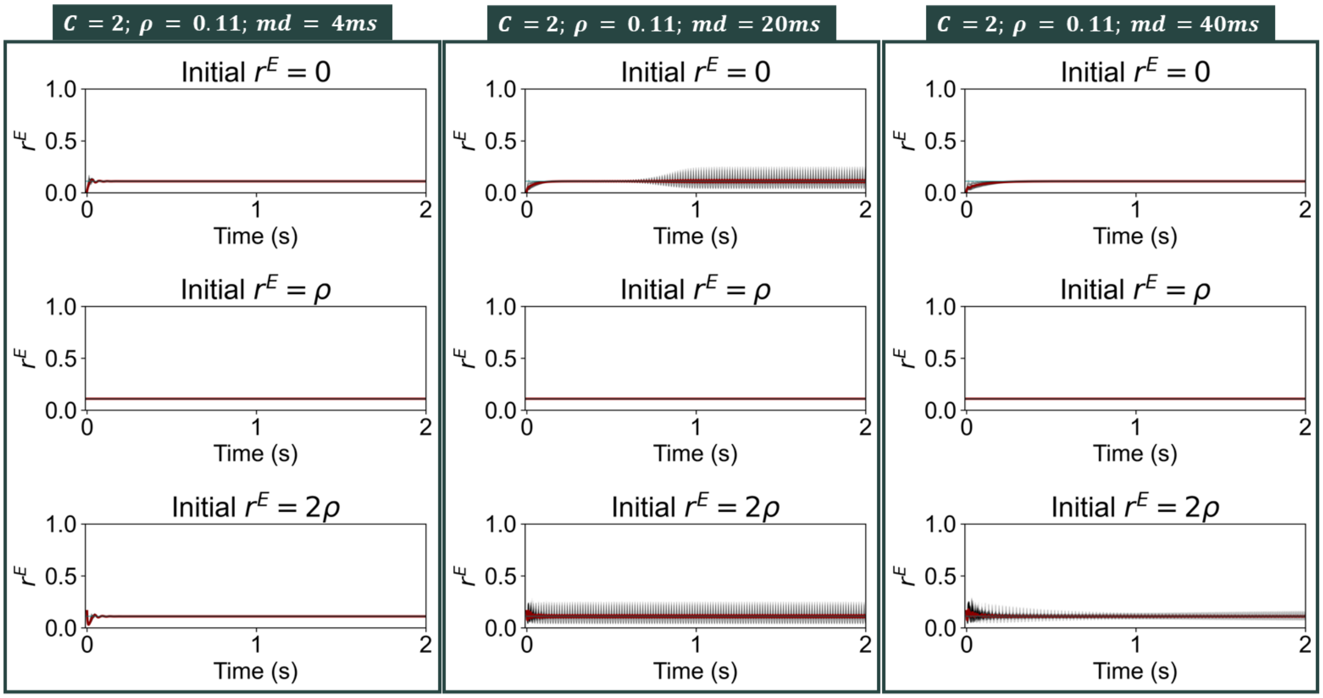

Fig. S9. Behavior of the network with homeostasis of G^E and c^{EI}. We present the activity of models with predicted parameters and different initial conditions: $r^E = 0$ (Top), $r^E = \rho$ (Middle) and $r^E = 2\rho$ (Bottom)

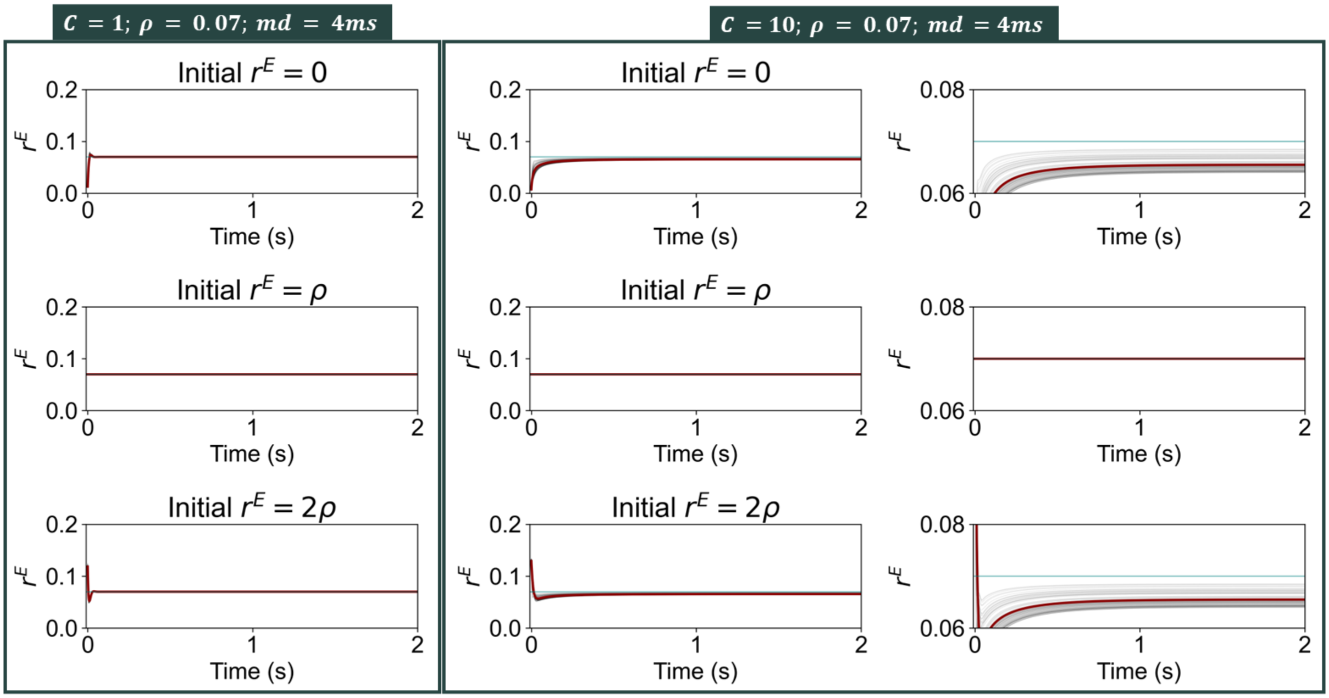

Fig. S10. Behavior of the network with homeostasis of G^E and c^{EI}. We present the activity of models with predicted parameters and different initial conditions: $r^E = 0$ (Top), $r^E = \rho$ (Middle) and $r^E = 2\rho$ (Bottom)

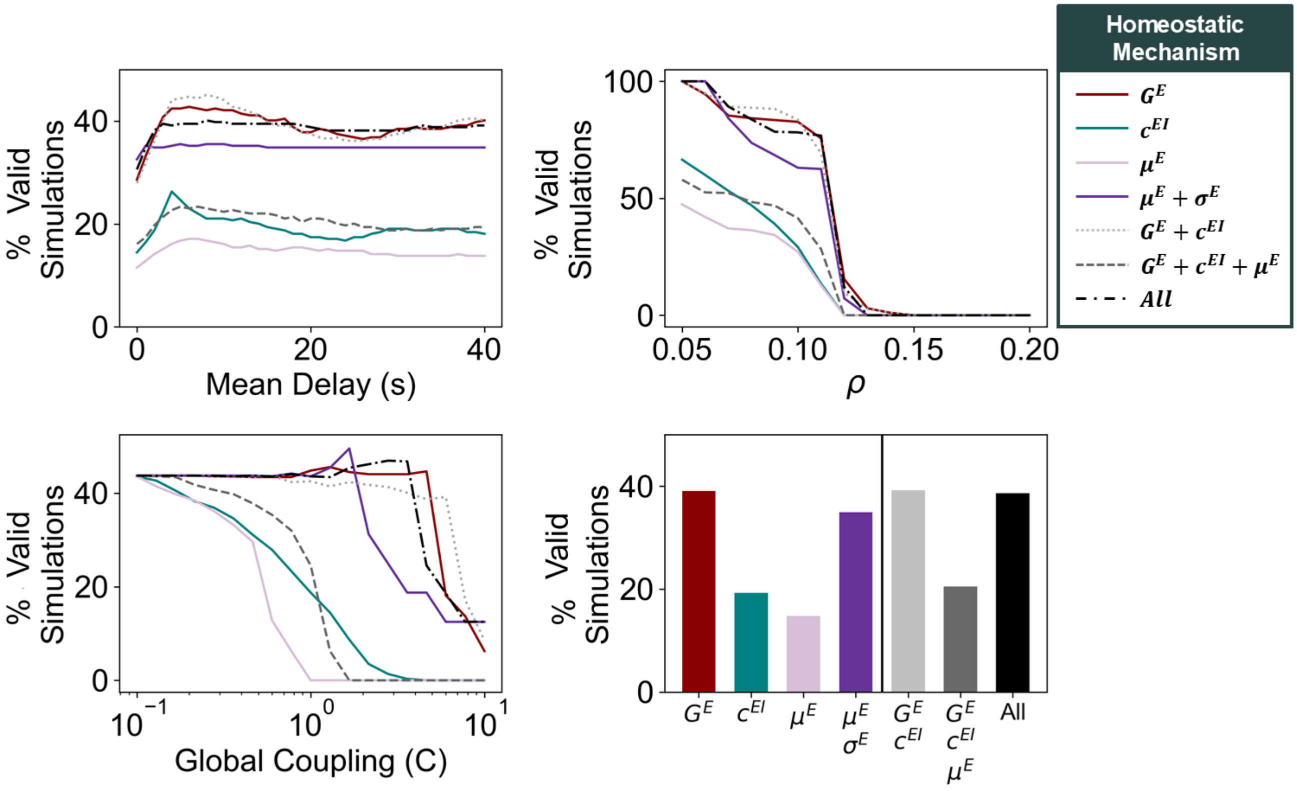

Fig. S11. Model validity for different mechanisms of homeostasis (A) Percentage of valid simulations as a function of the mean delay. Each line represents a different mechanism of E-I homeostasis. (B) Percentage of valid simulations as a function of ρ (C) Percentage of valid simulations as a function of C. (D) Percentage of valid simulations across all combinations of parameters for each mechanism of homeostasis

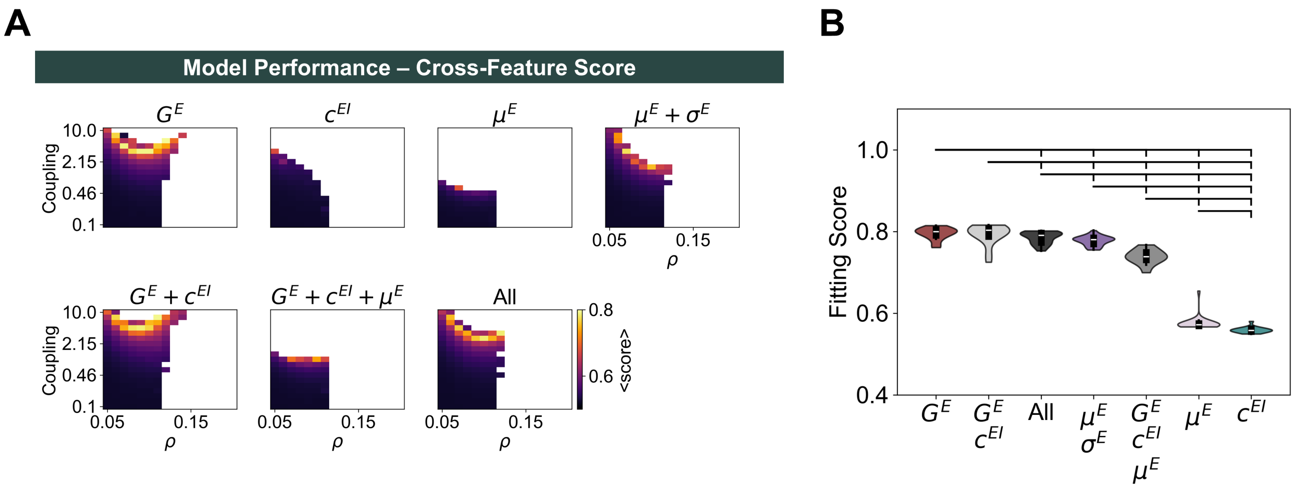

Fig. S12. Cross-feature model performance for different homeostatic mechanisms (A) Parameter spaces representing the cross-feature score for each combination of C and ρ, averaged across mean delays. Blank spaces represent combinations of C and ρ for which the homeostatic set point is not valid (i.e. mean firing rates differ from ρ by more than 1% in at least one cortical area) (B) Comparison between cross-feature fitting scores at the optimal point of each mechanism of homeostasis. Distributions correspond to simulations with optimal C and ρ and all values of mean delay yielding a valid network solution. Brackets indicate a significant difference, with p<0.05 from a Mann-Whitney U-test. All p-values were FDR-corrected. All scores in this figure were computed using the following formula: $\sqrt{\frac{r_{FC}^2+\left(\frac{MSE_{max}-MSE_{FC}}{MSE_{max}}\right)^2+(1-KS_{FCD})^2}{3}}$. This score represents the norm of a 3D vector where each component represents the performance of the model in representing one of the features of interest, normalized between 0 and 1. MSE_{max} represents the maximum possible mean squared error between our empirical FC matrix and a given FC matrix.

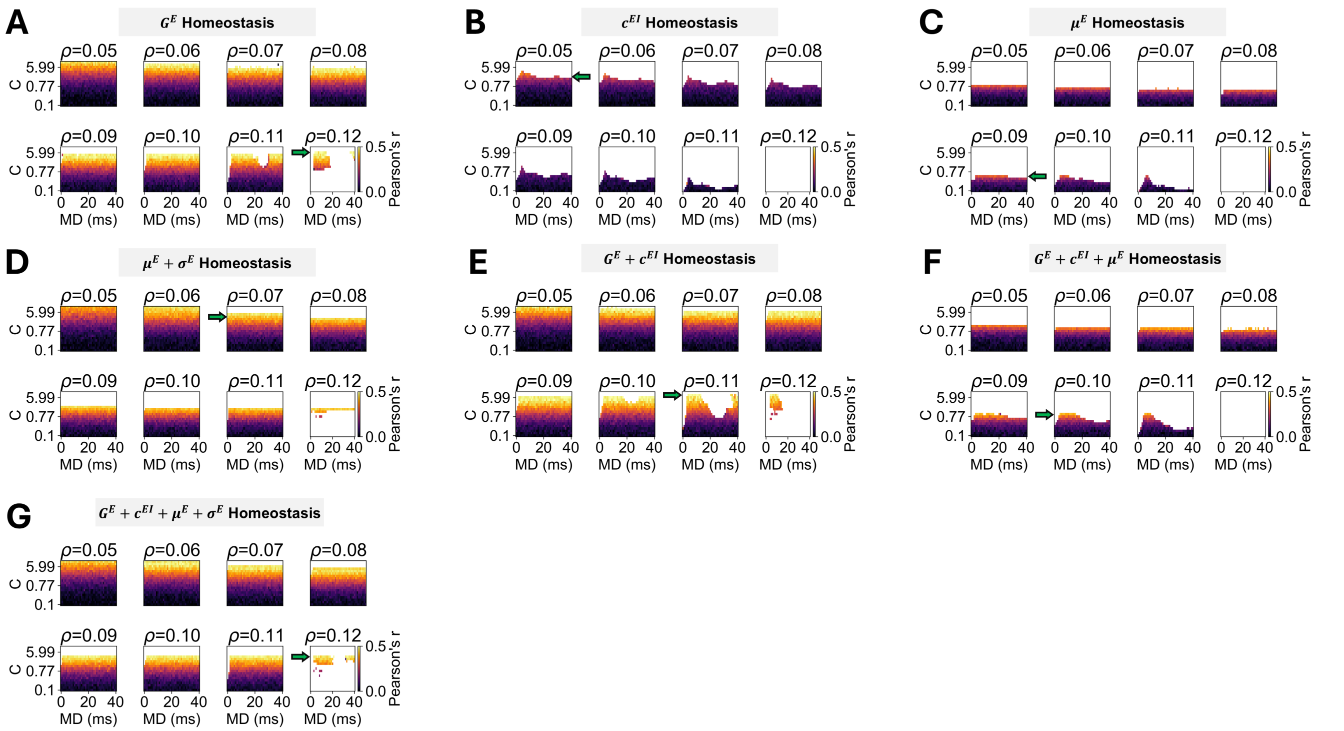

Fig. S13. FC fitting of models with different mechanisms of homeostasis (A) G^E Homeostasis (B) c^{EI} Homeostasis (C) μ^E Homeostasis (D) $\mu^E + \sigma^E$ Homeostasis (E) $G^E + c^{EI}$ Homeostasis (F) $G^E + c^{EI} + \mu^E$ Homeostasis (G) $G^E + c^{EI} + \mu^E + \sigma^E$ Homeostasis. Colors represent the correlation coefficient between empirical and simulated FC matrices for each combination of C and ρ.

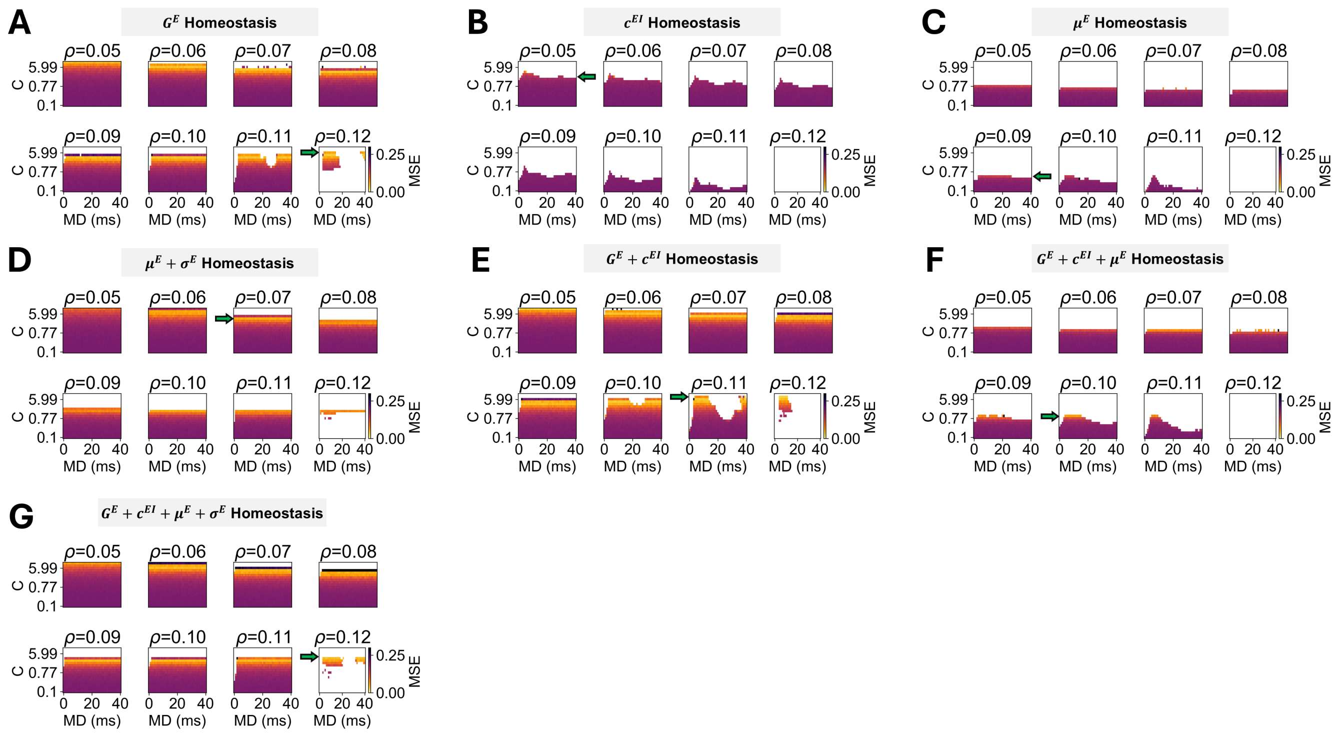

Fig. S14. FC fitting of models with different mechanisms of homeostasis (A) G^E Homeostasis (B) c^{EI} Homeostasis (C) μ^E Homeostasis (D) $\mu^E + \sigma^E$ Homeostasis (E) $G^E + c^{EI}$ Homeostasis (F) $G^E + c^{EI} + \mu^E$ Homeostasis (G) $G^E + c^{EI} + \mu^E + \sigma^E$ Homeostasis. Colors represent the mean squared error between empirical and simulated FC matrices for each combination of C and ρ.

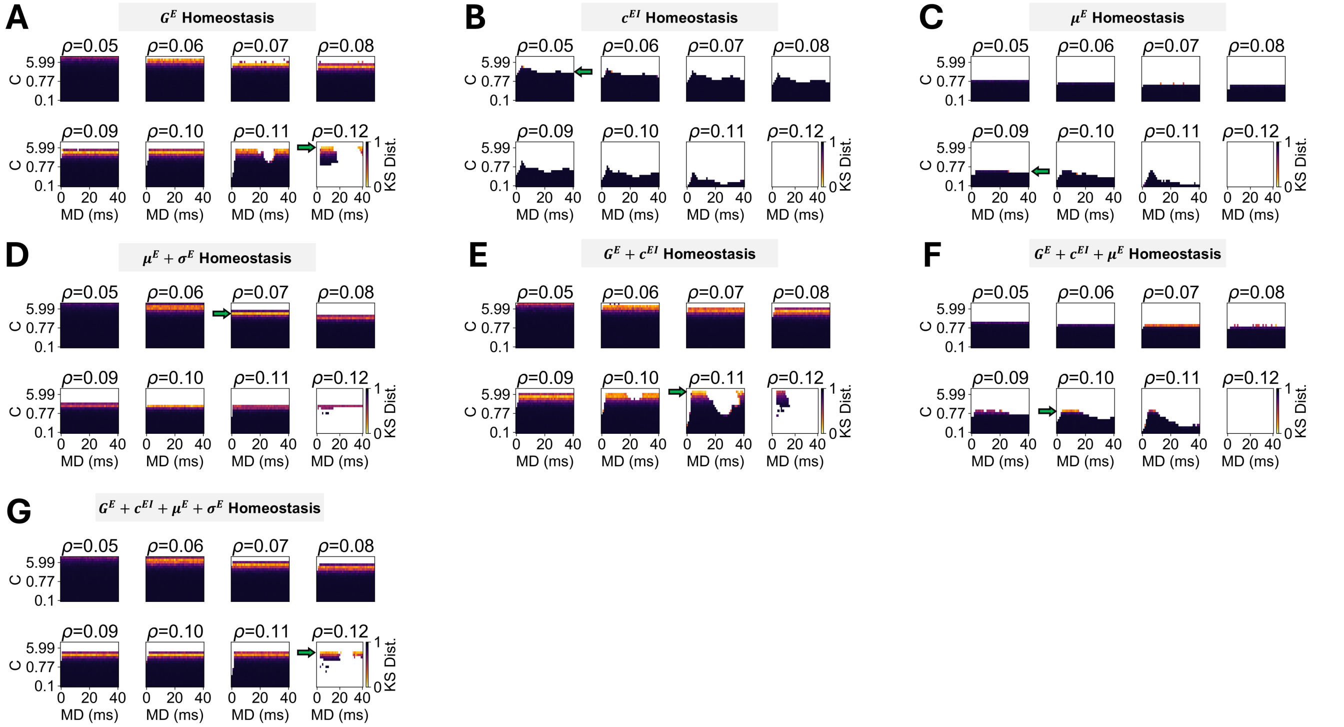

Fig. S15. FCD fitting of models with different mechanisms of homeostasis (A) G^E Homeostasis (B) c^{EI} Homeostasis (C) μ^E Homeostasis (D) $\mu^E + \sigma^E$ Homeostasis (E) $G^E + c^{EI}$ Homeostasis (F) $G^E + c^{EI} + \mu^E$ Homeostasis (G) $G^E + c^{EI} + \mu^E + \sigma^E$ Homeostasis. Colors represent the Kolmogorov-Smirnov distance between empirical and simulated FCD distributions for each combination of C and ρ.

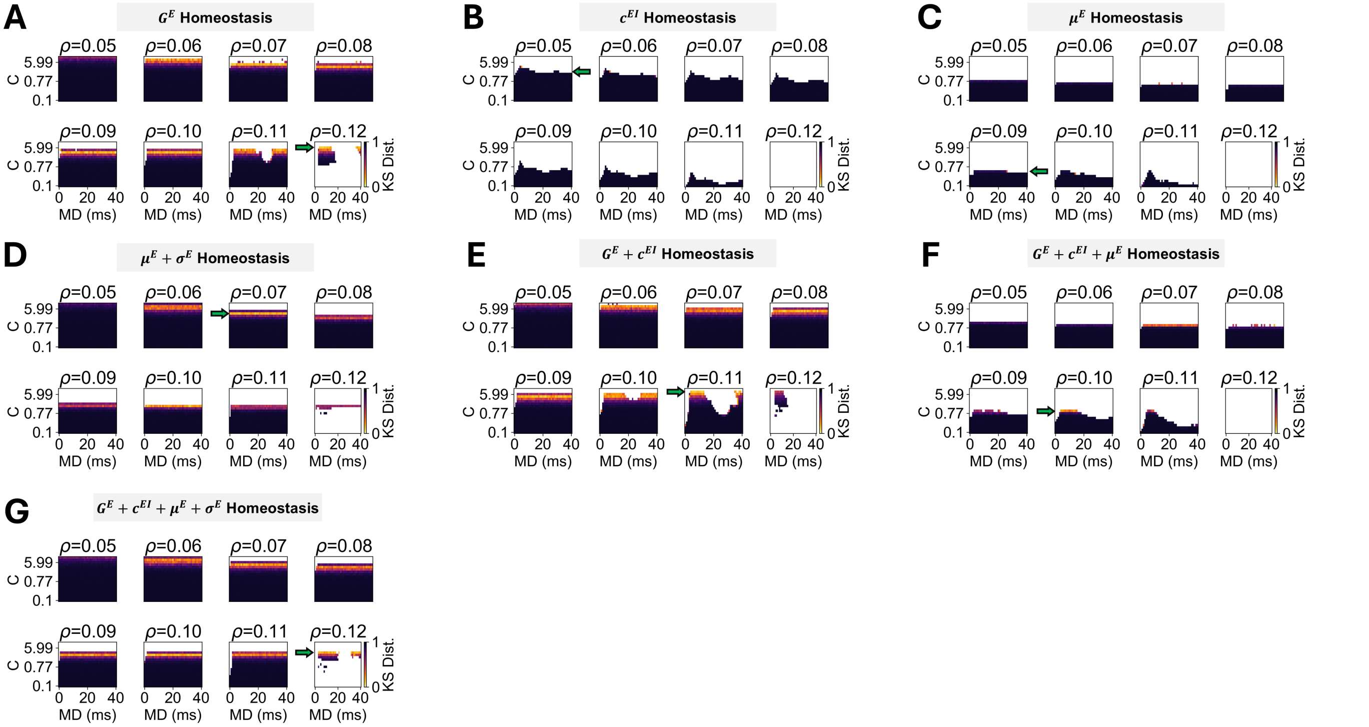

Fig. S16. Fitting performance of models with different mechanisms of homeostasis (A) G^E Homeostasis (B) c^{EI} Homeostasis (C) μ^E Homeostasis (D) $\mu^E + \sigma^E$ Homeostasis (E) $G^E + c^{EI}$ Homeostasis (F) $G^E + c^{EI} + \mu^E$ Homeostasis (G) $G^E + c^{EI} + \mu^E + \sigma^E$ Homeostasis. Colors represent the fitting score ($r_{FC} - MSE_{FC} - KS_{FCD}$) for each combination of C and ρ.

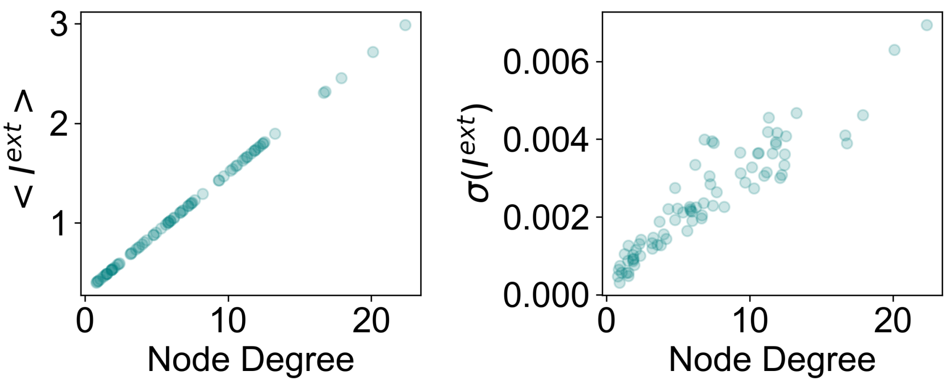

Fig. S17. Relationship between node degree, average inputs and input fluctuations. (A) Relationship between node degree (i.e sum of connectivity weights of a node) and the average input received by nodes. (B) Relationship between node degree (i.e sum of connectivity weights of a node) and fluctuations (i.e. standard deviation) of inputs received by a node. These results were obtained from a 60s simulation of the model with homeostasis of G^E, c^{EI} and $\mu^E + \sigma^E$ at the optimal point ($C = 3.59$, $\rho = 0.12$) and with a mean delay of 40 ms.

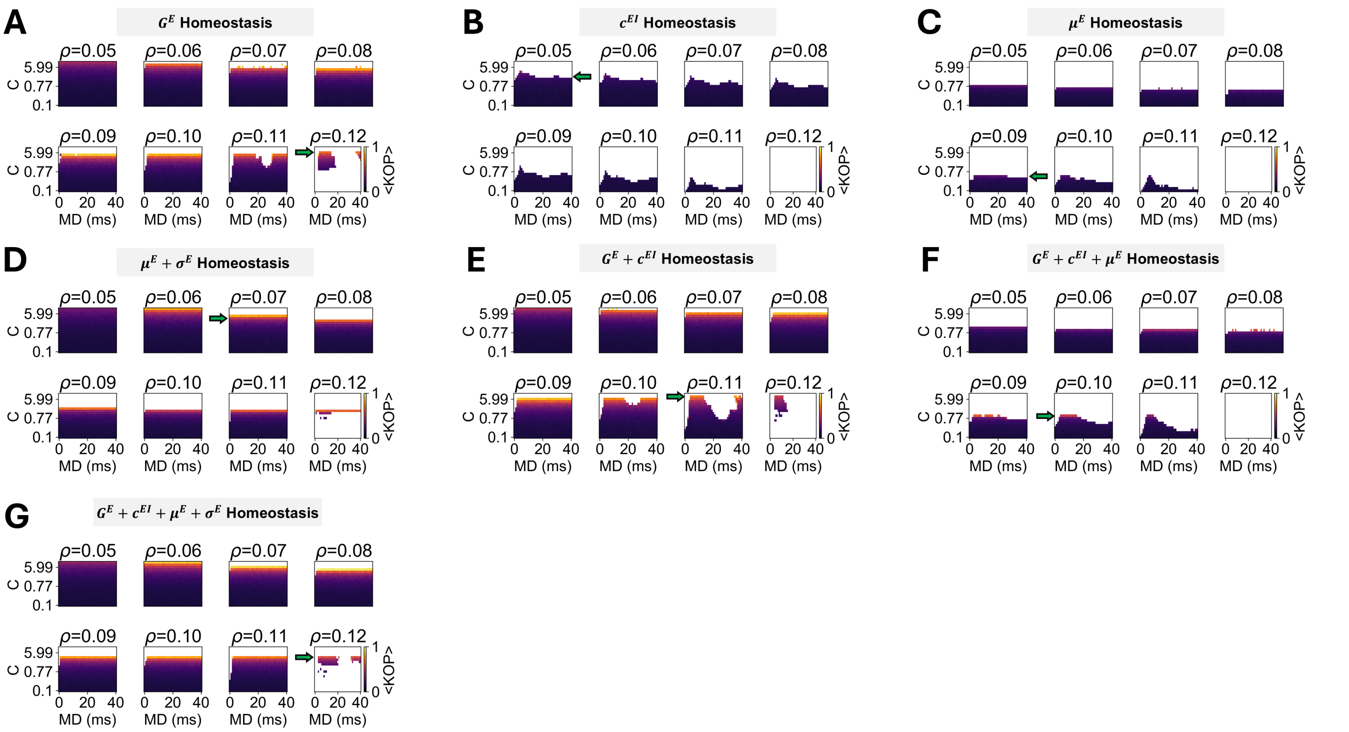

Fig. S18. Synchrony of models with different mechanisms of homeostasis (A) G^E Homeostasis (B) c^{EI} Homeostasis (C) μ^E Homeostasis (D) $\mu^E + \sigma^E$ Homeostasis (E) $G^E + c^{EI}$ Homeostasis (F) $G^E + c^{EI} + \mu^E$ Homeostasis (G) $G^E + c^{EI} + \mu^E + \sigma^E$ Homeostasis. Colors represent the mean of the Kuramoto Order Parameter (i.e. synchrony) for each combination of C and ρ.

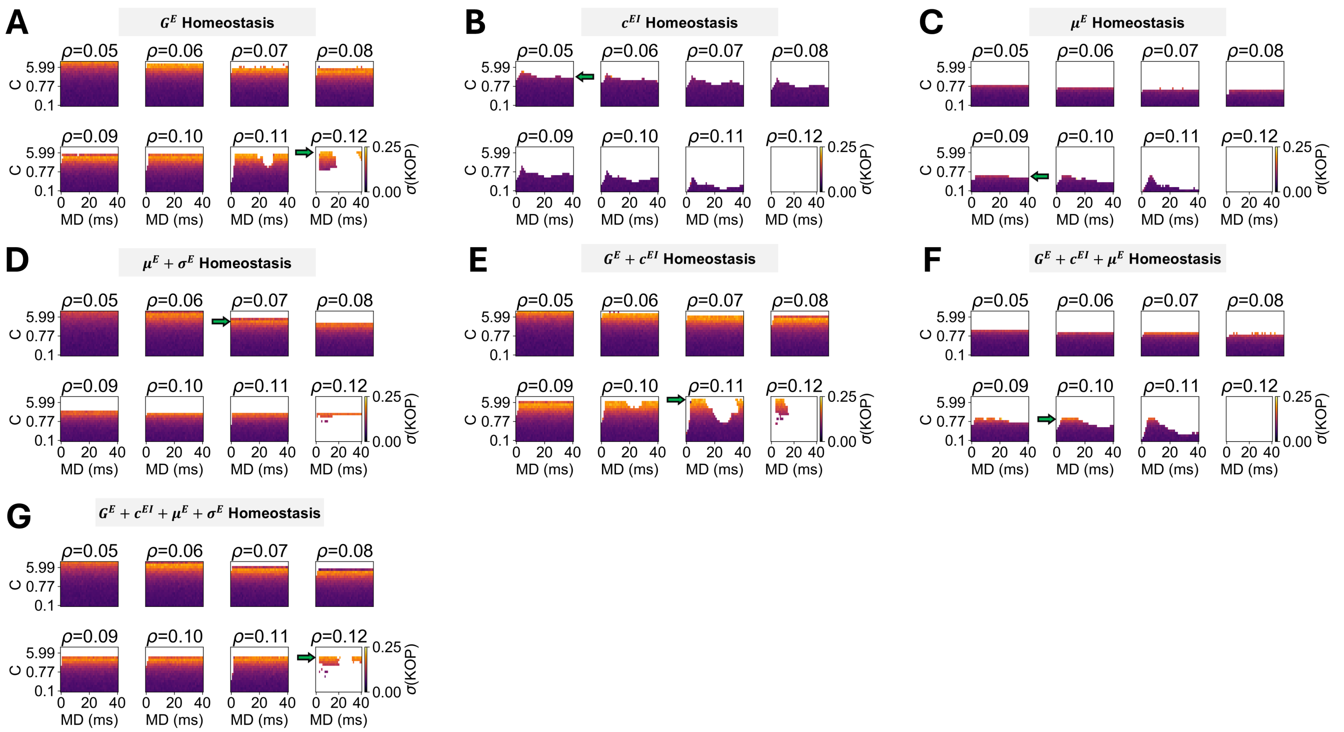

Fig. S19. Metastability of models with different mechanisms of homeostasis (A) G^E Homeostasis (B) c^{EI} Homeostasis (C) μ^E Homeostasis (D) $\mu^E + \sigma^E$ Homeostasis (E) $G^E + c^{EI}$ Homeostasis (F) $G^E + c^{EI} + \mu^E$ Homeostasis (G) $G^E + c^{EI} + \mu^E + \sigma^E$ Homeostasis. Colors represent the standard deviation of the Kuramoto Order Parameter (i.e. metastability) for each combination of C and ρ.

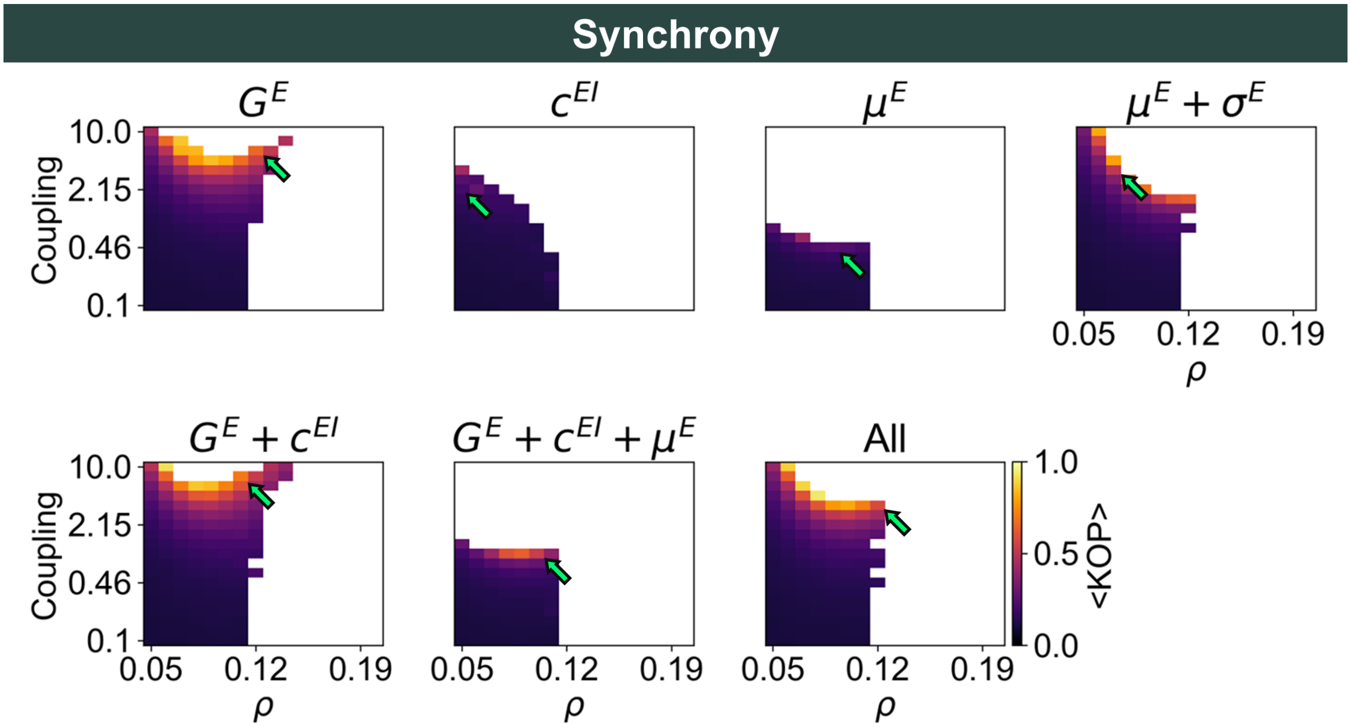

Fig. S20. Parameter spaces representing network synchrony (average Kuramoto Order Parameter across time) for each combination of C and ρ, averaged across mean delays, for models with distinct mechanisms of homeostasis

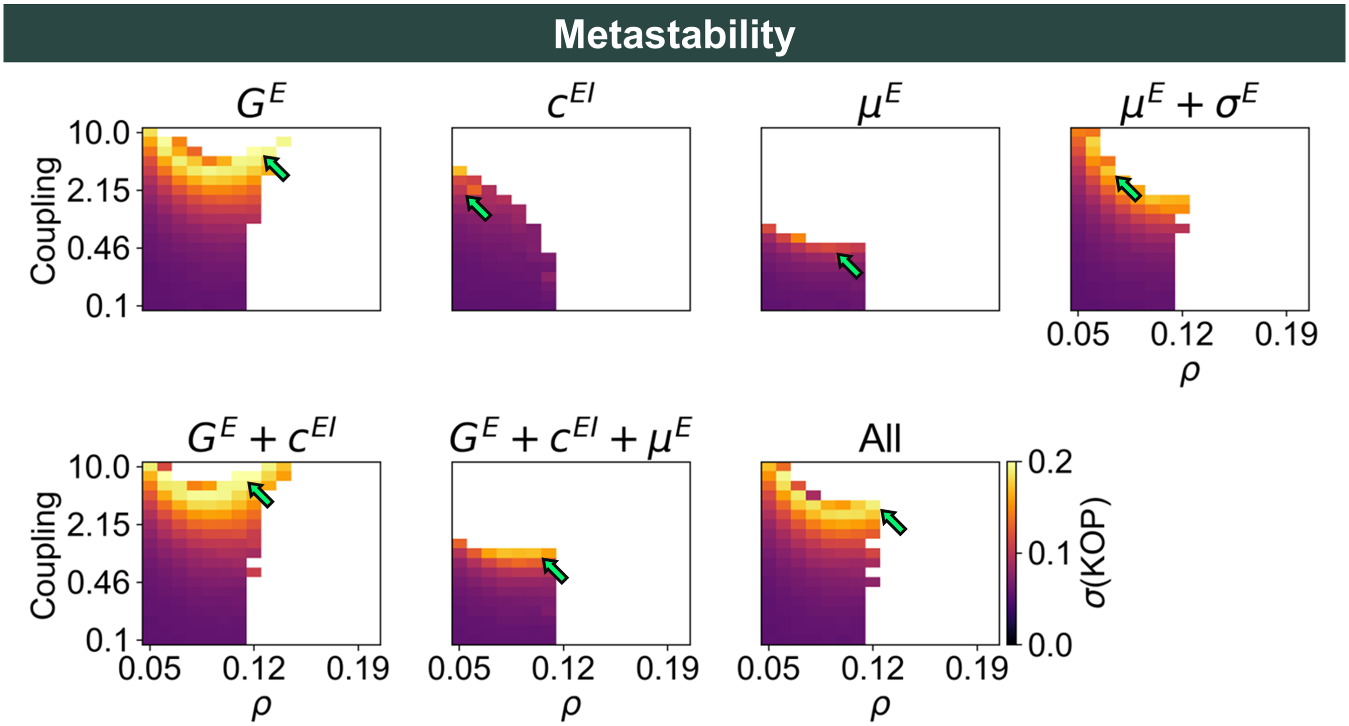

Fig. S21. Parameter spaces representing network metastability (standard deviation of Kuramoto Order Parameter across time) for each combination of C and ρ, averaged across mean delays, for models with distinct mechanisms of homeostasis

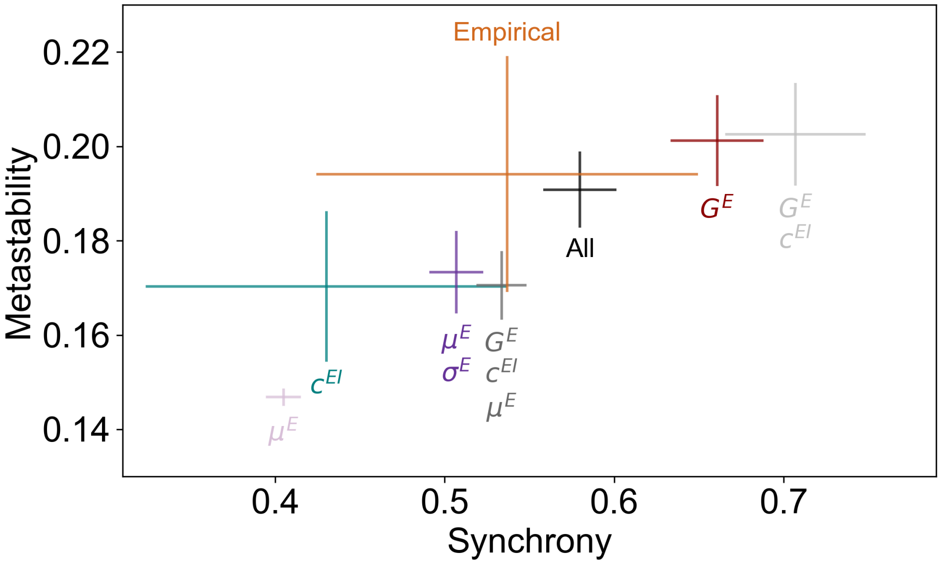

Fig. S22. Synchrony and metastability in models with distinct mechanisms of homeostasis and empirical data The center of the crosses represents the mean synchrony and metastability in models at the optimal working point (C and ρ) of each mechanism of homeostasis. Conversely, horizontal and vertical bars represent the standard deviation of synchrony and metastability, respectively. Note that the model with all mechanisms of homeostasis is the one that better approximates empirical levels of both synchrony and metastability.

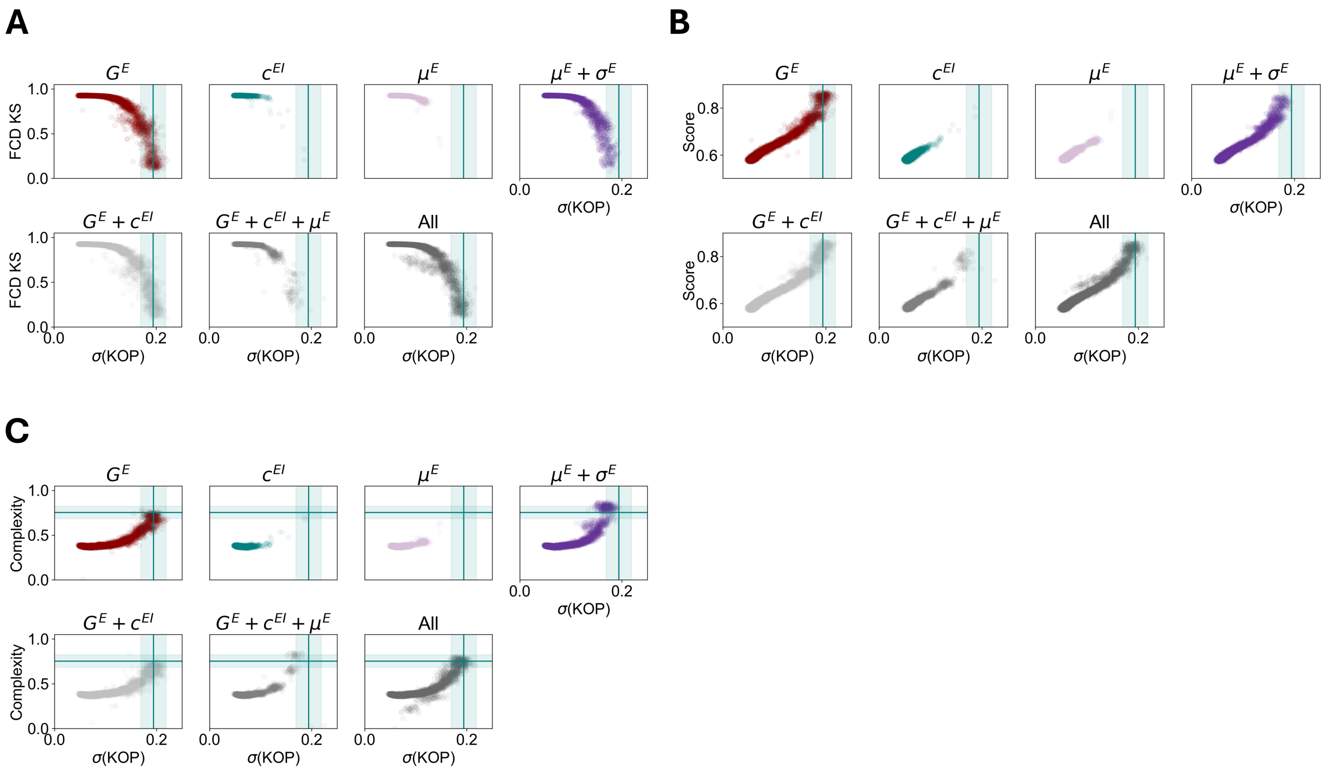

Fig. S23. Relationship between metastability, model performance and complexity for all mechanisms of homeostasis (A) Relationship between metastability and the KS distance between empirical and simulated FCD distributions (B) Relationship between metastability and fitting scores (C) Relationship between metastability and functional complexity. Each data point corresponds to a single simulation. Blue lines and shaded areas represent the mean and standard deviation of metastability and complexity in empirical data.

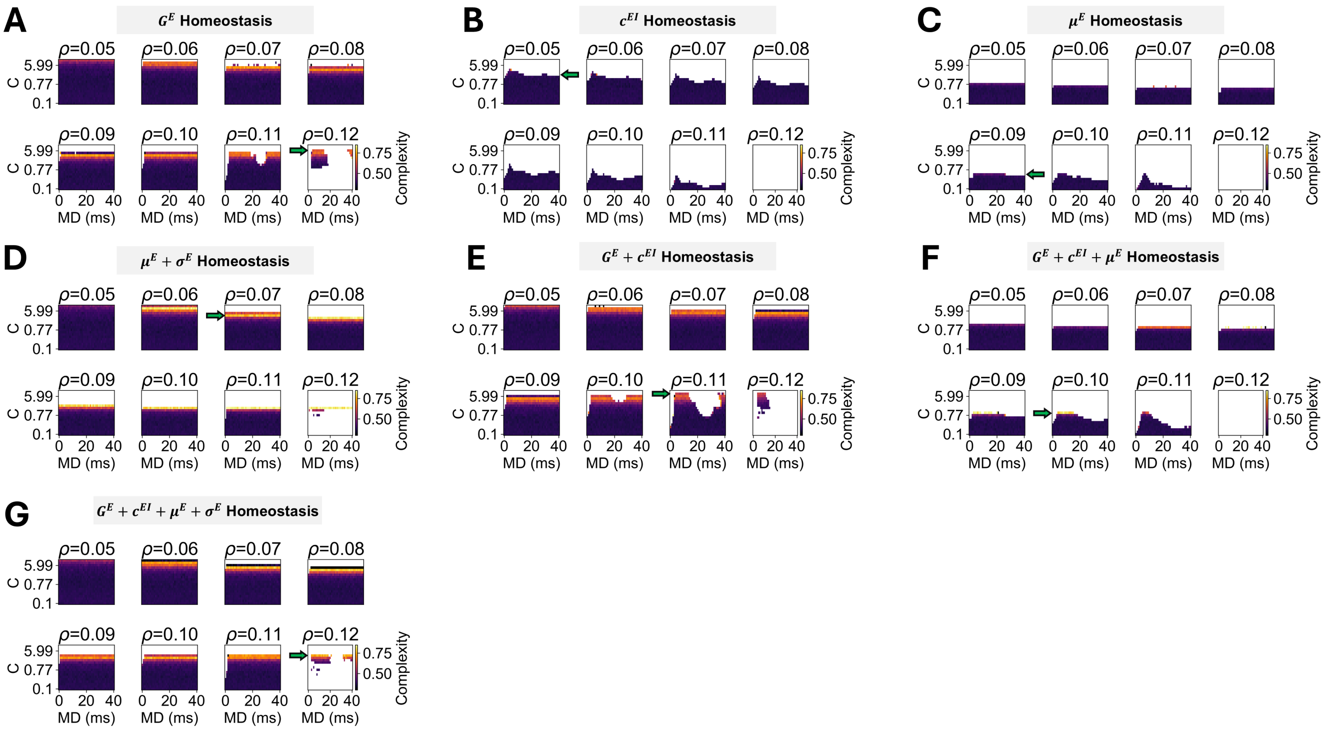

Fig. S24. Functional complexity of models with different mechanisms of homeostasis (A) G^E Homeostasis (B) c^{EI} Homeostasis (C) μ^E Homeostasis (D) $\mu^E + \sigma^E$ Homeostasis (E) $G^E + c^{EI}$ Homeostasis (F) $G^E + c^{EI} + \mu^E$ Homeostasis (G) $G^E + c^{EI} + \mu^E + \sigma^E$ Homeostasis. Colors represent the complexity of FC matrices for each combination of C and ρ.

Functional Complexity

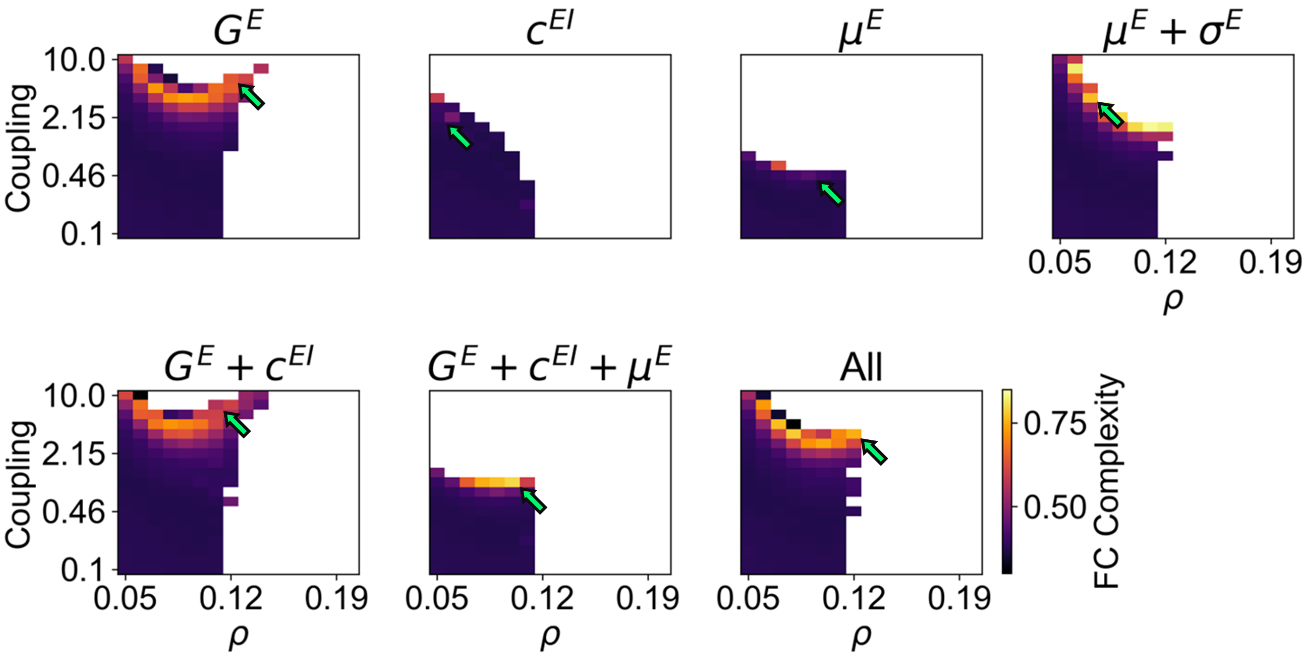

Fig. S25. Parameter spaces representing functional complexity for each combination of C and ρ, averaged across mean delays, for models with distinct mechanisms of homeostasis

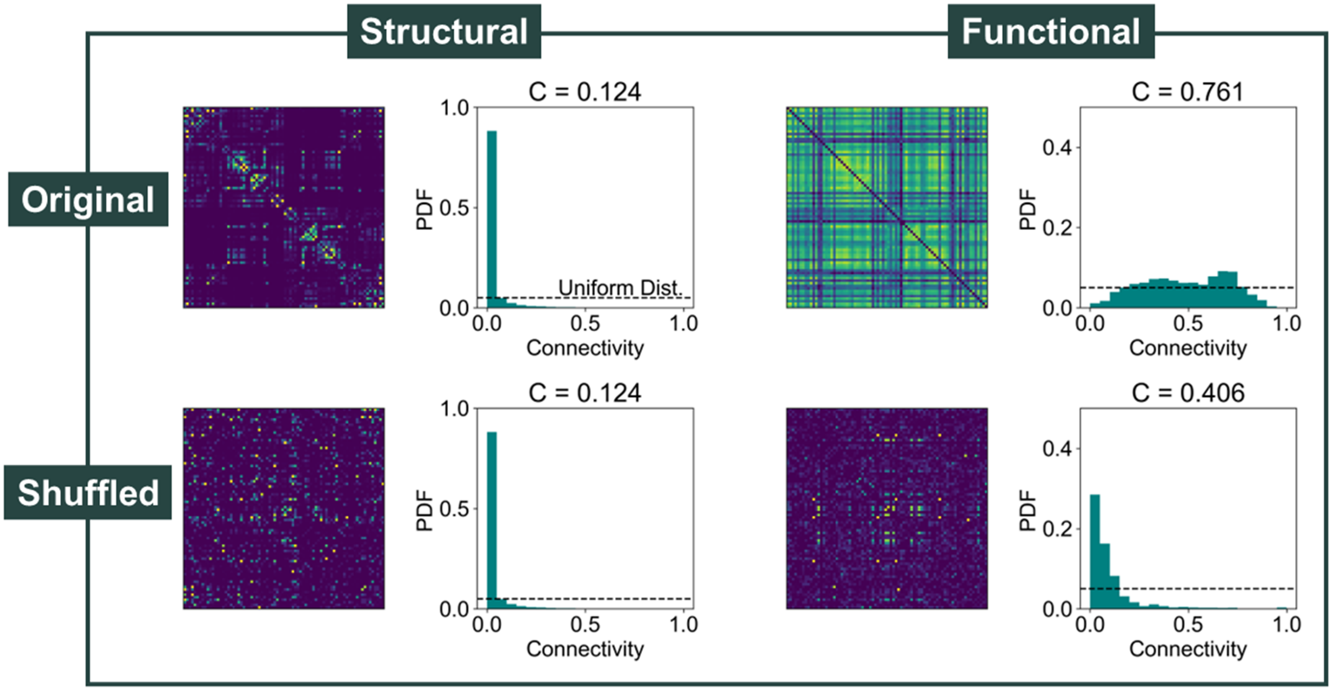

Fig. S26. Functional complexity in models with the original and shuffled connectome. (Left) Structural connectivity matrices and respective weight distributions and complexity, corresponding to the original and shuffled connectomes. (Right) Functional connectivity matrices generated by models with the homeostasis of G^E, c^{EI}, μ^E, and σ^E with the original and shuffled connectomes and respective weight distributons. We used the optimal models ($C = 3.59$, $\rho = 0.12$) with a mean delay of 40 ms.

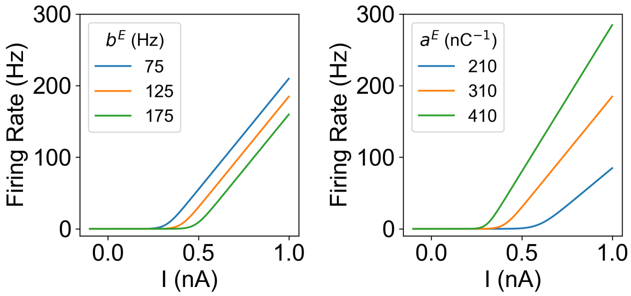

Fig. S27. Input-Output function of the Wong-Wang model with different parameters. Value of $F^E(x)$ in the Wong-Wang model for different values of the firing threshold b^E (Left) and slope a^E (Right)

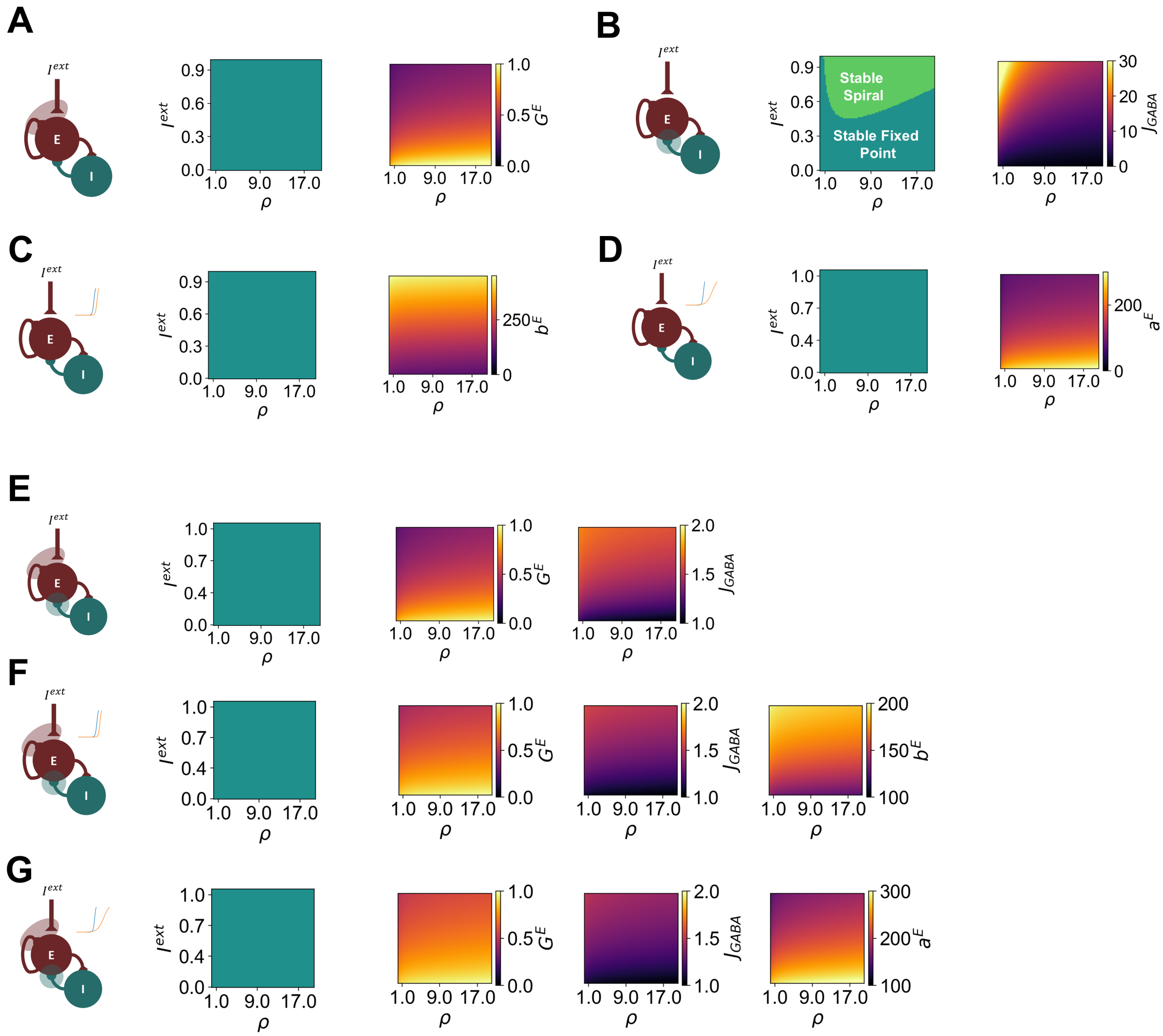

Fig. S28. Dynamics of local Wong-Wang circuits under different mechanisms of E-I homeostasis. We present results for circuits under the homeostasis of excitation (A), inhibition (B), firing threshold (C), firing slope (D), excitation and inhibition (E), excitation, inhibition and firing threshold (F), and excitation, inhibition and firing slope (G). In each subplot, we present the result of linear stability analysis on the left, with colors representing the different dynamical regimes. In addition, we present the values of the parameters being modulated by E-I homeostasis. Note that, in the Wong-Wang model, E-I homeostasis does not have a strong effect on local dynamics, since they remain in a stable regime for all tested combinations of parameters and homeostatic mechanisms. As opposed to the Wilson-Cowan model, there is no Hopf-bifurcation.

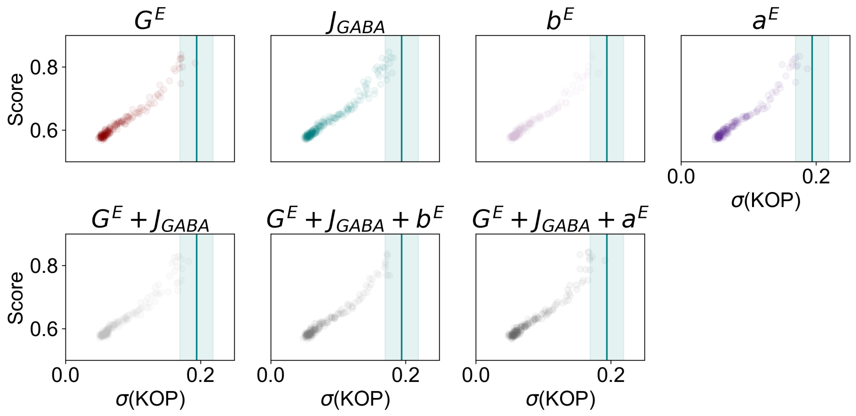

Fig. S29. Relationship between metastability and model performance for all mechanisms of homeostasis in the Wong-Wang model Blue lines and shaded areas represent the mean and standard deviation of metastability in empirical data.

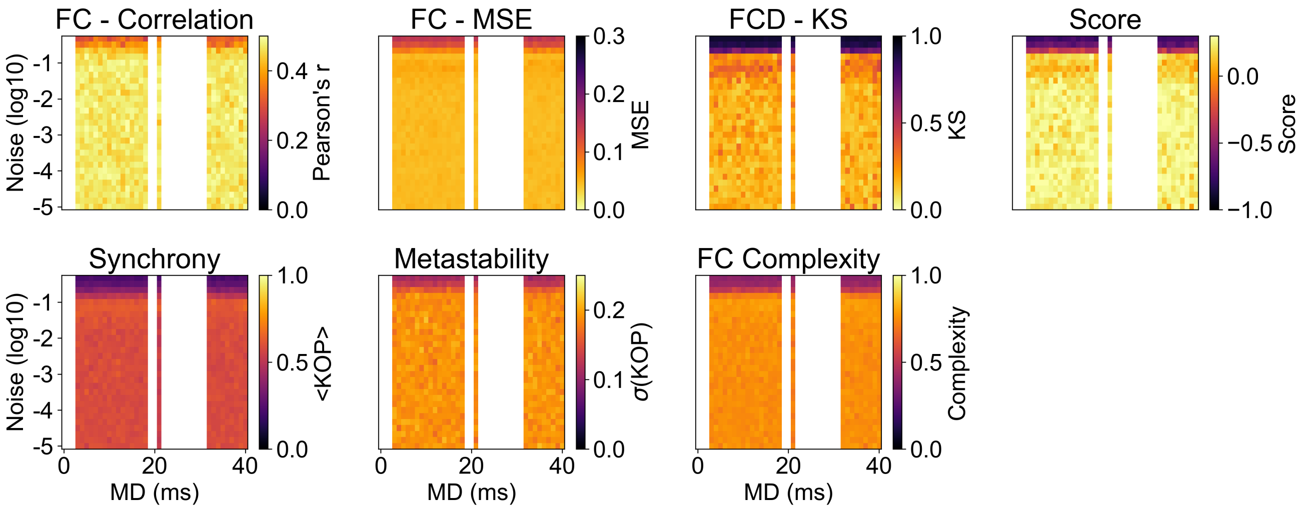

Fig. S30. Parameter space of model with all mechanisms of homeostasis with different noise levels. We present the results for all combinations of mean delay and noise variance for models with $C = 3.59$ and $\rho = 0.12$ and across all mean delays yielding a valid solution of the model. Note that the empty areas in the parameter space relate to mean delays for which $|\langle r^E \rangle - \rho|/\rho > 0.01$ in at least one node.

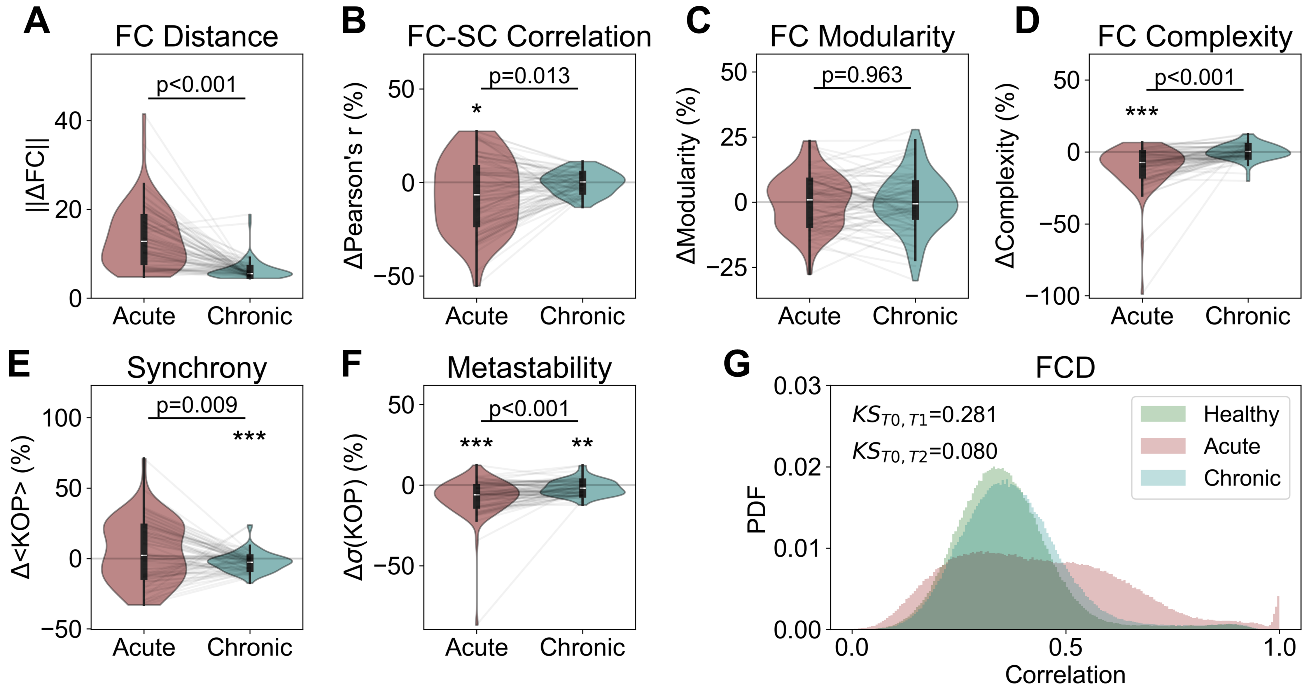

Fig. S31. Disruption and recovery of network properties after focal lesion in models with homeostasis of excitation (G^E) (A) Distance from baseline of FC matrices in acute and chronic simulations (B) Change from baseline (in percentage) of FC-SC correlation in the acute and chronic simulations (C) Same as B, for FC Modularity (D) Same as B, for FC complexity (E) Same as B, for synchrony (D) Same as B, for metastability (F) Distributions of FCD correlations in all stages of our lesion simulations. In addition, we present the Kolmogorov-Smirnov distance between distributions at baseline and acute/chronic simulations. P-values represent the result of a Wilcoxon Ranked-sum test. For 1-sample tests, asterisks represent the significance of a Wilcoxon Ranked sum test (* $p<0.05$; ** $p<0.01$; *** $p<0.001$). All p-values were FDR-corrected.

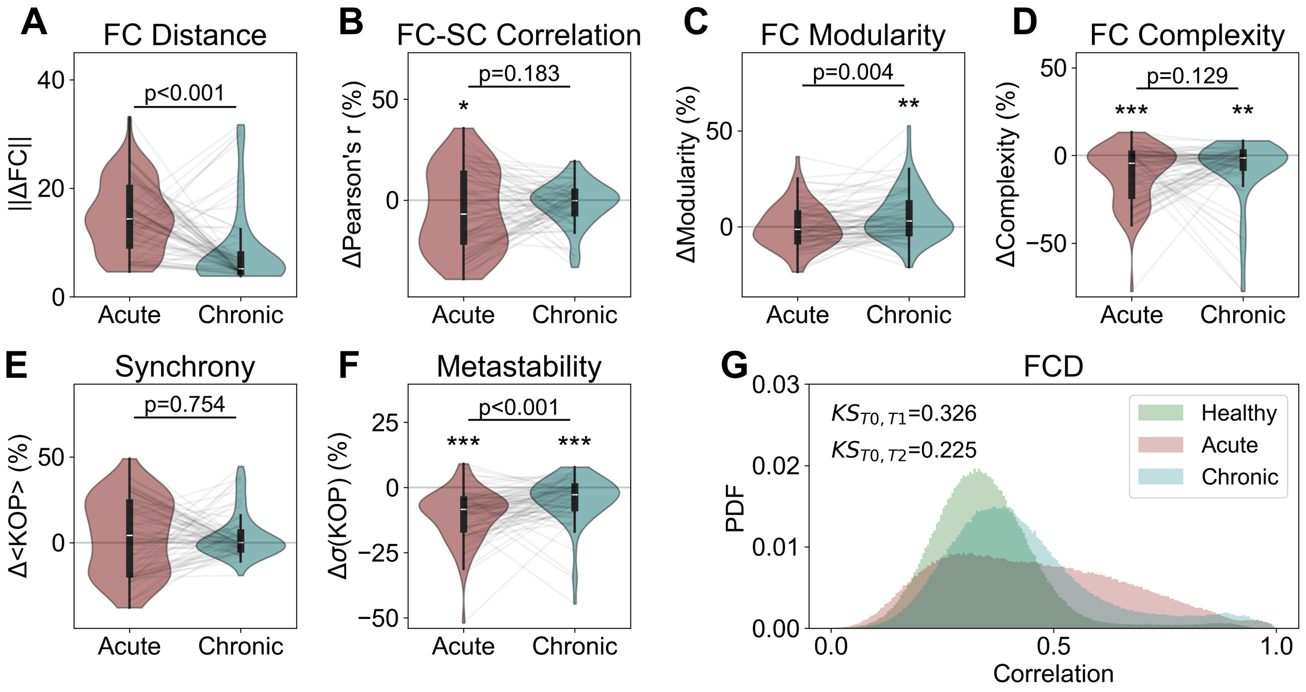

Fig. S32. Disruption and recovery of network properties after focal lesion in models with homeostasis of excitation (G^E) and inhibition (c^{EI}) (A) Distance from baseline of FC matrices in acute and chronic simulations (B) Change from baseline (in percentage) of FC-SC correlation in the acute and chronic simulations (C) Same as B, for FC Modularity (D) Same as B, for FC complexity (E) Same as B, for synchrony (D) Same as B, for metastability (F) Distributions of FCD correlations in all stages of our lesion simulations. In addition, we present the Kolmogorov-Smirnov distance between distributions at baseline and acute/chronic simulations. P-values represent the result of a Wilcoxon Ranked-sum test. For 1-sample tests, asterisks represent the significance of a Wilcoxon Ranked sum test (* $p<0.05$; ** $p<0.01$; *** $p<0.001$). All p-values were FDR-corrected.

Table S1. Optimal parameters, fitting scores, and metastability in the Wong-Wang model for each mechanism of E-I homeostasis in comparison to empirical data

Fitting scores correspond to $\frac{r_{FC}-MSE_{FC}-KS_{FCD}-S_{min}}{S_{max}-S_{min}}$, where r_{FC} is the Pearson's correlation between empirical and simulated FC matrices, MSE_{FC} their mean squared error, KS_{FCD} the Kolmogorov-Smirnov distance between FCD distributions and S_{max}/S_{min} the maximum/minimum of $r_{FC} - MSE_{FC} - KS_{FCD}$. Values are presented as mean±sd over 10 simulations. Values in bold represent a significant difference from empirical data (p<0.05, Mann-Whitney U-test, FDR correction)

Homeostatic Mechanism	C	ρ (Hz)	Score	r_{FC}	MSE_{FC}	KS_{FCD}	Metastability
G^E	0.125	4.0	0.831±0.007	0.41±0.01	0.066±0.002	0.19±0.04	**0.170±0.005**
J_{GABA}	0.261	5.0	0.826±0.010	0.43±0.01	0.063±0.002	0.21±0.03	**0.169±0.006**
b^E	0.178	10.0	0.825±0.006	0.39±0.01	0.068±0.002	0.20±0.03	**0.165±0.006**
a^E	0.261	3.0	0.833±0.007	0.41±0.01	0.067±0.003	0.18±0.03	**0.172±0.006**
$G^E + J_{GABA}$	0.178	6.0	0.820±0.012	0.41±0.02	0.073±0.004	0.24±0.05	**0.164±0.006**
$G^E + J_{GABA} + b^E$	0.681	1.0	0.833±0.008	0.43±0.01	0.068±0.005	0.20±0.04	**0.174±0.006**
$G^E + J_{GABA} + a^E$	0.215	4.0	0.828±0.007	0.40±0.01	0.067±0.002	0.19±0.02	**0.171±0.008**
Empirical	-	-	-	-	-	-	0.194±0.025

Table S2. Default Parameters of the Wilson-Cowan Model

Model Parameters	
c^{EE}	3.5
c^{IE}	3.75
c^{EI}	2.5
G^{E}	1
μ^{E}/μ^{I}	1
σ^{E}/σ^{I}	0.25
τ^{E}	2.5 ms
τ^{I}	5.0 ms

Table S3. Default Parameters of the Wong-Wang Model

Model Parameters		
w_+	1.4 (a.u.)	Local excitatory recurrence
J_{NMDA}	0.15 (nA)	Excitatory synaptic coupling
J_{GABA}	1.00 (nA)	Inhibitory synaptic coupling
G^E	1.00 (a.u)	Excitatory input gain
γ	0.641 (a.u)	Kinetic parameter
I_0	0.382 (nA)	Background input
w^E	1.0 (a.u)	Relative weight of background input to E population
w^I	0.7 (a.u)	Relative weight of background input to I population
a^E	310 (nC^{-1})	Gating variables of $F^{E/I}(x)$
a^I	615 (nC^{-1})	...
b^E	125 (Hz)	...
b^I	177 (Hz)	...
d^E	0.16 (s)	...
d^I	0.087 (s)	...
$\tau^E = \tau^{NDMA}$	100 (ms)	Time constant of E population
$\tau^I = \tau^{GABA}$	10 (ms)	Time constant of I population

References

1. F Páscoa dos Santos, PFMJ Verschure, Excitatory-inhibitory homeostasis and bifurcation control in the Wilson-Cowan model of cortical dynamics. *PLOS Comput. Biol.* **21**, e1012723 (2025) Publisher: Public Library of Science.
2. NS Desai, LC Rutherford, GG Turrigiano, Plasticity in the intrinsic excitability of cortical pyramidal neurons. *Nat. Neurosci.* **2**, 515–520 (1999).
3. K Nataraj, N Le Roux, M Nahmani, S Lefort, G Turrigiano, Visual deprivation suppresses L5 pyramidal neuron excitability by preventing the induction of intrinsic plasticity. *Neuron* **68**, 750–762 (2010).
4. W Wen, GG Turrigiano, Developmental Regulation of Homeostatic Plasticity in Mouse Primary Visual Cortex. *The J. Neurosci.* **41**, 9891–9905 (2021).
5. EM Izhikevich, *Dynamical Systems in Neuroscience: The Geometry of Excitability and Bursting.* (The MIT Press), (2006).
6. W Gerstner, WM Kistler, R Naud, L Paninski, *Neuronal Dynamics: From Single Neurons to Networks and Models of Cognition.* (Cambridge University Press, Cambridge), (2014).
7. F Páscoa dos Santos, J Vohryzek, PFMJ Verschure, Multiscale effects of excitatory-inhibitory homeostasis in lesioned cortical networks: A computational study. *PLOS Comput. Biol.* **19**, e1011279 (2023) Publisher: Public Library of Science.
8. A Vattikonda, BR Surampudi, A Banerjee, G Deco, D Roy, Does the regulation of local excitation-inhibition balance aid in recovery of functional connectivity? A computational account. *NeuroImage* **136**, 57–67 (2016).
9. J Zhang, et al., Disrupted structural and functional connectivity networks in ischemic stroke patients. *Neuroscience* **364**, 212–225 (2017).
10. C Gratton, EM Nomura, F Pérez, M D'Esposito, Focal brain lesions to critical locations cause widespread disruption of the modular organization of the brain. *J. Cogn. Neurosci.* **24**, 1275–1285 (2012).
11. JS Siegel, et al., Re-emergence of modular brain networks in stroke recovery. *Cortex; a J. Devoted to Study Nerv. Syst. Behav.* **101**, 44–59 (2018).
12. MEJ Newman, Fast algorithm for detecting community structure in networks. *Phys. Rev. E* **69**, 066133 (2004).
